# Computational study of the rate constants and free energies of intramolecular radical addition to substituted anilines

**DOI:** 10.3762/bjoc.9.185

**Published:** 2013-08-08

**Authors:** Andreas Gansäuer, Meriam Seddiqzai, Tobias Dahmen, Rebecca Sure, Stefan Grimme

**Affiliations:** 1 Kekulé-Institut für Organische Chemie und Biochemie der Rheinischen-Friedrich-Wilhelms-Universität Bonn, Gerhard-Domagk-Straße 1, D-53121 Bonn, Germany; 2 Mulliken Center for Theoretical Chemistry, Institut für Physikalische und Theoretische Chemie der Rheinischen-Friedrich-Wilhelms-Universität Bonn, Beringstraße 4, D-53115 Bonn, Germany

**Keywords:** aromatic substitution, computational chemistry, DFT-D3, free radical, polar effects, radical reaction

## Abstract

The intramolecular radical addition to aniline derivatives was investigated by DFT calculations. The computational methods were benchmarked by comparing the calculated values of the rate constant for the 5-*exo* cyclization of the hexenyl radical with the experimental values. The dispersion-corrected PW6B95-D3 functional provided very good results with deviations for the free activation barrier compared to the experimental values of only about 0.5 kcal mol^−1^ and was therefore employed in further calculations. Corrections for intramolecular London dispersion and solvation effects in the quantum chemical treatment are essential to obtain consistent and accurate theoretical data. For the investigated radical addition reaction it turned out that the polarity of the molecules is important and that a combination of electrophilic radicals with preferably nucleophilic arenes results in the highest rate constants. This is opposite to the Minisci reaction where the radical acts as nucleophile and the arene as electrophile. The substitution at the N-atom of the aniline is crucial. Methyl substitution leads to slower addition than phenyl substitution. Carbamates as substituents are suitable only when the radical center is not too electrophilic. No correlations between free reaction barriers and energies (Δ*G*^‡^ and Δ*G*_R_) are found. Addition reactions leading to indanes or dihydrobenzofurans are too slow to be useful synthetically.

## Introduction

**Scheme 1:**
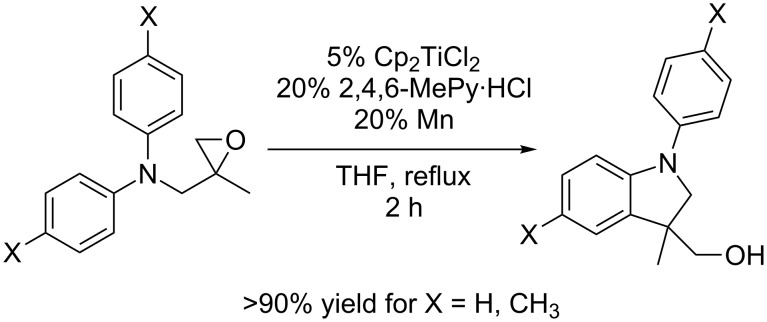
Experimental results for the radical arylation of epoxides.

The development of efficient catalytic reactions is one of the central issues of chemistry [1–2]. Radical-based transformations are amongst the most attractive methods for the use in catalytic cycles due to the ease of radical generation, high functional group tolerance, and selectivity in C–C bond formation [3–5]. Recently, we have reported a novel catalytic reaction, a radical arylation of epoxides [6–8] proceeding via catalysis in single electron steps (for experimental results see Scheme 1) [9–10]. The C–C bond forming step of the catalytic cycle is an intramolecular alkyl radical addition to substituted anilines. Even though only rarely used, reaction sequences employing such steps in an intermolecular or intramolecular manner have been employed in some transformations that are highly useful. Prominent examples are the Minisci reaction [11–15] for the preparation of mainly nitrogen heterocycles and Zard’s homolytic substitution reactions at nitrogen heterocycles with xanthates as radical precursors [16–20].

Despite this usefulness only few studies have been concerned with the determination of absolute rate constants of radical additions to arenes. These were carried out in mechanistic studies of the Minisci reaction [21–22]. It was found that the butyl radical adds to benzene with a rate constant of 3.8 × 10^2^ M^−1^ s^−1^ at 79 °C. In this study it was also demonstrated that the rate constants for addition reactions to electron deficient (protonated) heteroarenes can be much higher due to polar effects. Despite these insightful investigations a more general picture of the kinetics of radical addition to arenes is still elusive and, to the best of our knowledge, thermodynamic data (free energies) for radical additions to arenes are not available.

In this study, we have investigated the rate constants and free energies of intramolecular radical addition to substituted anilines that constitutes the C–C bond forming event of indoline synthesis via homolytic substitution with computational methods. The results are of general interest for the understanding of radical addition to electron rich arenes and should be helpful in the design of novel radical reactions.

The aim of theoretical thermochemistry is to describe the energetics of a chemical process with an accuracy of 1 kcal mol^−1^ or even better. At the same time, the methods applied should not be too demanding in terms of computational costs in order to be still applicable to chemically interesting systems. Kohn–Sham density functional theory (KS-DFT) has been proven to yield good accurate thermochemical properties within acceptable computation times [23–25]. However, the number of the proposed approximate exchange–correlation functionals to choose from is huge and they can suffer from severe problems, e.g., self-interaction error (SIE) leading to underestimated reaction barriers and the lack of long-range electron correlation (London dispersion) effects. Regarding the latter problem, one of the most successful and widely used dispersion correction schemes is DFT-D3, in which a damped, atom-pair wise potential is added to a standard DFT result [26]. A thorough energy benchmark study of various density functionals for general main group thermochemistry, kinetics and non-covalent interactions (GMTKN30 benchmark set) [27] showed that Zhao and Truhlar’s PW6B95 functional [28] in combination with DFT-D3 (termed PW6B95-D3 from now on) is the most robust and accurate general purpose hybrid functional and is therefore used also in this work. As a meta-hybrid functional it partially avoids the SIE by admixture of non-local Fock-exchange (28%) leading to reasonable reaction barriers [27] which are normally underestimated (in particular for radical species) with semi-local GGAs.

We conducted a DFT study using the above mentioned state-of-the-art quantum chemical methods which are applied successfully to various thermochemical problems in our group since several years. This well established protocol consists of gas phase structure optimization at the dispersion-corrected DFT-D3 level using large triple-zeta AO basis sets (TPSS-D3/def2-TZVP) followed by accurate single-point energy calculations at the meta-hybrid level with a further extended AO basis set (PW6B95-D3/def2-QZVP), thermo-statistical corrections from energy to free energy at a given temperature and corrections for solvation free energy by the reliable (DFT-based) COSMO-RS continuum solvation model [29–30]. For recent applications of this procedure see [31–33]. The estimated accuracy is 1–2 kcal mol^−1^ for absolute free enthalpies and relative values for different compounds (trends) should have an error <1 kcal mol^−1^.

## Results and Discussion

### Theoretical methods and benchmarking

#### Computational details

The quantum chemical calculations have been performed with the TURBOMOLE 6.4 suite of programs [34]. All geometry optimizations were performed on the DFT level using the TPSS density functional [35] along with the polarized triple-zeta def2-TZVP basis set [36]. This choice avoids major basis set superposition errors (BSSE) without employing counter-poise corrections and gives theoretically consistent energies and structures. Single point energies were obtained on the PW6B95 [28] level together with the extended quadruple-zeta basis set def2-QZVP [36]. For the small benchmark on the 5-*exo* cyclization of the 5-hexenyl radical the functionals BP86 [37–38] and B3LYP [39–41] also have been applied together with the def2-QZVP basis set. CCSD(T) calculations with the def2-TZVPP[36] basis set have been performed and extrapolated to the complete basis set limit (CBS) [42] via MP2 [43] calculations with the def2-TZVPP and def2-QZVPP basis sets. CCSD-F12 [44] calculations with perturbative triples (CCSD-F12(T)) together with the correlation-consistent basis set cc-pVDZ-F12 [43] for explicitly correlated wave function methods have been calculated using TURBOMOLE 6.5 [45].

For all calculations the resolution-of-identity (RI) approximation for the Coulomb integrals [46] with matching default auxiliary basis sets [47] was applied. The numerical quadrature grid *m4* was employed for integration of the exchange-correlation contribution. For all geometry optimizations as well as single-point calculations the D3 dispersion correction scheme [26] applying Becke–Johnson (BJ) damping [48–50] was used.

Computations of the harmonic vibrational frequencies were performed analytically using the TURBOMOLE module *aoforce*. Thermal corrections from energy to free enthalpy were calculated within the standard harmonic-oscillator approximation for each molecule in the gas phase. The vibrational frequencies were used unscaled. The HOMO–SOMO energy gaps were evaluated using the TPSS-D3/TZVP orbitals. The COSMO-RS model [29–30] was used as implemented in COSMO therm [51] to obtain all solvation free energies. Single point calculations employing the default BP86 [37–38]/def-TZVP [52] level of theory were performed on the optimized gas phase geometries. The solvation contribution was then added to the gas phase free energies.

#### The 5-*exo* cyclization as benchmark

**Scheme 2:**
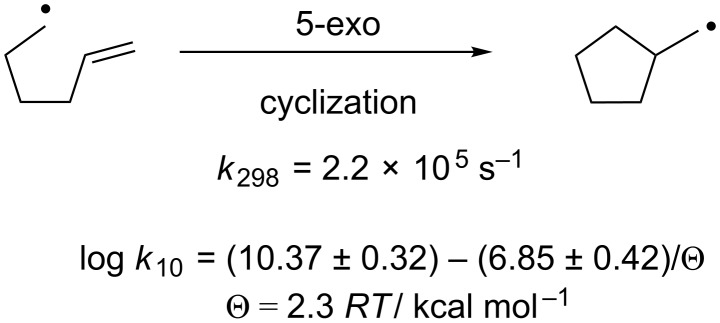
5-*exo* cyclization of the hexenyl radical.

In order to find reliable computational methods for the description of the radical additions, we sought for systems for benchmarking that are structurally related to our system and that are experimentally well investigated. An ideal radical reaction in this respect is the 5-*exo* cyclization (Scheme 2) of the 5-hexenyl radical.

The reaction is preparatively highly important and has been used in many syntheses of complex molecules [3–5]. Moreover, the kinetics of the 5-*exo* cyclization has been studied very thoroughly and the rate constant has been determined by a number of approaches. The value currently accepted as ‘best’ for the rate constant is *k* = 2.2 × 10^5^ s^−1^ at 25 °C [53]. The Arrhenius equation for the 5-hexenyl cyclization has been determined to be log *k*_10_/s^−1^ = (10.37 ± 0.32) − (6.85 ± 0.42)/θ with θ = 2.3 *RT*/ kcal mol^−1^. This implies that *k* lies between 5.1 × 10^4^ s^−1^ and 9.3 × 10^5^ s^−1^ at 25 °C and 6.1 × 10^5^ s^−1^ and 8.2 × 10^6^ s^−1^ at 100 °C.

Moreover, the geometry of the transition state of the 5-*exo* cyclization has been the subject of investigation [54–55]. In the transition state, the length of the forming C–C bond is assumed to be 2.341 Å and the attack of the radical at the olefin occurs at an angle of 105.8°. This value is close to the tetrahedral angle and the Bürgi–Dunitz angle [56].

In this study, the absolute free energy of activation as well as the free energy of the 5-*exo* cyclization of the hexenyl radical were calculated using the TPSS and PW6B95 functionals as described in computational details. From these values the absolute rate constants at 298 K (25 °C) in benzene were computed as summarized in Table 1 together with the experimental value for *k* at 25 °C. The experimental free enthalpy of activation was derived from the rate constant. We furthermore give zero-point and solvation exclusive pure electronic activation energies which are more convenient for a straightforward comparison of theoretical methods.

**Table 1:** Experimental and computed rate constants and free energies of activation of the 5-*exo* cyclization of the hexenyl radical in benzene at 25 °C.

	Δ*Ε* [kcal mol^−1^]	Δ*G*^‡^ [kcal mol^−1^]	*k* [s^−1^]

Experiment		10.18	2.20 × 10^5^
TPSS-D3	4.60	8.07	7. 83 × 10^6^
BP86-D3	4.05	7.52	1.99 × 10^7^
B3LYP	9.81	13.28	1.18 × 10^3^
B3LYP-D3	7.46	10.94	6.18 × 10^4^
PW6B95	7.86	11.33	3.19 × 10^4^
PW6B95-D3	7.16	10.63	1.03 × 10^5^
CCSD(T)/CBS	9.46	12.93	2.12 × 10^3^
CCSD-F12(T)	9.51	13.02	1.83 × 10^3^

From our data it is clear that the semi-local TPSS and BP86 functionals strongly underestimate the activation energy of the 5-*exo* cyclization (by about 3 kcal mol^−1^) due to the SIE but this behavior is as expected for functionals of this type. The hybrid functional B3LYP slightly overestimates the activation barrier when the D3 correction is used and highly overestimates the barrier (by about 3 kcal mol^−1^) without the D3 correction. The plain PW6B95 functional without the dispersion correction still overestimates the barrier by 0.7 kcal mol^−1^. However, the use of PW6B95-D3 provided an energy of activation that differs from the experimental value by only 0.5 kcal mol^−1^. This deviation (about 5%) is within the typical error limits of DFT-D3 and the experimental methods. In passing it is noted that the D3-dispersion correction to the barrier even for this relatively small molecule is substantial (decrease by 0.7 kcal mol^−1^ for PW6B95 and 2.4 kcal mol^−1^ for B3LYP, respectively) and quantitative agreement between theory and experiment cannot be obtained with uncorrected standard density functionals. The encouraging observation that two different hybrid density functionals yield the same barrier to within 0.3 kcal mol^−1^ is mainly an effect of the D3-correction (plain PW6B95 and B3LYP differ by 2 kcal mol^−1^). Although the stabilizing influence of intramolecular London dispersion on the transition state due to its ‘closer’ (more dense) structure is partially quenched by solvation, we think that reliable predictions (‘the right answer for the right reason’) can only be achieved when both effects are accounted for by, e.g., the D3 and COSMO-RS models.

Extrapolated CCSD(T)/CBS via MP2/CBS calculations and estimating the basis set limit by explicitly correlated CCSD-F12(T)/cc-pVDZ-F12 yield an almost identical energy of activation of 12.93 and 13.02 kcal mol^−1^, respectively, which is very encouraging. Presently the origin of the difference to the experimental barrier of 2.4 kcal mol^−1^ is not clear to us. We noted some spin contamination of the Hartee–Fock reference wave function for the transition state structure (S^2^ ≈ 1) but it seems unlikely that this influences the highly accurate CCSD(T) calculations so significantly.

We also investigated the influence of the choice of the geometries and vibrations on the energy of activation and optimized the 5-hexenyl radical as well as the transition state also on the B3LYP-D3/def2-TZVP level. The obtained thermal correction to the free energy of activation is 2.44 kcal mol^−1^ compared to the value based on TPSS geometries of 2.84 kcal mol^−1^. Single-point calculations on the PW6B95/def2-QZVP level for the electronic barrier show that this change by 0.4 kcal mol^−1^ is compensated by a higher Δ*E* of 7.58 kcal mol^−1^ compared to 7.16 kcal mol^−1^ for the TPSS geometries. With an almost identical solvation free enthalpy for the activation process (0.62 and 0.64 kcal mol^−1^) the free energy of activation is practically the same for the TPSS structures (10.63 kcal mol^−1^) and the B3LYP geometries (10.67 kcal mol^−1^). The total influence of the geometries and vibrations on Δ*G*^‡^ is therefore small (0.1–0.2 kcal mol^−1^ at most) and this technical detail cannot explain the discrepancy of the CCSD(T) barrier and the experimental value.

The 5-*exo* cyclisation of 5-hexenyl has been studied before using the G3-(MP2)-RAD protocol and a value of 7.6 × 10^4^ s^−1^ was reported for the rate constant at 21 °C [57]. This high-level composite method was designed to yield accurate gas-phase thermochemical data for free radicals [58]. Nevertheless this protocol does not include solvation effects, which might be an explanation for the better agreement of the rate constant presented in this work compared to the experimental value [53]. In order to increase the validity of the benchmarking, the rate constants *k* were calculated for a number of temperatures and compared to the values obtained from the Arrhenius equation reported as most reliable (Table 2).

**Table 2:** Rate constants of the 5-*exo* cyclization of the hexenyl radical in benzene at different temperatures calculated at the PW6B95-D3/QZVP//TPSS-D3/def2-TZVP level of theory.

*T* [°C]	*k*_exp._ [s^−1^]	*k*_calc._ [s^−1^]

25	2.20 × 10^5^	1.02 × 10^5^
40	3.83 × 10^5^	1.99 × 10^5^
60	7.43 × 10^5^	4.36 × 10^5^
80	1.34 × 10^6^	8.76 × 10^5^
100	2.26 × 10^6^	1.62 × 10^6^

The results demonstrate that the agreement between calculated and experimental values is becoming even better with increasing temperature. This suggests that the (small) error in the calculated values is due to a slight overestimation of the enthalpy of activation. When employing the COSMO-RS model to simulate different media (THF and benzene) we found that the rate constant at 40 °C for the two solvents is almost identical (2.02 × 10^5^ s^−1^ and 1.99 × 10^5^ s^−1^). This is in agreement with experimental results indicating the insensitivity of *k* to solvent effects. On an absolute scale, however, inclusion of these effects is important as the free energy barrier computed for the gas phase is increased by about 0.6 kcal mol^–1^ in THF or benzene. This improves the agreement between theory and experiment. As documented in the Supporting Information, even the sign of solvent correction varies for different systems and differences on the order of 1 kcal mol^–1^ are found and it hence can be concluded that they should be included by default in accurate computational work.

Finally, the literature transition state geometry and our geometry are very similar. The length of the forming bond is 2.30 Å and the angle of attack to the double bond is 108.2° in our treatment. These values are slightly different than the values used in the modeling based calculations of radical cyclization (2.34 Å and 105.8°) that were derived from values of the attack of alkyl radicals on ethane and propene, however [54–55].

In summary, it can be concluded that the calculations employing the PW6B95-D3/QZVP//TPSS-D3/TZVP method on the 5-*exo* cyclization of the hexenyl radical are in excellent agreement with the experimental and previous computational results. Therefore, this approach was employed in the investigations of the following intramolecular radical additions to arenes.

### Investigation of the radical addition to substituted anilines

In our preparative work, we have been mostly concerned with the catalytic synthesis of indolines via addition reactions of epoxide derived radicals [59–60] and thus, radical additions to substituted anilines are investigated in this study.

#### Substitution at nitrogen

**Scheme 3:**
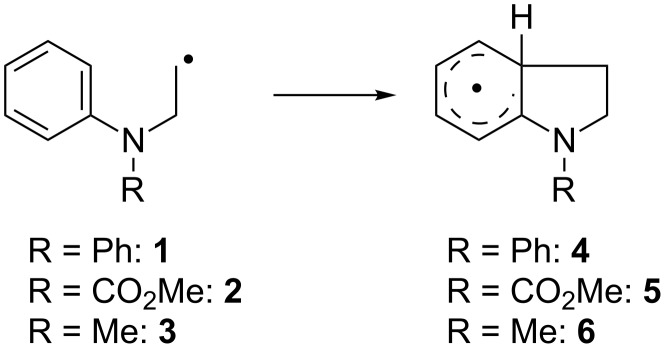
Intramolecular radical additions of simple aniline derivatives.

Radicals **1**–**3** (Scheme 3) are simple models for the addition steps of these sequences and were therefore studied first (Table 3).

**Table 3:** Calculated kinetic and thermodynamic data (on the PW6B95-D3/QZVP//TPSS-D3/def2-TZVP level) and HOMO-SOMO gap Δ*E*_H-S_ (on the TPSS-D3/TZVP level) of the reactions of **1**–**3** in benzene at 40 °C.

Subst.	*k* [s^−1^]	Δ*G*^‡^ [kcal mol^−1^]	Δ*G*_R_ [kcal mol^−1^]	Δ*E*_H-S_ [eV]

**1**	3.56 × 10^3^	13.3	−10.3	−0.77
**2**	5.62 × 10^3^	13.0	−9.9	−1.40
**3**	3.88 × 10^2^	14.7	−10.2	−1.14

Somewhat surprisingly for us, all addition reactions are considerably exergonic and all ∆*G*_R_ values are fairly similar. Thus, radical stabilization in **4**–**6** provides a substantial thermodynamic driving force for the addition. Despite the similarity of the ∆*G*_R_ values the rate constants of the addition differ significantly. For **1** and **3** the difference in *k* can be ascribed to the lower HOMO–SOMO gap and hence more favorable polar effects for **1**. For **2** having the highest rate constant this is not the case. We suggest that the electron withdrawing substituent on N reduces the aromaticity of the aniline and hence facilitates radical attack.

**Scheme 4:**
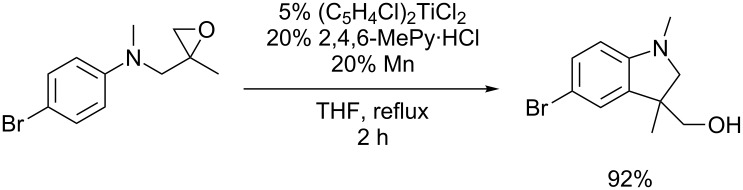
Successful catalytic radical addition to an *N*-methyl substituted aniline.

For **1** and **2** the addition is about as fast as the 6-*endo* cyclization of the hexenyl radical. Such reactions and other even slower cyclizations are well documented in titanocene mediated and catalyzed radical processes [61–69]. Therefore, the relatively high computational rate constant for the addition of **1** readily explains the excellent synthetic results with epoxides derived from aryl substituted anilines in the radical arylation of epoxides. The reaction of **3** is too slow to be useful in typical radical chain reactions. However, reactions under our catalytic reaction conditions [6] with radicals similar to **3** were successful, too (see Scheme 4). Nevertheless, the transformations are, in agreement with the calculations, clearly more demanding than those with radicals similar to **1** and thus, more elaborate catalysts and the use of additives to enhance catalyst stability is essential.

**Figure 1:**
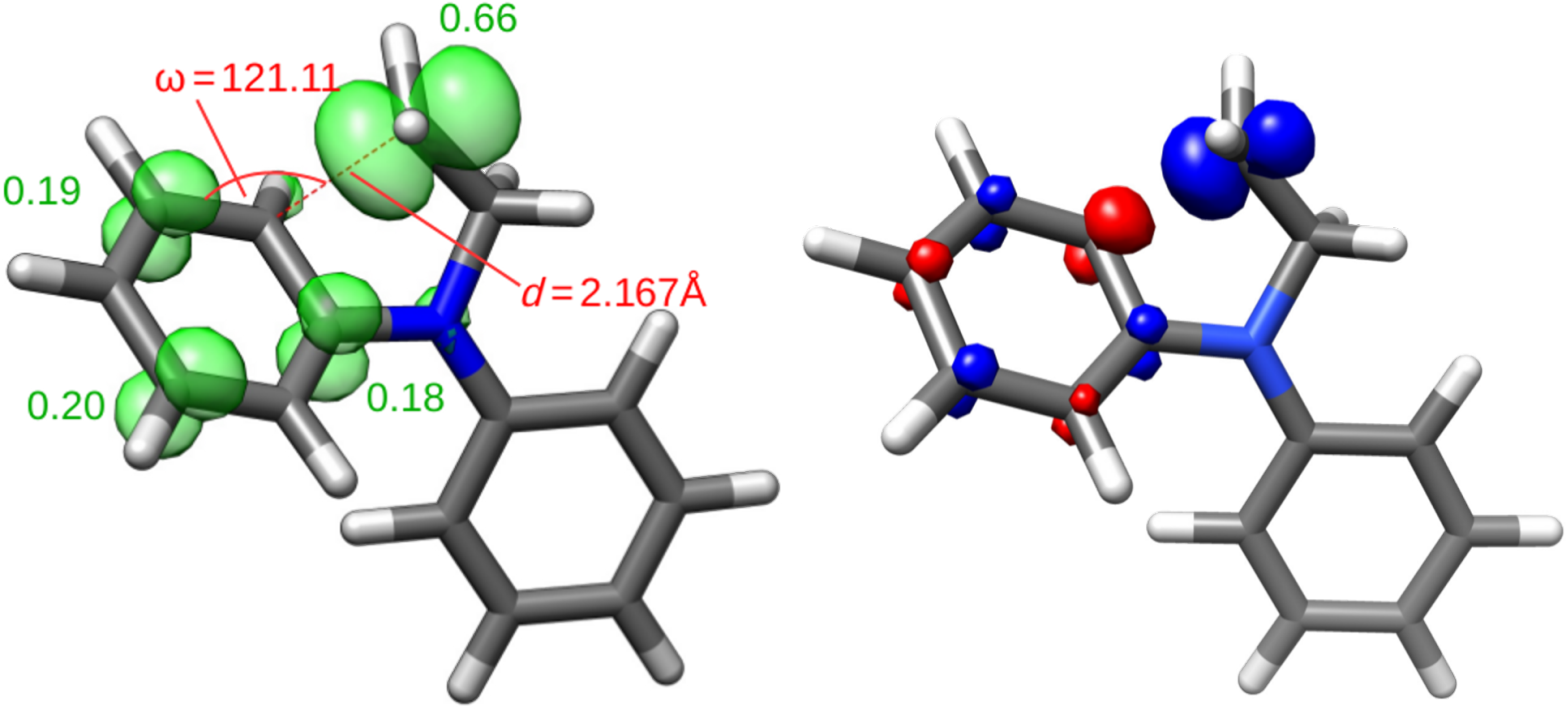
Optimized structure of the transition state of the radical addition of **1** (left: spin density plot and atomic spin-density populations; right: SOMO).

In accordance with intuition, all transition states are ‘later’ than that of the 5-*exo* cyclization as indicated by the shorter distances between the radical center and the C-atom attacked for **1**–**3** (2.15–2.17 Å vs 2.30 in the 5-*exo* cyclization). Moreover, the trajectory of attack on the arene is very similar for **1**–**3** (121–123°). This angle is substantially larger than for the 5-*exo* cyclization (108.2°). This is shown for the addition of **1** in Figure 1.

#### Substitution at the radical center

**Scheme 5:**
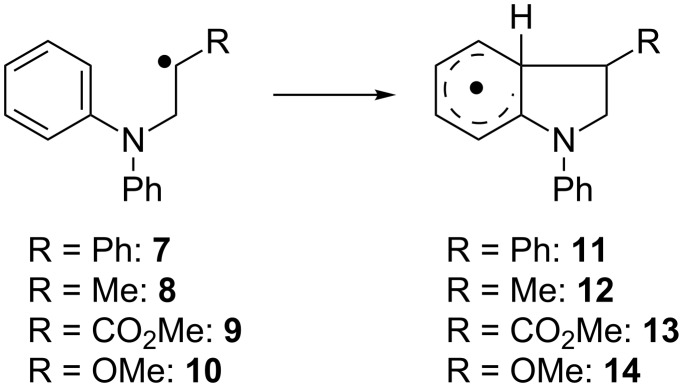
Intramolecular radical additions of simple aniline derivatives.

We investigated the influence of radical substitution on the rate and the free energy of the addition reaction next. In order to ensure comparability the examples were chosen with phenyl substitution at N. They are shown in Scheme 5 and the results are summarized in Table 4.

**Table 4:** Calculated kinetic and thermodynamic data (on the PW6B95-D3/QZVP//TPSS-D3/def2-TZVP level) and HOMO–SOMO gap Δ*E*_H-S_ (on the TPSS-D3/TZVP level) of the reactions of **7**–**10** in benzene at 40 °C.

Subst.	*k* [s^−1^]	Δ*G*^‡^ [kcal mol^−1^]	Δ*G*_R_ [kcal mol^−1^]	Δ*E*_H-S_ [eV]

**7**	7	17.2	+2.7	−1.07
**8**	8.94 × 10^2^	14.2	−6.7	−0.90
**9**	2.17 × 10^4^	12.2	−3.3	−0.83
**10**	1.70 × 10^3^	13.8	−3.9	−1.14

**Scheme 6:**
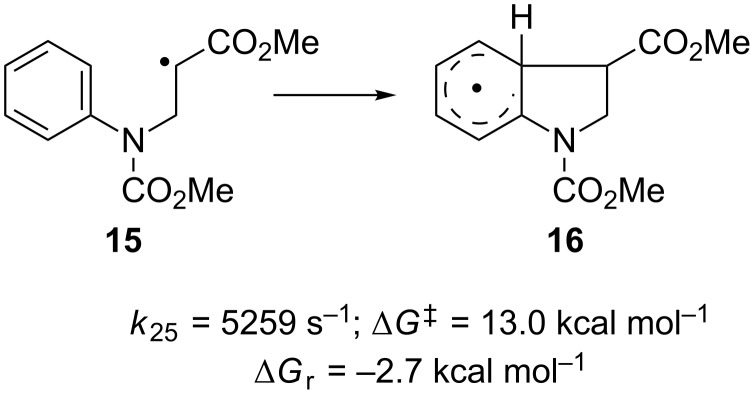
Mismatching of polar effects.

The notion that the radical acts as an electrophile and the arene as nucleophile is further corroborated by the highest rate constant for the addition reaction of **9** that has the most electrophilic radical center due to ester substitution. The more nucleophilic radicals **8** and **10** react slower than **1**. The –OMe group in **10** is a better electron donor than the –Me group in **8**, which should make it more nucleophilic and lead to a slower radical addition. The calculated rate constant for **10** still is higher than for **8**, which leads to the conclusion that the electron withdrawing inductive effect of the –OMe group overcomes its +M-effect. However, the difference in Δ*G*^‡^ is below 0.5 kcal mol^−1^ and thus within the error margin of the theoretical method. The addition of the stabilized benzyl radical is slowest and also endergonic. As above, no correlation between *k* and ∆*G*_R_ is obvious. The polarity of the radical [70–72] and the arene is reversed in comparison with the Minisci reaction [11]. With alkyl substitution at N (as in **3**) similar trends are observed. This indicates that for compounds derived from **3** the SOMO–HOMO interaction is decisive, too. Finally, care has to be taken in transferring effects from one series of substrates to another. As shown in Scheme 6, radical **15** adds to the arene to give **16** substantially slower than **9**. Thus, the combination of an electron deficient radical with an electron withdrawing substitution on N leads to a mismatching of polar effects with respect to *k*.

#### Effect of arene substitution

The results obtained so far strongly suggest that a matching of the nucleophilicity of the arene and the electrophilicity of the radical center are decisive for the magnitude of *k*. We investigated this issue by the introduction of substituents either in the *p*-position or both *m*-positions of the anilines.

#### Effect of *p*-substitution

**Scheme 7:**
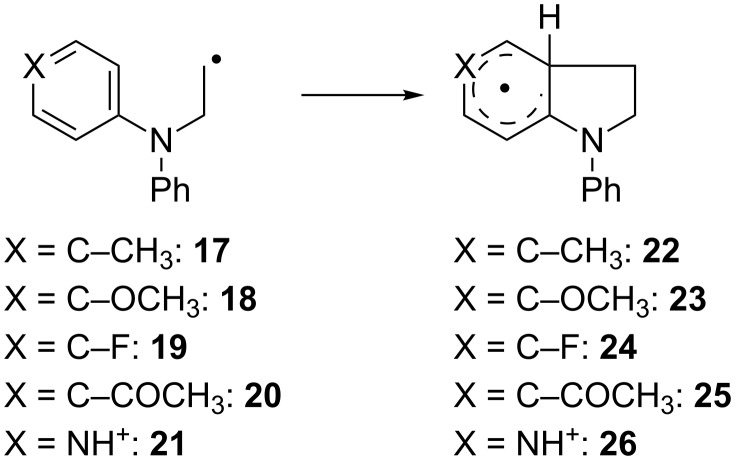
Examples of *p*-substituted anilines investigated.

The examples of *p*-substitution investigated are summarized in Scheme 7 and Table 5.

**Table 5:** Calculated kinetic and thermodynamic data (on the PW6B95-D3/QZVP//TPSS-D3/def2-TZVP level) and HOMO–SOMO gap Δ*E*_H-S_ (on the TPSS-D3/TZVP level) of the reactions of **17**–**21** in benzene at 40 °C.

Subst.	*k* [s^−1^]	Δ*G*^‡^ [kcal mol^−1^]	Δ*G*_R_ [kcal mol^−1^]	Δ*E*_H-S_ [eV]

**17**	2.52 × 10^4^	12.1	−11.2	−0.77
**18**	7.25 × 10^3^	12.8	−9.4	−0.74
**19**	5.54 × 10^3^	13.0	−11.4	−0.70
**20**	9.04 × 10^2^	14.1	−8.7	−0.89
**21**	5.44 × 10^2^	14.5	−8.9	−0.98

Methyl substitution in **17** leads by far to the highest value of *k*. For **18** and **19** higher values than for **1** were obtained. However, the effect of –OCH_3_ and –F substitution is surprisingly small and within the error margin of the computational method. Electron withdrawing substituents (**20** and **21**) strongly retard the addition. In these cases larger SOMO–HOMO gaps are involved. The radicals act as electrophiles in all cases. Thus, compared to the Minisci reaction our addition has a reversed polar effect.

Changing the substituent at N from Ph to CH_3_ leads to similar trends with lower absolute values of *k* as expected. These results are summarized in Supporting Information File 1.

#### Effect of *m*,*m*’-disubstitution

**Scheme 8:**
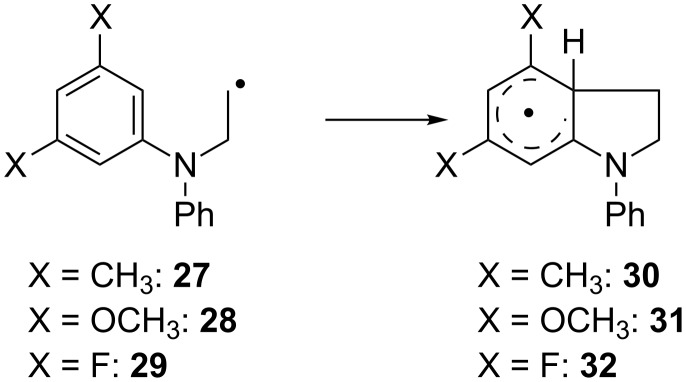
Examples of *m*,*m*’-disubstituted anilines investigated.

The second substitution pattern investigated is the *m*,*m*’-disubstitution. By adding both substituents, the problem of the regioselectivity of addition to the arene is circumvented. The examples and results are summarized in Scheme 8 and Table 6.

**Table 6:** Calculated kinetic and thermodynamic data (on the PW6B95-D3/QZVP//TPSS-D3/def2-TZVP level) and HOMO–SOMO gap Δ*E*_H-S_ (on the TPSS-D3/TZVP level) of the reactions of **27**–**29** in benzene at 40 °C.

Subst.	*k* [s^−1^]	Δ*G*^‡^ [kcal mol^−1^]	Δ*G*_R_ [kcal mol^−1^]	Δ*E*_H-S_ [eV]

**27**	1.01 × 10^4^	12.6	−10.2	−0.80
**28**	5.46 × 10^2^	14.5	−9.8	−1.46
**29**	1.62 × 10^2^	15.2	−8.7	−1.30

As above, methyl substitution (in **27**) leads to a higher value for *k*. The introduction of two –OCH_3_ (in **28**) or two –F substituents (in **29**) results in a reduction of the value of *k* compared to **1**. While this could be indicative of a weak negative inductive effect, the differences in Δ*G*^‡^ are low and within the errors of the computational method.

#### Radical additions leading to dihydrobenzofurans and indanes

**Scheme 9:**
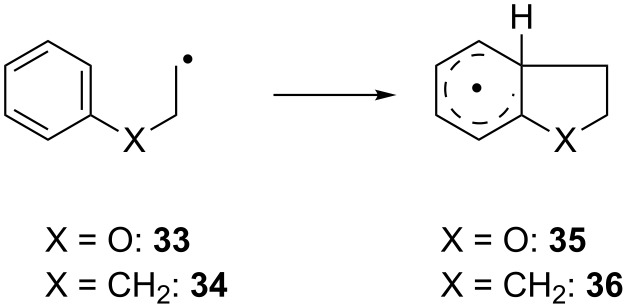
Addition reactions leading to dihydrobenzofuran and an indane.

So far, all radicals investigated contained a substituted aniline and the importance of the nucleophilicity of the arene has become obvious for a number of examples. To conclude our study we therefore investigated an O atom and a CH_2_ group in the chain linking the radical center and the arene as shown in Scheme 9. The results are summarized in Table 7.

**Table 7:** Calculated kinetic and thermodynamic data (on the PW6B95-D3/QZVP//TPSS-D3/def2-TZVP level) and HOMO–SOMO gap Δ*E*_H-S_ (on the TPSS-D3/TZVP level) of the reactions of **33** and **34** in benzene at 40 °C.

Subst.	*k* [s^−1^]	Δ*G*^‡^ [kcal mol^−1^]	Δ*G*_R_ [kcal mol^−1^]	Δ*E*_H-S_ [eV]

**33**	51	15.9	−6.0	−1.61
**34**	2	17.9	−1.8	−2.22

For both **33** and **34** the calculated rate constants are substantially lower than for **1** and **3**. This can be attributed to the much higher SOMO–HOMO gap that indicates much weaker polar effects for the reactions of **33** and **34**. Thermodynamically, both addition reactions are favorable and once again, no correlation between ∆*G*_r_ and *k* is obvious. Thus, for our simple model systems the combination of only weakly nucleophilic arenes and an electrophilic radical center is disadvantageous. This is in agreement with preliminary synthetic results that suggest that dihydrobenzofurans and indanes are not accessible via the titanocene catalyzed radical arylation.

## Conclusion

The intramolecular radical addition to substituted anilines was studied computationally with the aid of the PW6B95-D3 functional in combination with the large quadruple-zeta basis set def2-QZVP. This method was chosen after benchmarking on the 5-*exo* cyclization of the hexenyl radical. It provides sufficiently accurate values for the rate constant of the cyclization over a wide range of temperatures.

For the radical addition to anilines it was found that polar effects are highly important and a combination of electrophilic radicals with preferably nucleophilic arenes results in the highest rate constants. In general, the relative rates correlate with a low SOMO–HOMO gap. Thus, the polarity of the radical and the arene is reversed in comparison with the related Minisci reaction. The substitution at the N-atom of the aniline is crucial. Methyl substitution leads to slower addition than phenyl substitution. Carbamates as substituents are suitable only when the radical center is not too electrophilic. Concerning the substitution pattern of the arene it was found that electron releasing substituents accelerate the addition whereas strongly electron withdrawing substituents like acyl groups retard the addition. Para-substitution has a stronger influence than meta-substitution. Addition reactions leading to indanes or dihydrobenzofurans are too slow to be useful.

## Supporting Information

File 1 Energies and coordinates of all radicals and transition states, tables with data on radicals with Me-substitution on N analogous to Tables 4, 5, and 6.
