# Supplementary material for: Computational study of the rate constants and free energies of intramolecular radical addition to substituted anilines
Source: Beilstein J Org Chem. 2013 Aug 8;9:1620–9. doi: 10.3762/bjoc.9.185 (PMC3778327; doi:10.3762/bjoc.9.185)
Supplement: File 1 — Energies and coordinates of all radicals and transition states, tables with data on radicals with Me-substitution on N analogous to Tables 4, 5, and 6. [file Beilstein_J_Org_Chem-09-1620-s001.pdf]

Supporting Information

for

Computational study of the rate constants and free energies of intramolecular radical addition to substituted anilines

Andreas Gansäuer*[1], Meriam Seddiqzai[1], Tobias Dahmen[1], Rebecca Sure[2] and Stefan Grimme*[2]

Address: [1]Kekulé-Institut für Organische Chemie und Biochemie der Rheinischen-Friedrich-Wilhelms-Universität Bonn, Gerhard-Domagk-Straße 1, D-53121 Bonn, Germany and [2]Mulliken Center for Theoretical Chemistry, Institut für Physikalische und Theoretische Chemie der Rheinischen-Friedrich-Wilhelms-Universität Bonn, Beringstraße 4, D-53115 Bonn, Germany

Email: Andreas Gansäuer - andreas.gansaeuer@uni-bonn.de, Stefan Grimme - grimme@thch.uni-bonn.de

Energies and coordinates of all radicals and transition states, tables with data on radicals with Me-substitution on N analogous to Tables 4, 5, and 6

Table S.1: Calculated kinetic and thermodynamic data on the PW6B95-D3/QZVP//TPSS-D3/def2-TZVP level of the reactions of the 5-hexenyl radical and **1**–**34** in benzene at 40 °C.

	$E^{‡}_{E\text{->}TS}$ kcal × mol^{-1}	thermo-correction kcal × mol^{-1}	solvent-correction kcal × mol^{-1}	$\Delta G^{‡}_{E\text{->}TS}$ kcal × mol^{-1}	k s^{-1}
**5-hexenyl-radical**	7.16	3.02	0.61	10.79	1.99×10^{5}
**1**	10.85	2.19	0.25	13.29	3.56×10^{3}
**2**	10.68	2.17	0.15	13	5.62×10^{3}
**3**	12.19	1.98	0.49	14.66	3.88×10^{2}
**7**	14.73	1.83	0.61	17.16	7
**8**	11.29	2.64	0.22	14.15	8.94×10^{2}
**9**	11	1.35	−0.18	12.16	2.17×10^{4}
**10**	11.54	2.08	0.13	13.75	1.70×10^{3}
**15**	11.83	1.35	−0.13	13.04	5.26×10^{3}
**17**	10.45	1.36	0.25	12.07	2.53×10^{4}
**18**	10.32	2.21	0.31	12.84	7.25×10^{3}
**19**	10.48	2.41	0.12	13.01	5.54×10^{4}
**20**	11.73	1.89	0.52	14.14	9.04×10^{2}
**21**	12.22	2.22	0.01	14.45	5.44×10^{2}
**27**	10.01	2.4	0.22	12.64	1.00×10^{4}
**28**	11.24	2.33	0.08	13.65	1.99×10^{3}
**29**	11.8	2.05	0.44	14.29	7.13×10^{2}
**33**	13.31	2.12	0.5	15.93	51
**34**	15.4	2.18	0.37	17.94	2

All rate constants were calculated using the transition state theory:

$k = k_{B}T / h \exp(-\Delta_{R}G^{‡} / RT)$

Table S.2: Calculated kinetic and thermodynamic data (on the PW6B95-D3/QZVP//TPSS-D3/def2-TZVP level) and the HOMO–SOMO gap $\Delta E_{H\text{-}S}$ (on the TPSS-D3/TZVP level) of the reactions of **S1–S3** in benzene at 40 °C.

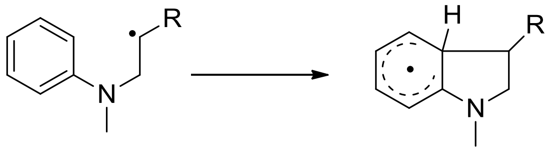

R = Ph: **S1**
R = Me: **S2**
R = CO_2Me: **S3**

R = Ph: **S5**
R = Me: **S6**
R = CO_2Me: **S7**

Subst.	k<br>s^{-1}	$\Delta G^{\ddagger}$<br>kcal × mol^{-1}	ΔG_R<br>kcal × mol^{-1}	$E_{H\text{-}S}$<br>eV
**S1**	0.2	19.56	2.84	−1.36
**S2**	501	14.51	−8.06	−1.24
**S3**	7100	12.86	−2.48	−1.17

Table S.3: Calculated kinetic and thermodynamic data (on the PW6B95-D3/QZVP//TPSS-D3/def2-TZVP level) and the HOMO–SOMO gap $\Delta E_{H\text{-}S}$ (on the TPSS-D3/TZVP level) of the reactions of **S8**–**S11** in benzene at 40 °C.

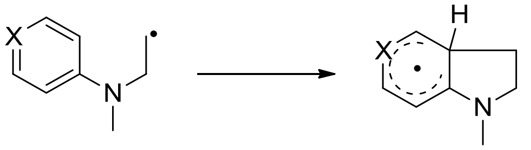

X = C-CH_3: **S8**
X = C-OCH_3: **S9**
X = C-F: **S10**
X = C-$COCH_3$: **S11**
X = NH^+: **S12**

X = C-CH_3: **S13**
X = C-OCH_3: **S14**
X = C-F: **S15**
X = C-$COCH_3$: **S16**
X = NH^+: **S17**

Subs.	k<br>s^{-1}	$\Delta G^{\ddagger}$<br>kcal × mol^{-1}	ΔG_R<br>kcal × mol^{-1}	$E_{H\text{-}S}$<br>eV
**S8**	2401.00	13.53	−11.25	−1.09
**S9**	840.00	14.18	−12.57	−0.84
**S10**	1426.00	13.86	−12.36	−1.00
**S11**	140.00	15.30	−9.00	−1.19
**S12**	439.00	14.59	−10.34	−1.46

Table S.3: Calculated kinetic and thermodynamic data (on the PW6B95-D3/QZVP//TPSS-D3/def2-TZVP level) and the HOMO–SOMO gap $\Delta E_{\text{H-S}}$ (on the TPSS-D3/TZVP level) of the reactions of **S18**–**S20** in benzene at 40 °C.

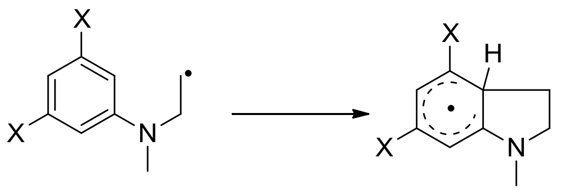

X = CH: **S18**
X = OCH_3: **S19**
X = F: **S20**

X = CH: **S21**
X = OCH_3: **S22**
X = F: **S23**

Subs.	k s^{-1}	$\Delta G^{\ddagger}$ kcal × mol^{-1}	ΔG_R kcal × mol^{-1}	$E_{\text{H-S}}$ eV
**S18**	2188	13.59	−10.71	−1.12
**S19**	546	14.45	−8.54	−1.46
**S20**	162	15.21	−8.18	−1.12

TPSS/def2-TZVP-calculated geometries and energies of the molecules

Substrate of 5-exo-cyclization of 5-hexenyl-radical

energy: -235.3181814961

-2.69667685862476	0.60610438555252	2.84518747439439	c
-3.30534118047394	-0.89992574206802	4.77189365214970	c
-0.11426768497830	0.81467902153926	1.69142048167380	c
-0.07133568331474	-0.01143294946359	-1.09869587175043	c
2.55679014567369	0.29916898541854	-2.31405492079484	c
2.65832444193266	-0.59390770420869	-4.98202518986430	c
-5.21809128840526	-0.99071305211985	5.51162901199612	h
-1.90181937317800	-2.09562286407393	5.68306138619310	h
-4.16413427926659	1.77077772068192	1.98219734971786	h
1.23321611916069	-0.32741800510023	2.77797175089586	h
-0.66324993496593	-1.99457304861037	-1.23100380217427	h
0.53533830125812	2.78688175667326	1.80263596973657	h
-1.45624307781801	1.09395049820401	-2.17588985206674	h
3.92736449722146	-0.76288659245216	-1.14559307667997	h
3.14511997386356	2.28518105498928	-2.20064499991075	h
3.74824127973836	0.39766203706559	-6.40884546892429	h
1.78676460217704	-2.37792550202755	-5.50924389459183	h

Product of 5-exo-cyclization of the 5-hexenyl-radical

energy: -235.3430983267

3.53315569418480	-0.04240195120103	2.47322800337540	c
1.48103375284337	0.67824356556090	0.70395500343715	c
-1.20950196769005	0.57223795985316	1.86226395599556	c
-3.04448412287719	0.44910845874008	-0.41729708471005	c

-1.39880171096001	-0.31410876153486	-2.73146673834674	c
1.17095408373798	-1.09249462179618	-1.61948040326864	c
3.39652589793134	-1.79563107760412	3.53823143436657	h
5.25304606276380	1.05905456032637	2.65778800862806	h
-1.57827375071574	2.17364151196450	3.11637946529411	h
-1.36055872130485	-1.15041347254806	3.00606362242089	h
-3.95659448733795	2.27703324467996	-0.73938196267250	h
-4.55435563798503	-0.92200495536470	-0.07537391160130	h
1.11896368337039	-3.05696100509603	-0.95473832906086	h
-1.14433089417237	1.31141834307217	-3.98911240304614	h
-2.26062762995464	-1.82196488080237	-3.85437095257574	h
1.81941632092409	2.59715308866865	-0.01480085801127	h
2.73443342724205	-0.92191000691841	-2.96188685022449	h

Transition state of 5-exo-cyclization of the 5-hexenyl-radical

energy: -235.3105501906

1.93015922400954	0.03000151745422	1.84787888556499	c
-0.67831064434014	0.61363700295227	2.99075069294752	c
-2.43577944574538	1.39163967749115	0.91698247494766	c
-0.49653670753482	-1.02405712053255	-2.12094992858275	c
1.54685364631101	-1.89170460987108	-0.29669509406695	c
0.02470455436297	0.49391303667589	-4.14441945403266	c
1.00911113729649	-3.71878874714360	0.52137275378924	h
-1.40420416671255	0.94218663215297	-5.54702663187541	h
1.84219802136171	1.43562420318269	-4.33416513428016	h
-2.22596510864207	-2.13331903291774	-2.17667814165144	h

3.32306189486275	-2.18196100265589	-1.32129839529511	h
2.73506039775196	1.77356602070386	1.06284819288898	h
3.25299586951484	-0.67915430157486	3.27732157373602	h
-0.49555780467722	2.10410140766464	4.43254428469045	h
-1.39924150720792	-1.07535773527245	3.95275443455586	h
-4.36212025886154	0.68065210460710	0.87657147171588	h
-2.16642910174987	3.23902094708296	0.06220801494763	h

1

energy: -596.9645033308

2.14844952582951	-0.67411782778304	-4.17932864087178	c
1.40870862058972	-1.12247343604958	-1.67134366358551	c
3.04839674491894	-2.46695330818332	-0.07603811297683	c
5.37162985757584	-3.31633363497773	-0.96932911513192	c
6.09534702135777	-2.87801118477370	-3.46958891773714	c
4.46294749027188	-1.56239632949210	-5.06730316804723	c
-0.97841579207162	-0.28528716999335	-0.80931850655438	n
-3.18645138811834	-0.74395219613261	-2.41839605263154	c
-4.57788744571857	1.60247233510547	-3.10804956483185	c
-1.34040284503198	0.72204894864433	1.61585512050832	c
2.47915280251387	-2.84833274518950	1.85699730558571	h
6.61052248644177	-4.36148150174933	0.29142835174856	h
7.90489610175625	-3.55389111240361	-4.16095664021172	h
5.00249293573534	-1.19208342252567	-7.01349095589416	h
0.91801026155919	0.40206346007620	-5.42120700091567	h
-4.46628792298102	-2.10875992887899	-1.49388060512228	h

-2.48376856998281	-1.73238636444531	-4.10487693608280	h
-6.48149493223005	1.49349435557715	-3.86595624198145	h
-3.66739098161898	3.43099880924737	-2.94642453880114	h
-3.74851367262087	0.73508028852461	2.74618644260035	c
-4.10185130481416	1.79505425298824	5.13198515464795	c
-2.09167034135639	2.86702090549601	6.45177354192271	c
0.29585692920010	2.88850334953575	5.32382791045118	c
0.67472132168983	1.85007376651766	2.94158613106003	c
-5.35689714546236	-0.07177462158104	1.76849942268645	h
-5.98124625816693	1.77100222410919	5.95946739168800	h
-2.37772176667742	3.68653563954033	8.31034973711782	h
1.88605483169389	3.74915477041179	6.29773055959386	h
2.53281343571761	1.92473167838502	2.07980159176639	h

Entry 4

energy: -596.9847575536

1.88747453276295	2.24284235410933	3.14297225208270	c
1.35732114751081	2.63612268872000	5.89263302426692	c
-0.99629764627669	2.17570436376424	6.85254346470565	c
-2.95208892869504	1.11060629593438	5.35628669527653	c
-2.47031598141035	0.30300567285541	2.85877427709440	c
-0.13064906857645	0.73983142425558	1.80660711321025	c
0.91077951212966	-0.19895167892387	-0.41215455474757	n
3.70540003148143	-0.05997616434155	-0.33861947620130	c
4.30428790845571	0.79136870879128	2.37061668542778	c
-0.36059375939000	-0.97768242639940	-2.59345788481934	c

-2.88210727441581	-0.26114806481304	-3.05730941472642	c
-4.10075108191606	-1.03023473240172	-5.25396074804678	c
-2.85955004877438	-2.51377118209360	-7.04958872149452	c
-0.34884898298037	-3.19092341220115	-6.62008747466066	c
0.89821437449203	-2.43594915049454	-4.43004508135440	c
2.00281187778357	4.11559570993383	2.19700047605044	h
2.84623506643620	3.42451436551279	7.06796916461723	h
-1.37981665707301	2.58869711813942	8.82865961410077	h
-4.79405366588459	0.76092201826232	6.18661385998621	h
-3.89107038463801	-0.78354150749965	1.85866483878370	h
-3.85161050421909	0.95143157690804	-1.72324933691608	h
-6.04172939654659	-0.44022905152674	-5.57275878170083	h
-3.82516522810215	-3.11265862029536	-8.75736572606131	h
0.66255738226242	-4.32491770155884	-8.00120095977060	h
2.84953042975807	-2.99223688740496	-4.14674765515812	h
4.53253542681777	-1.90590398458267	-0.76568586725274	h
4.37036898067352	1.29397642503217	-1.76387534636775	h
4.54192389172663	-0.85089640117531	3.60523838362301	h
6.01520804660769	1.94440224349367	2.46152718005300	h

Entry TS-1-4

energy: -596.9514551991

0.65028440308462	-2.23555895780136	-4.56198189175604	c
-0.44570732728114	-0.88444519730979	-2.54444457778705	c
-3.01040251300684	-0.18541848143457	-2.75200372097643	c
-4.42049839491595	-0.88079885330037	-4.85478718334356	c

-3.33752934407214	-2.26415746063094	-6.82579980739738	c
-0.79066913038459	-2.91295880111239	-6.65892581217535	c
0.98820855384459	-0.18797426561156	-0.44993817095378	n
0.04400572870931	0.84189054810657	1.80102674355319	c
1.67697204288050	2.66722976476283	2.94624286222107	c
4.76351965937960	0.09339403294367	2.16027068505246	c
3.79150153450663	-0.42687443574887	-0.45515133137380	c
-2.02108563462640	-0.11113890646975	3.12773105823043	c
-2.49773145182357	0.72262879829565	5.58985822758901	c
-0.88569790766081	2.47290482261104	6.76262130749792	c
1.14910155028312	3.47335340475430	5.44705307802656	c
2.73332723925622	3.93145631776449	1.71867773865368	h
2.33098290898589	4.91509007584280	6.30702558404042	h
-1.27329523892819	3.09124364584208	8.68112631476810	h
-4.10340201684788	-0.03953144184887	6.61619370751400	h
-3.19153222590955	-1.56910737074045	2.28518154830472	h
-3.87404963043272	0.93612586955522	-1.27100110824426	h
-6.39113636750792	-0.31192937201065	-4.96004060611006	h
-4.44998790912984	-2.79973345612678	-8.46370476930403	h
0.10673411408984	-3.95951084558300	-8.18113102696848	h
2.63123305997604	-2.75296857120440	-4.50934957752558	h
4.26643652616423	-2.35697105067390	-1.05321996789136	h
4.61586456522797	0.87698873829663	-1.84527536729051	h
4.46834628101100	-1.34841875711018	3.59125294939552	h
6.47620692512761	1.20519020594264	2.35249311425007	h

2

energy: -593.7931518979

2.66404695046249	-1.24905782257828	-4.77702014041604	c
0.44645039389826	-0.84750299402998	-3.41367696716521	c
0.55513739388726	-0.01466062997479	-0.90526463186669	c
2.91079620523932	0.44429512583828	0.20632088342227	c
5.11795753054941	0.01770819003450	-1.16114158050108	c
5.01108443282348	-0.82927479898783	-3.65708648398213	c
-1.74504224351354	0.47619550811864	0.41908128786255	n
-3.84584572274369	1.68440172860431	-0.96731666552141	c
-3.00898641833552	3.95970932718805	-2.38050529762596	c
3.00355399473050	1.12657635510000	2.13430898660525	h
6.93400593429332	0.37465075674817	-0.27278170704324	h
6.73813062246295	-1.14675278687665	-4.71867000541582	h
2.54825044456286	-1.90067131780885	-6.72025070133900	h
-1.37114148567093	-1.19077496974920	-4.29798231149933	h
-5.25338350887650	2.13526005384365	0.49130012309824	h
-4.75248118831308	0.31156121237744	-2.23897842338801	h
-4.05307791217023	4.59061916711498	-4.02730961079742	h
-1.50321416221668	5.14132639505719	-1.64648903456925	h
-2.27191626241531	-0.34800916687168	2.83461396547709	c
-4.25578120579328	0.05521608441003	3.94779151306241	o
-0.33055927348564	-1.69776336556940	3.84212204966031	o
-0.81612576591280	-2.60200209662147	6.37352784784026	c
0.88622549355392	-3.63683536133238	6.89693967902848	h
-1.14041017374670	-1.01399911814556	7.65259623354679	h

-2.46767407327004	-3.84021547588927	6.38587099152668	h

5

energy: -593.8125945264

-4.06739209804298	2.50309186618888	-2.71766739992365	c
-1.98548170219103	0.59045216181886	-2.69710307427286	c
-0.87020185183438	0.18202153244301	-0.10494271503717	c
-2.09290182101164	0.90115649606581	2.06658796007093	c
-4.34836420831542	2.32240470701928	1.86863817034007	c
-5.21915963286161	3.19769083307547	-0.51240322818829	c
1.53085828044956	-0.87840396782903	-0.45940147674758	n
2.28570193927304	-0.81297292530219	-3.16166807450518	c
0.39299281326038	1.04069840484029	-4.33090442371226	c
-1.29561191291022	0.48777046217687	3.90332051013272	h
-5.32654616601907	2.88779296635750	3.57987080713493	h
-6.82042357129780	4.48406570608675	-0.56799985568410	h
-4.73714518306904	3.19366894252399	-4.53238159535140	h
-2.77519268444306	-1.25352588418020	-3.33167992327045	h
2.11143432395552	-2.71213281671307	-3.97164123620836	h
4.25731921232912	-0.23030200649747	-3.30266909972694	h
1.04365152047567	2.99056187497879	-4.09302459141148	h
0.08265544962772	0.67810735831401	-6.33995395643990	h
3.19645124729489	-1.93813376642208	1.23404286654584	c
5.24923976152822	-2.80163908916402	0.62430770877042	o
2.28631644085876	-1.91409977549225	3.63528235293358	o
3.98548246059260	-3.03331961719309	5.46227108833638	c

3.00298642199313	-2.87207088533361	7.26507082860758	h
4.33991571416446	-5.01044060676246	4.98657559223571	h
5.77341524619316	-2.00244197100013	5.49747276537143	h

TS-2-5

energy: -593.7800845656

3.86787118030262	-0.80801433159536	-3.44467306358098	c
1.39177506514159	-0.33256150264034	-2.63612144734233	c
0.83010303403124	-0.41280323115228	-0.07480913512656	c
2.75627961199998	-0.96574588145003	1.74665991024832	c
5.24453018175481	-1.44271053115975	0.86398431850192	c
5.79492879603594	-1.30475665827342	-1.69479379501768	c
-1.40972912802288	0.50343827531733	1.05045776805709	n
-0.96396551951504	1.83749114858506	3.49755690892132	c
1.79183043673256	2.49521025988402	3.62601567310433	c
-2.21726518715510	3.48296348765340	3.52356505011346	h
-1.51897491186283	0.61200994710165	5.07416703933580	h
2.71394004105449	2.43099609550528	5.45758082445450	h
2.49520793819176	3.91814715939854	2.32279731254488	h
6.70417412140949	-1.95262408731693	2.21416607545231	h
2.19243243099627	-1.81441268591972	3.53233492096519	h
7.70781868290611	-1.65252287525613	-2.35331724758576	h
4.29551127738059	-0.74415651591735	-5.45071382533281	h
-0.06623380758825	0.15224558465538	-3.98779405125150	h
-3.84629212432881	0.38332003379108	0.17329601610210	c
-5.65080231127946	1.32326923544298	1.26529320308066	o

-4.01949469000216	-0.93206486001700	-2.03237752589779	o
-6.57279400059764	-1.14471497863781	-2.98255732015315	c
-6.39727915332948	-2.22205651318367	-4.72927966254209	h
-7.76180192324796	-2.13864321293302	-1.61850893681825	h
-7.36177004100780	0.72869663811786	-3.34292901023297	h

3

energy: -405.1058130788

0.19696664634266	-0.47398190206514	0.62385653120577	c
0.25350881483807	0.11153677415161	3.22799411938065	c
2.52450253787919	0.14568341684567	4.55467875474626	c
4.81317260124929	-0.36944369521205	3.35351338664113	c
4.78232557192398	-0.90904571816207	0.77278575443690	c
2.53058065165261	-0.95221260348307	-0.58412915395944	c
-1.48446140152693	0.53332654864808	4.22637267408314	h
2.49309120218015	0.59441260634570	6.55890883043379	h
6.57864743776368	-0.33205570033979	4.39690248867988	h
6.54058198949225	-1.28953242603978	-0.21866641874862	h
2.59868820552450	-1.33947162783728	-2.59411077462697	h
-2.08351940540965	-0.60450365542616	-0.67652330490039	n
-4.35510052868827	0.40620768233528	0.50533852442672	c
-2.07978396268837	-0.72743300057334	-3.43731947268792	c
-1.43188477370070	1.71569738106894	-4.69551790945932	c
-5.92748201383702	0.18555767599404	-0.81180470890205	h
-4.80693523366402	-0.64688569008207	2.22998497536355	h
-4.17914738065601	2.42064979445457	0.99379817315660	h

-3.96213965151973	-1.36504846841055	-4.03048762157228	h
-0.78598850655930	-2.25537673086683	-4.01619111817160	h
-1.98271162375447	2.07474361029574	-6.63991761673291	h
-0.23291117684189	3.07717572835854	-3.73946611279283	h

6

energy: -405.1239931752

1.98395187297715	0.78857837057922	-0.77412569460061	c
-0.42322212026298	0.45015859225104	0.71448012047365	c
-0.45620597430962	0.16476923783492	3.29856360070587	c
1.86850905552544	-0.11404790682469	4.59725259979090	c
4.16563040789055	-0.33222736275321	3.23524698451439	c
4.27461723565802	-0.04902078668664	0.66212176058149	c
-2.39825576559118	0.44269722355292	-0.96050423793300	n
-1.53578600982052	0.06650495719261	-3.58013910326591	c
1.30045500014840	-0.53018071745299	-3.29722426671265	c
-4.99786629043257	0.00819130972252	-0.23847568867714	c
-2.23023132172794	0.01674576765460	4.32220754464229	h
1.86745378921814	-0.33920197649966	6.63553957573483	h
5.88388140488887	-0.78081064202766	4.27002100835912	h
6.05143707131146	-0.23738391527951	-0.35139512905319	h
2.16536122981866	2.83603266381550	-1.21248285735320	h
-6.24417484865032	0.61501272238226	-1.77057346429268	h
-5.37274665498612	-1.99809946112917	0.17554921773721	h
-5.44883989806000	1.11894496605705	1.44608441140953	h
-1.86289076554215	1.78416022222258	-4.70352384283068	h

-2.58853438160747	-1.48374324852511	-4.46640498067093	h
1.58233938757016	-2.57097105968258	-3.10189338440243	h
2.41511757598414	0.14389104359597	-4.90032417415680	h

TS-3-6

energy: -405.0903963673

-1.59541116965038	0.15897111159114	-3.62126121145507	c
-2.29710716132972	0.84827121628191	-0.98025652805910	n
-0.26655667992804	0.70114221205697	0.65464909616049	c
2.14950455593600	1.15904815114521	-0.50992081238927	c
0.88889260003806	-1.22376534421441	-3.60236494393210	c
4.36380108072161	0.67843935736538	0.91458835538263	c
4.21118752755562	-0.24479331908238	3.36869932358844	c
1.84288250062625	-0.56994500970216	4.50821248770022	c
-0.38974602539903	-0.11132473322878	3.16040737082634	c
-4.82138885537539	0.21472254566805	-0.11609075131278	c
-5.28545242262296	1.32451568386283	1.56492241324953	h
-6.17853468358876	0.69707675161813	-1.59579711126658	h
-5.02983485444473	-1.80790919805450	0.34919456524089	h
-3.13744719687549	-1.00353938218825	-4.38599451169552	h
-1.47084494448441	1.86386011183797	-4.80037869449309	h
2.19584883243888	-0.91730469238027	-5.15561022719422	h
0.92743015869045	-3.09819284685403	-2.76253361132185	h
6.19394082413137	1.08990348778973	0.07892236858079	h
2.25804758527582	2.57379132664340	-1.99643323046271	h
5.92301025308169	-0.62525333276500	4.43519002492104	h

1.72219948168017	-1.22626462228213	6.44971699426881	h
-2.20442140647723	-0.48144947510842	4.04213863366407	h

7

energy: -828.1796954096

2.64683930816430	1.12480521362671	4.71008054223602	c
0.64417804672280	-0.53556899915874	4.18570230646278	c
-1.08230148964287	-1.09849177541824	6.11942272159578	c
-0.81051063526816	-0.02503411892984	8.50700150253818	c
1.19559798267084	1.60627833641572	9.02491420697539	c
2.92279561437000	2.16508620917851	7.11034717169549	c
0.32870642150116	-1.59714548432111	1.74708049173790	n
-2.16152196309838	-1.29879014926725	0.53290063821760	c
-2.51697339578257	1.28356036878854	-0.56959539524607	c
2.42387331129521	-2.18808917220927	0.22210039476401	c
2.33225818374215	-1.96407074060048	-2.42313018632454	c
4.39341410279488	-2.69479290014708	-3.89541294631986	c
6.58716509218139	-3.63408043611102	-2.78451422965004	c
6.68628774539013	-3.85614307160555	-0.15377883277111	c
4.63837689750936	-3.16001260266445	1.33424034897418	c
4.72444927337547	-3.38284818731443	3.37108746724922	h
8.36843712403432	-4.60887754483113	0.75257259678218	h
8.18957536219941	-4.18808604505868	-3.93948510497889	h
4.27464530139163	-2.49561015036557	-5.93592032198412	h
0.67670574904536	-1.18119047441514	-3.34092416299319	h
-3.59779908027705	-1.67243340588108	1.97961987020700	h

-2.34927022368862	-2.78462493950132	-0.89017125549991	h
-3.53871133450283	1.84993350854693	-2.96874128175303	c
-1.89843315802716	2.85035576435257	0.61104994591695	h
-2.62264179167208	-2.40635740693207	5.76092675263717	h
-2.16348544455904	-0.49287270450197	9.97892761643189	h
1.40976190261684	2.42954267425432	10.89190444519051	h
4.48380772640604	3.44852924432387	7.47500148644223	h
3.97949713888489	1.58766867699604	3.22141107103467	h
-3.70483606925226	4.41641631694050	-3.77444279678324	c
-4.67283127526058	5.04828842507622	-6.12020264057965	c
-5.51920272043020	3.15962595739574	-7.77438517926552	c
-5.38050918313255	0.62382274674365	-7.03037647782118	c
-4.41857716162440	-0.03196353618893	-4.68364263258565	c
-3.04361246659960	5.88611396748954	-2.50000649457502	h
-4.77303601160128	7.02056541396706	-6.68281127750261	h
-6.27590879278955	3.65766427189711	-9.61541042097062	h
-6.03569542785590	-0.84924238281809	-8.30232890067555	h
-4.34051465923133	-2.01193086775168	-4.15101103880944	h

11

energy: -828.1781445740

-1.27041884001449	-2.09068897488034	-5.74854623135595	c
-2.40858877625278	-0.58074612270032	-3.87584665288011	c
-5.04323571283725	-0.21304441477278	-3.97195069186669	c
-6.47844337978155	-1.36569074007998	-5.84630710239794	c
-5.34824957294896	-2.89698504192602	-7.67503977162322	c

-2.73451613964135	-3.23100884472721	-7.61348272537377	c
-0.92978024304239	0.58407155793182	-2.02221194264382	n
-1.72467616976923	1.60371584890008	0.26175104843222	c
0.26304134829800	3.44003486787116	1.12517912363255	c
2.74702416025377	2.18765883913167	0.08545386687751	c
1.80288313005617	1.03193183179559	-2.41654713494217	c
-3.77579783772669	0.96689675036604	1.73302870702087	c
-3.95947896437476	1.93576413243307	4.20998759795009	c
-1.96990086423865	3.40099938245855	5.25715453088648	c
0.10497184373627	4.06627377107116	3.87426094305139	c
0.03827222728848	5.19888138509488	0.00273758131751	h
1.63538098624401	5.14522991719009	4.71616149469274	h
-2.09077253689554	3.96506254721129	7.22902369274844	h
-5.57168124026628	1.42777840237585	5.37047472409291	h
-5.17244208451466	-0.37675813640028	1.06641118658232	h
-5.94130765770621	1.02748276319987	-2.61335073535827	h
-8.50587453544581	-1.04198371249587	-5.88731210070025	h
-6.48155679022964	-3.79592105659103	-9.12934370208418	h
-1.81168787791860	-4.39630677677480	-9.03001842370902	h
0.75677356626604	-2.38621002431675	-5.74528826141377	h
2.77640604154014	-0.74528625277553	-2.82418176631155	h
2.07699988370029	2.31645259009801	-4.02222971492364	h
3.83323358627795	0.27702583723265	1.90600900822957	c
4.17170051366283	3.63660137715547	-0.29197609146603	h
5.96111357816991	0.90376343635060	3.34558280728740	c
6.95060936866741	-0.78261735381638	5.11208919996222	c

5.81495924087605	-3.13401148996194	5.46891673611520	c
3.69304554655957	-3.78152903995923	4.04382059532001	c
2.70995889890381	-2.09269816439835	2.27746412240990	c
6.85183527206861	2.73705489808133	3.07487920238695	h
8.60879288655207	-0.26128420499901	6.20514188351798	h
6.57946817427932	-4.45375394601233	6.84284279430199	h
2.79423463014166	-5.60823014127061	4.31057922429049	h
1.04770434006228	-2.61792569709051	1.19468297794381	h

TS-7-11

energy: -828.1608467117

3.15610659861675	-2.13773066371227	1.45195620229689	c
3.96011842365674	0.39947197472918	1.59885349505630	c
5.65252336252524	1.06213873437208	3.54834318227141	c
6.51956584007571	-0.72981988426698	5.25970737984247	c
5.71012021480454	-3.23891284387476	5.08216271501481	c
4.02773388399755	-3.92753976302636	3.16989525703488	c
2.98290744156834	2.33640473209340	-0.08629707476540	c
1.72289769426887	1.57875581555719	-2.54223255890266	c
-0.95658408921436	0.90573482648071	-2.01680573250915	n
-2.46276368315028	-0.28015970839266	-3.82417527943362	c
-1.39239408339540	-1.34029142529452	-6.01971759168451	c
-2.91778812087992	-2.51354144994519	-7.81642775969530	c
-5.52852209511435	-2.65467529706454	-7.49833528108633	c
-6.60405755833018	-1.56034252406008	-5.34946766474051	c
-5.11094222579058	-0.37971121827859	-3.54124395701524	c

-1.68461207155593	1.55982380611829	0.43659576803514	c
-0.29415318964773	3.70383552448769	1.38156536996008	c
-0.43513015275107	4.23541479682096	4.03016340573042	c
-1.81537565278587	2.69092425302320	5.61638804640713	c
-3.19754191589769	0.63853067322689	4.63473350459658	c
-3.10836123909054	0.05867437886736	2.05565267270264	c
-0.10671941331522	5.31611847855168	0.11263192697897	h
0.54283490099418	5.88073816378246	4.77239353179181	h
-1.89083835202976	3.09246788906174	7.62805686845679	h
-4.28668044212856	-0.55802730954961	5.89629950155198	h
-4.04244666274492	-1.62261565518147	1.34489178692674	h
-5.98384074750354	0.50999672856159	-1.91527565185573	h
-8.63986456873196	-1.60406133895275	-5.08295993518685	h
-6.70559984156161	-3.57330965225077	-8.90465815336023	h
-2.03917528654627	-3.32231252568624	-9.48698013193832	h
0.62856439869425	-1.24877773251383	-6.33862517342458	h
2.66937159119576	-0.07305496509760	-3.35989976524546	h
1.81166616880841	3.11610158326262	-3.93209036156357	h
4.10022900390604	4.05910469156518	-0.17159913222194	h
6.28006686879968	3.01367184838048	3.69985052725853	h
7.83027414700270	-0.17583168208690	6.74026391761828	h
6.38719387923497	-4.64104943357484	6.41938945737869	h
3.38663769334106	-5.87262634285922	3.01949642970750	h
1.83457928067536	-2.70351748327650	-0.01249974198730	h

8

energy: -636.3081061007

1.36615633152472	1.45274077056979	3.50009855458659	c
-0.64656775923964	0.54640850529379	2.01090661855730	c
-3.11213686478845	0.71961819743440	2.99568153087304	c
-3.52743333123729	1.71843081653565	5.39762076499868	c
-1.52364362931807	2.57241022645555	6.87711684824617	c
0.92398640091828	2.43375658697435	5.89591039574107	c
-0.21967312678483	-0.39801396253132	-0.42779369201681	n
-2.35741646920909	-0.58810496664952	-2.18074038441451	c
-3.51707759745189	1.90438438930606	-2.80664788723775	c
2.13815144783642	-1.39960646446428	-1.18432015263428	c
3.06528172166108	-0.92336733632976	-3.62421019805597	c
5.35000308698499	-1.96874625346328	-4.41253324955169	c
6.76929769389885	-3.47409875059674	-2.77912299649633	c
5.86022704161951	-3.94113731718917	-0.34544738711084	c
3.56331260321135	-2.93389072947863	0.44583130630110	c
2.84735734241455	-3.33727834442431	2.32488712711919	h
6.93031471807391	-5.13178747090530	0.94067743261319	h
8.55687900319291	-4.27333163431696	-3.39166401713275	h
6.03644960800176	-1.57300005185213	-6.30684511009921	h
2.00589203923157	0.29758939292042	-4.88954705699536	h
-3.80428640163933	-1.87714237435723	-1.40308613226021	h
-1.63563804545563	-1.55111285332543	-3.87590683247649	h
-6.10179841152082	2.07125595527981	-3.90768144139801	c
-2.31900544434802	3.56927064892847	-2.70641360445952	h

-4.71451288005589	0.07687952055208	1.89449485515736	h
-5.45066765827760	1.82108107653395	6.11066534424216	h
-1.85854460377193	3.34712344567064	8.74677345140144	h
2.51437344691798	3.12334170154053	6.99736113101782	h
3.27466786641314	1.40261403666407	2.75429398886627	h
-6.89000340775588	3.97313683793186	-3.71174141640868	h
-6.12576863476791	1.61030399074099	-5.94360774002864	h
-7.39817608627876	0.73027241055167	-2.99501005094423	h

12

energy: -636.3242690509

0.41319551783498	-2.23692814046075	-4.85352716506222	c
-0.87307477531945	-0.84359187124851	-2.98625146949556	c
-3.46042408575307	-0.33252239794636	-3.34723927586546	c
-4.71160540525700	-1.23677286610090	-5.47316312075347	c
-3.43876792346912	-2.65546447858656	-7.29860780753516	c
-0.86640200482059	-3.12877609232395	-6.97207562929420	c
0.42399482880152	0.07310118990059	-0.87407892436152	n
-0.59353443386052	0.94150748916521	1.38233615498122	c
1.36550002587881	2.59206255049366	2.62463306410282	c
3.85833044372998	1.26385078474789	1.79591115881454	c
3.19483666993124	0.46700448007058	-0.92233652967304	c
-2.84031392261830	0.31940702184524	2.53953806055792	c
-3.28366096975085	1.11478101206513	5.04747180088290	c
-1.37715019676927	2.38950800591018	6.43880752208779	c
0.88809920738952	3.03102963892195	5.37565262897181	c

1.35470359424788	4.44120203775437	1.62869732436919	h
2.33302823150155	3.99643798914112	6.47154582494704	h
-1.72717832010801	2.83300114965387	8.41456525610486	h
-5.05240505674743	0.62079590920699	5.95971537423945	h
-4.20830028088690	-0.88830991396926	1.60694283902485	h
-4.46110111805697	0.82812617542572	-1.98995186618547	h
-6.70473575097735	-0.80431080662601	-5.71313911113902	h
-4.42865649926607	-3.36020755317311	-8.95119808118224	h
0.16843347177158	-4.20901736785141	-8.37870273044697	h
2.41216161935307	-2.63638994630428	-4.65001299566075	h
4.16283072324705	-1.28218229953345	-1.45188873347111	h
3.66908490008416	1.92547231355912	-2.32098612427357	h
4.47347610489232	-1.01970081219342	3.46020742017646	c
5.44423928420831	2.59217999644861	1.78811719180344	h
6.16610666643300	-1.99308788974407	2.77211374996738	h
4.80024857268395	-0.43791748049319	5.41732155431713	h
2.89904088167204	-2.36428782775502	3.45958263905107	h

TS-8-12

energy: -636.2945833590

-3.88300805698456	-0.88075794232744	-2.74724360514321	c
-1.24962444478284	-1.29198446176038	-2.68064429420303	c
-0.13861526358746	-2.61270660076459	-4.70972144395770	c
-1.62257151341744	-3.53934792849267	-6.67725791177931	c
-4.23052472281936	-3.17346615554986	-6.70265195612052	c
-5.33502820811173	-1.82023734580200	-4.72282865490655	c

0.21983988386725	-0.34832756090812	-0.70973084716184	n
3.01490839653424	-0.21990166130521	-0.89657351858764	c
4.08109933079005	0.43019783861929	1.65993237675624	c
6.41163060612553	2.02260387348107	1.81843556208141	c
-0.69909708245928	0.65721637240250	1.56535121689423	c
0.82056996525514	2.66834117246032	2.55110235970284	c
0.37031116033874	3.49997843605674	5.06160623033459	c
-1.46233959533965	2.34124511422967	6.53594013213926	c
-2.96279928887405	0.40411563852273	5.51972602260616	c
-2.56349565085376	-0.45596227643995	3.05004761492298	c
1.64351086753158	4.00530613815491	1.22136512234806	h
1.45069485821471	5.08051383331072	5.80393350648073	h
-1.78531127158822	2.97792679605899	8.46063305914711	h
-4.41464199693477	-0.47961707837122	6.66999529185568	h
-3.62978020380200	-2.05192869663250	2.32743404842803	h
-4.77037286641708	0.21214506722335	-1.25892127580951	h
-7.35845966260762	-1.46814962603903	-4.72157350891532	h
-5.37588453202167	-3.90214107712667	-8.24032633753669	h
-0.71111766519402	-4.55707279082926	-8.21066620661262	h
1.88640559063175	-2.91445057200409	-4.76735494499337	h
3.71350325145407	-2.06550077811254	-1.54178307247685	h
3.58393247379401	1.18990743779681	-2.31686215982428	h
3.87450596130273	-1.03759764447579	3.08747145353638	h
6.75766365864785	2.64335921118946	3.76214475192017	h
8.10745706180860	0.98440662651705	1.19744619377173	h
6.25663895949923	3.70188664091778	0.61157479510303	h

9

energy: -824.9891101855

1.69680329004086	1.15413222779271	3.53268913236272	c
-0.14986141278125	-0.16627369478041	2.15326237141014	c
-2.65837176661430	-0.12593283431288	3.02261959114698	c
-3.27215992114811	1.15763988707814	5.23704485552939	c
-1.42504176641056	2.41619456080912	6.63340205165081	c
1.06218287964866	2.40710294714340	5.75473897665902	c
0.49167389224097	-1.44579044100879	-0.10419438961693	n
-1.41521130247601	-1.66076770060820	-2.12381373337407	c
-2.03362531053318	0.86336755414722	-3.19357556560739	c
2.96042294749668	-2.32364489291279	-0.57898463233719	c
4.00803200936229	-2.21425191561220	-3.01602650722861	c
6.40882301094882	-3.19369986928042	-3.48446487732417	c
7.82200210526277	-4.27255012776349	-1.54241112403913	c
6.79051885576245	-4.37812719672484	0.88723950479213	c
4.39105222928263	-3.42800914978586	1.37224523236705	c
3.58776601271688	-3.55905312034022	3.25383082384024	h
7.85201626378678	-5.24158749475867	2.41810057194485	h
9.69497687166248	-5.02377873968902	-1.91074954924149	h
7.18173930588714	-3.08158011952986	-5.38318086922307	h
2.96672540517135	-1.32748100745860	-4.54462352378401	h
-3.13278916861352	-2.54672328748029	-1.39097337163405	h
-0.62838504906424	-2.93798063477765	-3.54941361937644	h
-4.57223583015408	1.84463181142341	-3.05887991497345	c
-0.53801639300748	2.06137759723885	-3.92645674443515	h

-4.14790117703614	-1.05094080806579	1.96405338909592	h
-5.22504167870138	1.16120710928087	5.87044497747429	h
-1.91706717856406	3.40566708846485	8.36188826097396	h
2.52480073705600	3.41348526958120	6.78649100732504	h
3.62722458341556	1.20617950932784	2.84499234512692	h
-4.74730248124274	4.20796807873860	-4.09488385648685	o
-6.39622960549542	0.72768389220593	-2.14954848199390	o
-7.25549888056200	5.28130112509318	-4.01755277538466	c
-7.07647695298836	7.13594722032644	-4.89463505309397	h
-8.57649619251238	4.08906389770485	-5.06744647889656	h
-7.89904833183697	5.44522325853345	-2.06122802364839	h

13

energy: -824.9944266111

-1.20289363223410	-3.31130234894595	-4.91202640798695	c
-2.32117156113501	-2.09006836418130	-2.82970617219236	c
-4.97668894337030	-1.97021661411675	-2.70479083622901	c
-6.44618632963074	-3.08935246813399	-4.57289889154530	c
-5.33067227176159	-4.33923517619267	-6.61215172304323	c
-2.70116117443510	-4.42133082127186	-6.76808581468147	c
-0.81143578967253	-0.95176215924729	-0.98231668691471	n
-1.51243081588812	-0.20755543667025	1.43016078535329	c
0.34976922840061	1.75142022539950	2.30894909468823	c
2.83433589949818	0.80548311832859	0.99702404534137	c
1.82889514768705	-0.21354313000840	-1.52952236139581	c
-3.38300069528300	-1.14018339147144	2.98317424425142	c

-3.45366903005424	-0.39159928105715	5.54063146229236	c
-1.51995993457146	1.15811304908598	6.56927642556528	c
0.37364680619419	2.12868692160756	5.10542212984489	c
-0.10477644565487	3.55834485984138	1.34830654807985	h
1.85691692855750	3.29208812150290	5.91912584457921	h
-1.53477763480717	1.54764573575442	8.58647520403061	h
-4.92071552398906	-1.13620929936114	6.76414250967093	h
-4.70703946513348	-2.54861389081766	2.30381809847501	h
-5.87354074150768	-0.94128672747442	-1.17911584423296	h
-8.49121519233372	-2.95902521694061	-4.44109778637609	h
-6.48977265099484	-5.21224183203632	-8.06185279365713	h
-1.79194212652432	-5.36033893351928	-8.35148578872883	h
0.83627688231089	-3.40198227351776	-5.08578675571603	h
2.92337687237702	-1.84919430266718	-2.16138079246155	h
1.88053300792090	1.24475174627675	-2.99919878970269	h
3.65301181598498	-0.70628430214845	2.13909274814893	h
4.71821418427410	2.91564295595594	0.66893424766654	c
6.63677574020505	2.71780048140364	2.36000805242493	o
4.52411082141852	4.64087958367888	-0.83596618908380	o
8.45787015351518	4.76361174686581	2.25726002707845	c
9.84394469537628	4.32052960809547	3.71323359293675	h
9.33470065261413	4.83412469918405	0.38980735950746	h
7.52067112264670	6.56220311679878	2.64254121401239	h

TS-9-13

energy: -824.9757886899

-4.86076285724883	-2.06076728471393	-2.71250222582897	c
-2.20390113734918	-2.22521329508674	-2.81435463088673	c
-1.10480415640457	-3.68149081320801	-4.75147381677073	c
-2.62160635182322	-4.98328046677376	-6.46426307345698	c
-5.24860055945728	-4.86540676930266	-6.31401617526105	c
-6.34703716365450	-3.37754217157397	-4.43015159270344	c
-0.70625416964538	-0.90587012547435	-1.08813721452387	n
2.03490197984998	-0.46035122483162	-1.55411546303607	c
3.21577760522074	0.51193535810859	0.84630731355442	c
5.04116642286473	2.56001759046115	0.66439109883987	c
5.21160029522440	4.07044976798551	-1.08482376804181	o
-1.52097465036292	0.12017497471288	1.19194282194118	c
-0.10201537876794	2.31828867341177	1.94233871036350	c
-0.36750541024166	3.20983002834280	4.47244307736496	c
-1.95576039926676	1.98593414186682	6.14287686807492	c
-3.39823777665378	-0.10401776830065	5.33695890036265	c
-3.17612945888142	-1.04446373505378	2.88339197886642	c
6.49702399326779	2.71852819741519	2.79917285966187	o
8.24142837103593	4.81785080957280	2.80960755230214	c
0.33590249065713	3.70082590677125	0.48160105645452	h
0.66202199528693	4.88910809106465	5.04847044705973	h
-2.14823172629109	2.66878865517255	8.06862755304460	h
-4.65458568679151	-1.04435222621145	6.65950226160392	h
-4.18527181982409	-2.74101698219510	2.33062692163531	h

-5.74454771138311	-0.86040253862950	-1.30660426042610	h
-8.39026198589982	-3.21255498649131	-4.30985795824657	h
-6.41879522304828	-5.88449659060673	-7.65530479331519	h
-1.72383282471366	-6.09803138596469	-7.93634641970244	h
0.93093247042968	-3.79594532896590	-4.93961995924506	h
2.89969862804601	-2.25947326541774	-2.10547415957207	h
2.31132893264968	0.90404984780393	-3.08741268158402	h
3.49083088064928	-0.82395682301150	2.37992613486695	h
9.55645033764711	4.66504636002796	1.22397720795713	h
7.21365861357233	6.60427179305466	2.66418898605381	h
9.23639343130749	4.68353358604080	4.60810644259301	h

10

energy: -711.5595933782

1.45220435507458	0.97138954351162	3.73617275054167	c
-0.53197609336636	0.17885983722980	2.14916763964053	c
-3.03065440397845	0.49990046840542	3.00450113398344	c
-3.50638903576962	1.52766167821904	5.38269106186493	c
-1.53320375525755	2.26697918326866	6.96275406267861	c
0.94815965353348	1.98341201621431	6.10767856807315	c
-0.03640929824847	-0.80208063199328	-0.26498274613938	n
-2.08009927343187	-0.86141190959857	-2.13456453805825	c
-2.96737903985215	1.73184190019095	-2.77625798122403	c
2.29815805698565	-1.93194789845751	-0.90383907142785	c
3.37127696084090	-1.51588202063242	-3.29400824869002	c
5.63283920411741	-2.68587484724468	-3.96533195496636	c

6.88465274596324	-4.25936415710341	-2.26097770536474	c
5.82929350044281	-4.66890224714751	0.12349983566647	c
3.55294755352431	-3.53699686224451	0.79660304155102	c
2.72127795028903	-3.89557205064359	2.63654536768108	h
6.76674719721844	-5.91148547737809	1.46286653115003	h
8.65500118678324	-5.15612783334058	-2.78173349260768	h
6.43313431218075	-2.33596843770846	-5.82366021387871	h
2.44283457729656	-0.24757291749267	-4.61493046983900	h
-3.68892729044678	-1.98096945754907	-1.43450045150321	h
-1.34409369474244	-1.89395415676993	-3.77964794387004	h
-5.33413571332368	1.85111803848871	-3.80339034201121	o
-1.64510737748423	3.25050481402188	-3.21364578022897	h
-4.61053988559012	-0.04349654156136	1.82095285049932	h
-5.45396862249591	1.74583368717168	5.99695237649049	h
-1.91684075327506	3.06378436851227	8.81380338443595	h
2.51870250080182	2.58053574720174	7.28875412433311	h
3.38974815816933	0.80948613543537	3.08757654747922	h
-6.13969442161901	4.35600811574577	-4.45164608362648	c
-8.01217369940716	4.16781593888845	-5.29181368302110	h
-6.23316122298371	5.53967430220754	-2.75380290546379	h
-4.83222433194882	5.20280167215266	-5.82178566414831	h

14

energy: -711.5679022740

-0.09718895677596	-2.10924610017311	-4.89046560539430	c
-1.40942340411148	-0.81491616683004	-2.97028523648717	c

-4.03612574467859	-0.47544048153822	-3.24006131213609	c
-5.29465612390353	-1.44877904074353	-5.33042624178986	c
-3.99306280657727	-2.76901515595448	-7.20914944271811	c
-1.38563834435214	-3.07197544974548	-6.97249847422232	c
-0.10426535116557	0.17724157027742	-0.90006860599115	n
-1.08672022463350	0.95277048548465	1.40647146993193	c
0.81399835145359	2.70235778930327	2.59974448851721	c
3.34206022148519	1.61168439442454	1.63562802216522	c
2.64039032011632	0.66276401521268	-1.02589277646014	c
-3.25913429762418	0.21810495516489	2.63251710041667	c
-3.64684049098264	0.97386495263842	5.16176745216097	c
-1.74057267038998	2.29844169112189	6.50825728184619	c
0.45629593083263	3.04850401597527	5.38053817928798	c
0.64596308435762	4.56563820001245	1.64072199062002	h
1.93028342712183	4.00233816691505	6.44359170853003	h
-2.02554656897646	2.67254294315165	8.50840148637467	h
-5.35748712789369	0.39087464694887	6.13056629285329	h
-4.59862655592685	-1.05004618893093	1.74062199170367	h
-5.06518914560702	0.60704497201581	-1.84034974571198	h
-7.31922534253628	-1.14962473319548	-5.50002254341912	h
-4.98955867947476	-3.52851227513874	-8.83321938755833	h
-0.32948276267763	-4.07317919681002	-8.42116843754362	h
1.92958047627930	-2.37762374374812	-4.75674875401118	h
3.67056813742320	-1.07637136685069	-1.46441848063588	h
3.05648559657121	2.06124252234938	-2.50125468620436	h
4.06192864230017	-0.40661153004786	3.26541582910262	o

4.85552042297507	3.03368777384742	1.56504594064677	h
6.58880717140402	-1.23165121337757	2.86777884082328	c
6.94946704154487	-2.70921447700654	4.26133221089392	h
6.86417419142598	-2.01870653921517	0.96197172059739	h
7.93322158299650	0.33181056446233	3.14565772381197	h

TS-10-14

energy: -711.5467454287

0.33114788829906	3.83155565030486	4.51155007869693	c
0.76155624708293	3.03210795821003	1.97252973296949	c
-0.74279597077261	0.98446425146048	1.01428696887620	c
-2.51678427726746	-0.20872982602974	2.53868947749015	c
-2.88801069069790	0.61925984144641	5.02638434245702	c
-1.42991480677785	2.60474848022309	6.01478494707059	c
0.13569158059624	0.05977528753147	-1.30724131526015	n
2.93083037125166	0.19935794289427	-1.54356164600239	c
4.03613160126288	1.00737305652081	0.94268626688840	c
-1.33747248899716	-0.97887353987727	-3.22272521999894	c
-0.21877737925118	-2.28093197331079	-5.26003243833371	c
-1.70746234840495	-3.28300010372071	-7.18691721920626	c
-4.32734999430851	-3.01744215070188	-7.15908107159266	c
-5.44198526442577	-1.68984535220461	-5.16670503647687	c
-3.98668669137935	-0.67431436594432	-3.23236317874614	c
1.40430955199343	4.45235429354168	0.62521788257046	h
1.36157016626114	5.45256561637151	5.23933419537440	h
-1.73426662995286	3.22719423610032	7.94774353290328	h

-4.28086225520525	-0.31932552622942	6.20508509573453	h
-3.54588586538390	-1.83432397174613	1.82809786791272	h
-4.88228871105097	0.39451272982722	-1.73145299070039	h
-7.47765306277126	-1.42043971550816	-5.12319013616410	h
-5.47632524541695	-3.80613815175640	-8.66414219431441	h
-0.79076736129671	-4.28332868667733	-8.72866010532397	h
1.81424716154026	-2.51190535736445	-5.35388374919113	h
3.65432858623414	-1.67878057825285	-2.04068584324993	h
3.46817017709921	1.52496942390702	-3.04613265724944	h
4.45024810261277	-1.00641044054356	2.52300418763119	o
5.53107746115065	2.43394149132865	0.93426391193069	h
5.52473890984441	-0.31761918283212	4.91529160121956	c
5.88382844425791	-2.08144280607144	5.91845626057905	h
7.30517135929540	0.71349739983003	4.63260008304083	h
4.19224143457909	0.85517406927313	5.97676836846388	h

15

energy: -821.8166625646

3.77567303798436	-1.94086207292754	-3.62880719248420	c
1.97229616617468	-1.85496326171031	-1.70929107100952	c
2.35954724888938	-0.31622530801870	0.41343073373152	c
4.57186865689408	1.12726817630467	0.58761730474414	c
6.36415254942490	1.00736836591518	-1.33569415402056	c
5.98278487580235	-0.51712021731656	-3.45437164634704	c
0.42531221030113	-0.18269121087081	2.29541436104561	n
1.01758885865545	0.09028813812635	4.82022292805506	c

-2.20390958822470	-0.60769111230725	1.44850313536332	c
-2.81280092260720	1.00111312154134	-0.77425073555033	c
-4.05242922694548	-0.05607253603765	-2.96873090937036	c
-4.57930364733280	-2.29748358355208	-3.25601710596985	o
-4.56628296528295	1.75475103057563	-4.73420319473571	o
-5.76481139831419	0.80712500630085	-6.99847793145281	c
4.88664725684527	2.31291192359647	2.22210815249892	h
8.07112667532945	2.13670556760968	-1.17570602650611	h
7.38826337688555	-0.59074506111630	-4.94761895525005	h
3.44309280193336	-3.14594363512071	-5.25688610017793	h
0.28217894691905	-3.00245446661969	-1.87465214633037	h
-3.42960841016223	-0.12449264537648	3.03962975762321	h
-2.53423910529135	-2.60042354870628	0.99409683865649	h
-2.31952758858916	2.99237251623262	-0.76135967149615	h
-6.03996212859903	2.46022993688807	-8.19457373394880	h
-4.54324785382540	-0.58075563865893	-7.91987327347165	h
-7.57003049793065	-0.08134662936664	-6.53095947890267	h
-1.11674469191506	0.10449304901061	6.28160791910358	o
3.13710024125814	0.29518483585399	5.69625994446155	o
-0.60758315413914	0.40048439624230	8.95002144810256	c
-2.46125513278036	0.36823382492083	9.84723906282165	h
0.57055414903431	-1.15313140016994	9.62667935240494	h
0.35354925960809	2.19387243875727	9.29864238841138	h

16

energy: -821.8213222188

1.29330609042369	-1.12478351509860	-4.68043590158134	c
0.55083247513289	-2.03089676216689	-2.10883071197895	c
-1.81847086261421	-0.80519512112437	-1.13227162533132	c
-3.49180831172236	0.45005699713879	-2.66353430780480	c
-2.83383913962560	0.84835597557008	-5.22081226958626	c
-0.41206646786238	0.15287487694481	-6.14143979584211	c
-1.84448197329986	-1.11663799566606	1.48772368920357	n
0.62708464507151	-2.02853087576674	2.44409582008169	c
2.41427402579703	-1.72137617586161	0.17830699990502	c
3.63325302105305	0.86835843289458	0.14673068165641	c
2.84634796386461	2.72716350609606	1.23593200183229	o
-3.81976672445081	-0.48460519566039	3.05470935031809	c
5.72193225664487	0.86341240114060	-1.33849482913848	o
6.89468449908992	3.31996189692566	-1.65592137284739	c
-5.22720147213520	1.20431788100420	-1.88673231502319	h
-4.14235654719438	1.83837832387113	-6.45022212633483	h
0.10528332410272	0.67976635263492	-8.05889826164070	h
3.14490365561663	-1.63874038360627	-5.39796548929851	h
0.21281234630465	-4.10186715557711	-2.20079942947554	h
0.48037679070199	-4.01197605639376	3.01695099351522	h
1.19821756108673	-0.89131883021487	4.06283455982017	h
3.88457212291605	-3.16834160483605	0.13051869699999	h
8.51649361716881	2.98655740062161	-2.87912171995615	h
5.55556749405430	4.62339256643514	-2.53236259194901	h

7.48377810723901	4.06381194983525	0.17716724917492	h
-3.18319872414789	-0.97866704426829	5.50766832460259	o
-5.85161526558126	0.36680437012363	2.38487995477825	o
-5.14520829440566	-0.33258827237345	7.29563627569745	c
-4.36984890592488	-0.83063911079770	9.13739173208381	h
-5.56546335776571	1.68599583690452	7.19161789062328	h
-6.85839394953813	-1.41304466872871	6.89567852749575	h

TS-15-16

energy: -821.8021730180

-3.15662666810509	0.23587810200108	4.66317018023751	c
-3.32646664180621	0.64514111473830	2.05531564430493	c
-1.26773740110808	1.66622908604782	0.79265315948388	c
0.98620606347029	2.38360992191678	2.13806180509750	c
1.08973148743880	1.91946019772773	4.79329339371307	c
-0.93515170668452	0.80715479377604	6.00927342294571	c
-0.91412958846424	1.70817032300293	-1.83232800578466	n
1.77305577915643	1.28016170596478	-2.60753853602628	c
3.22708582875052	0.35337934959459	-0.33544016418318	c
2.90624240234570	-2.29233615669395	0.37605534237113	c
1.26592263728831	-3.70117844259256	-0.44307742288432	o
-2.86250009259216	1.71777772575034	-3.55398206308556	c
4.60418555453686	-2.99958230353005	2.19068686920179	o
4.24655966824712	-5.54136585347104	3.12221602748344	c
1.76105784723341	-0.11798015465836	-4.12411894759992	h
2.58604710859037	3.03945460810979	-3.33126486400508	h

5.06888402452928	1.17663817998969	0.03143720102499	h
2.75832243634536	2.49048663931461	5.84152914888225	h
2.01960130797934	4.01921877907234	1.43464728808630	h
-0.84717523060753	0.44467501760561	8.02768237784842	h
-4.75457584711825	-0.59669706948636	5.64499551823527	h
-4.99695888636204	0.10567257874684	1.00746423559445	h
5.71793484668030	-5.80963086107348	4.53836603259741	h
2.36819041732542	-5.73015693936422	3.96045818602427	h
4.44476658397362	-6.90358841024521	1.58228023088027	h
-1.92110093761951	1.59216118672683	-5.95193741672726	o
-5.10602943167983	1.86056120160926	-3.05495628961584	o
-3.85253412420525	1.56737183916895	-7.88466280739265	c
-2.82003073696703	1.46583826152671	-9.66364236533753	h
-5.07759157776174	-0.07677021979171	-7.64515718261811	h
-4.98518512280966	3.29024579851592	-7.78147999875212	h

17

energy: -636.3046727064

0.88710006635211	1.45617584694423	2.01040985102335	c
-1.09384479045002	0.31863596645551	0.65221064051530	c
-3.50374178483473	0.28381121662736	1.77526434723876	c
-3.88260034516570	1.30402818533671	4.17121450773720	c
-1.91126094660802	2.39523060395843	5.55260964859965	c
0.47329734336943	2.45046897553434	4.40699968879451	c
-0.70270849739256	-0.64474812312581	-1.79060710238594	n
-2.89528270755894	-1.06612890100419	-3.42925743152676	c

-4.27885158707147	1.29722926838355	-4.07433258799227	c
1.68545701380495	-1.50350644645982	-2.62007645766512	c
2.45971324772199	-1.07515994938547	-5.12232423215856	c
4.77917793305433	-1.98231515881288	-5.97721720591469	c
6.38585626417892	-3.29768170100570	-4.35338458683908	c
5.62964147041607	-3.71457275157308	-1.85896356820895	c
3.30161085906300	-2.84618144214486	-0.99790362754066	c
2.70939912292045	-3.21113856511406	0.93130720764220	h
6.84747160465887	-4.75832858916405	-0.57655494543430	h
8.19928094059411	-3.98858109000789	-5.01916942908755	h
5.34309827213826	-1.62597147340721	-7.91918871635890	h
1.25389130381675	0.00281650574027	-6.38648062681663	h
-4.18544106891891	-2.45102794827300	-2.54948182059076	h
-2.17922636781221	-2.01561523406083	-5.13228809924984	h
-6.18352049532254	1.21094705322028	-4.83246439561903	h
-3.35897627946611	3.11816080429633	-3.88192008946014	h
-5.09988481094264	-0.52943590953141	0.78208734682449	h
-5.77231251810113	1.24040199925561	4.97955685188361	h
-2.34059414422664	3.47372816961243	8.15733844444893	c
2.04840707055347	3.32026177864860	5.40201140064305	h
2.75297940871039	1.57416070486944	1.16955304052571	h
-0.62744806322999	4.38390459245992	8.87261980306601	h
-3.85807540996393	4.88439729987093	8.15144804902698	h
-2.88261210428738	1.99603431185630	9.50698409487962	h

22

energy: -636.3257238403

0.98522838426942	-2.87362558087659	-5.36556939018074	c
-0.22342025843250	-1.33072038207445	-3.56371348365338	c
-2.70742461115268	-0.51220542145330	-4.06314766437586	c
-3.93972057149738	-1.26365920276036	-6.25819426843455	c
-2.75033332976606	-2.83023315825017	-8.01809735444611	c
-0.27538904639066	-3.60945520074639	-7.55411627288432	c
1.06095259582630	-0.57559714858745	-1.38416025342677	n
0.04853062746582	0.47836338924771	0.79724498635643	c
2.12403966660897	1.90219863791650	2.12260416217669	c
4.46679549763070	0.29965050323521	1.42140451159504	c
3.85779214374682	-0.58579525509947	-1.27484370557229	c
-2.31553583905271	0.19037697671271	1.84791505179074	c
-2.76919947345636	1.08684403145971	4.31395148677353	c
-0.78171182239747	2.09292145957229	5.84063497718226	c
1.58660963580391	2.39351731265043	4.85335697139513	c
2.35523109382289	3.74047662232194	1.13126020297769	h
3.11082029168289	3.13678087922749	6.01692130190668	h
-1.34082484463252	2.76101340691965	8.55791260539479	c
-4.63643744462796	0.85020879449620	5.13354252822115	h
-3.78933932044043	-0.84227636658460	0.86709146584600	h
-3.63389928171673	0.76485997177569	-2.75913191512904	h
-5.85009636854081	-0.59427204248976	-6.60430689264975	h
-3.72692700715538	-3.41464563641853	-9.72464062399321	h
0.69715771071860	-4.80915993531625	-8.90741530862071	h

2.90810230810444	-3.50938001825568	-5.05604457644070	h
4.58865088561518	-2.48373364647871	-1.64706627370739	h
4.61395405574457	0.69511060943501	-2.72223399297941	h
4.59483693180621	-1.32087618678014	2.70041150375046	h
6.23850686168540	1.35723351286210	1.50919845604967	h
-2.06765495165338	1.12101791276183	9.59580271304151	h
0.35549549252728	3.44091209380674	9.52355061106835	h
-2.78479001214656	4.24414906777062	8.66987844096802	h

TS-17-22

energy: -636.2921510967

-2.90127556194593	-0.52883098024706	-3.69401021163395	c
-0.34729370306413	-1.27254093530689	-3.50453707200128	c
0.69850311367084	-2.67857635179194	-5.51180694200854	c
-0.78062849932740	-3.36430186448259	-7.57909962981408	c
-3.31678254927206	-2.66963070306663	-7.72754954821398	c
-4.34942539686427	-1.23235989199191	-5.76793032234329	c
1.12036611567812	-0.57272327719161	-1.43707217315938	n
3.91930938445212	-0.85102551607173	-1.46749127593849	c
4.92511487544672	-0.29768770370797	1.12834673161157	c
0.21469414119705	0.51423341780412	0.80498171776084	c
1.87275601665845	2.33500654172959	1.90735657745711	c
1.38066357440173	3.18267047240878	4.40206506838973	c
-0.64081959268555	2.24766413297181	5.79203270314167	c
-2.26966068170663	0.48443683075685	4.63271777039809	c
-1.83719645175760	-0.39461862117787	2.18334056249249	c

-1.13738866423725	3.13008627740063	8.45806536503083	c
2.93760564740514	3.56864316284989	0.65644588886268	h
2.59265385831470	4.62705327486362	5.22001981891842	h
-3.87198549514827	-0.25188935563664	5.68796076701502	h
-3.03775764502397	-1.85403270067506	1.38603993510280	h
-3.72480656173214	0.63320427214126	-2.22114854762964	h
-6.31001033146892	-0.62735392711766	-5.86019341929365	h
-4.45880397796887	-3.21157096229392	-9.34288293823451	h
0.07790008292179	-4.45453693062112	-9.09313398831762	h
2.66962369055129	-3.23367403460903	-5.47238433563044	h
4.36300712202900	-2.79685468586568	-2.03898110133833	h
4.74692030439512	0.41866566208848	-2.88732587519756	h
4.62710695682621	-1.70982665797995	2.58804019144571	h
6.65184290338769	0.79796432534768	1.28436040324675	h
0.22106047286060	4.58093545448203	9.02557826176111	h
-3.04250105815442	3.92240042751652	8.64533889278724	h
-0.99279208983920	1.55907084747412	9.80285672533285	h

18

energy: -711.5545686225

0.29776392106048	1.51429654438795	1.50293539451550	c
-1.42944871209426	-0.20846055534493	0.49124566749295	c
-3.46420053872004	-0.99179068629521	2.00025097399831	c
-3.77224398504331	-0.06305086614317	4.43352451662769	c
-2.01773776315211	1.64635156389545	5.43838102091447	c
0.03438635439313	2.42459985028813	3.96355222927267	c

-1.18165663741100	-1.13149309115971	-2.02479226708546	n
-3.22817227603277	-0.51846267486616	-3.79246573237320	c
-3.01378403200697	2.04259388282337	-4.95977570776283	c
1.17472528278494	-1.86439069116190	-2.97772364849570	c
1.72544357072853	-1.74345589175160	-5.57788389371940	c
4.04375878688214	-2.59681419488452	-6.49582078639495	c
5.87384517736320	-3.56733718195822	-4.87085626019395	c
5.33770875978179	-3.68669549514894	-2.28646979130064	c
3.03053068868398	-2.86143768297602	-1.34501336175658	c
2.63218110703838	-3.00021816292355	0.66163021159866	h
6.72734250103563	-4.45476566379759	-0.98340801144528	h
7.67913241188960	-4.21831260215444	-5.59558451988655	h
4.41866850326185	-2.47389748801146	-8.51153149487922	h
0.36269698072036	-0.94183646764433	-6.88225831922849	h
-5.00592121370996	-0.68243700436044	-2.72404424240498	h
-3.30361285818381	-1.99491537784657	-5.24957797967934	h
-4.08812550613437	2.51110711417282	-6.64565061166282	h
-1.99462333672633	3.53445025259586	-3.99024012999369	h
-4.80358394047354	-2.36013030173785	1.25957499166389	h
-5.34386770814463	-0.66947644436519	5.60428371617299	h
-2.46390632052025	2.43569565050133	7.86584828246188	o
1.41505781407275	3.75176918672164	4.69154253776402	h
1.88395129480722	2.13324266116108	0.35785177643664	h
-0.70050894880498	4.17868149940715	8.93427610203130	c
-1.38125939312170	4.55473339502463	10.84248394134930	h
1.20216592577589	3.35702155714750	9.01743641113673	h

-0.64670590999979	5.94483536640509	7.84827898482617	h

23

energy: -711.5730489225

1.47698808165785	-2.93312859297994	-5.62708290661710	c
0.18817253940922	-1.42577875997704	-3.85060206046758	c
-2.33863496692382	-0.75732195824112	-4.35789867425104	c
-3.53226353713205	-1.61944166826069	-6.53393421582564	c
-2.26188121226531	-3.15208303765199	-8.26639758370248	c
0.25409928283201	-3.78195103385330	-7.79583761623325	c
1.43327087805846	-0.56343850457347	-1.68833324210154	n
0.36898897844671	0.49173576096687	0.46880163823127	c
2.36904991507608	2.04689130380979	1.76040795377715	c
4.78631538590844	0.54242569206247	1.09542542240032	c
4.22638313636794	-0.41943812644022	-1.58454440196777	c
-1.97237022056385	0.11500660231405	1.52741665090088	c
-2.49842661754051	1.05725585860012	3.97124548267803	c
-0.55763971561121	2.20656681225067	5.43216245546712	c
1.82167391523921	2.58415039652487	4.47329993461368	c
2.50727830404722	3.86209390697338	0.70835696788699	h
3.26394719951599	3.45436338202897	5.64508429320702	h
-0.94027123158480	2.97551501669255	7.89538339655985	o
-4.36035620007928	0.75382761782285	4.76297941093343	h
-3.39424321817351	-1.01665344641871	0.58077047799726	h
-3.33209979770712	0.49277674933902	-3.07735403911555	h
-5.47767448878636	-1.06407619622034	-6.88666757380809	h

-3.20839460895688	-3.82429883366548	-9.95747214598748	h
1.28931043782529	-4.95139846411322	-9.12898234174463	h
3.43364841829202	-3.45260483844699	-5.31299144850949	h
5.06013189486539	-2.27976815651715	-1.92881014517105	h
4.90998004903693	0.87901195372179	-3.05248501277602	h
4.98380455482378	-1.04449709074595	2.40701544147758	h
6.50548051794615	1.68423121161577	1.16836610545271	h
-3.30794766648115	2.38401083820545	9.04098292183095	c
-3.16724570142631	3.07232795827382	10.97950363488309	h
-4.87597660126931	3.34524242390550	8.08060581667909	h
-3.65309770484724	0.33844522299767	9.05158540330246	h

TS-18-23

energy: -711.5422495981

1.36013903754011	2.75663712969944	4.11453120233080	c
1.74733784535966	1.93580113272857	1.58836804180469	c
0.04069028721319	0.16272406524465	0.49538435292685	c
-1.97370330399575	-0.76731543814650	1.92180746989844	c
-2.33687844408223	0.05232929612783	4.39401672074785	c
-0.64027652566313	1.78206071476165	5.50552323598381	c
0.86214993859646	-0.89937048539633	-1.79589650273882	n
3.65074295013233	-1.22910250561108	-1.91649777123734	c
4.73483873984234	-0.75003181915416	0.66239107346223	c
-0.67229866112037	-1.45626260220825	-3.85525198364396	c
0.28662387017398	-2.79363803760804	-5.95187754721544	c
-1.26154486424400	-3.34788621601491	-8.00759203524483	c

-3.78295610177733	-2.58701951636395	-8.05678601770447	c
-4.72950661390712	-1.21899581755152	-6.00616872671504	c
-3.21211808274651	-0.64630854717270	-3.94141044969873	c
2.79886595162040	3.17654527441189	0.33438735675467	h
2.60881798294603	4.17786227092138	4.89939955575010	h
-1.17431998928325	2.42146685305436	7.96231140898666	o
-3.88741996434504	-0.66798712261353	5.52647221733411	h
-3.20941910507054	-2.19969519723151	1.12974825442737	h
-3.96923052448710	0.46393692162813	-2.39450526232277	h
-6.67658487789065	-0.56522864783443	-6.01810197923010	h
-4.97888439176841	-3.02642432954015	-9.66422686508337	h
-0.46964145060264	-4.38710955356218	-9.59226519872684	h
2.24387344733434	-3.39658075576362	-5.98850862086187	h
4.04441361350555	-3.16722203826446	-2.55060934309797	h
4.46196768768042	0.06173660436457	-3.32749623135023	h
4.44877958960169	-2.18550115173738	2.10172151232055	h
6.48288043884271	0.31452663655336	0.79648872215526	h
0.49814479898817	4.16748376788345	9.15927456332365	c
-0.20500265651343	4.37990117710194	11.08473775316119	h
2.43837135581203	3.43390931409050	9.19424677076800	h
0.47114802230820	6.00475862320282	8.19638432273585	h

19

energy: -696.2531637484

2.22098679323719	-0.41689261293190	-2.72854056942597	c
2.21119216934882	-0.32446201865841	-0.06707835779408	c

4.48806894166791	-0.82784511762166	1.21418028198974	c
6.67067145764215	-1.44108217052721	-0.12731380043530	c
6.65695230807001	-1.57008520733630	-2.75707432642671	c
4.40948174121111	-1.04293589604911	-4.03866970776736	c
0.02090344377094	0.34997747301569	1.26184344538706	n
0.22286229583397	1.07079550078836	3.92892100553434	c
1.71018255382145	3.42353692336494	4.34376492487275	c
-2.42515717891472	0.06879153412540	0.21608200644366	c
-4.25170501573087	1.95450860905917	0.58854035926718	c
-6.69670092088802	1.67282306662327	-0.34984568884205	c
-7.28436381486589	-0.49346438045107	-1.70530617771697	c
-5.51763072913956	-2.38992512400571	-2.12679978367826	c
-3.09462134263558	-2.10927485101464	-1.14222796870361	c
-1.70237169852594	-3.58884948717320	-1.41997780526023	h
-6.05187438430649	-4.06999480402323	-3.17385799598927	h
-8.11868068025117	3.12273885217305	-0.06947868242802	h
-3.74798824322177	3.66633275752274	1.60225503315316	h
1.02505291414973	-0.50789202708583	5.03441773274886	h
-1.72384826668531	1.27482085597582	4.62579182685565	h
2.38105426383381	3.90703555427976	6.22153729687416	h
2.06094144963802	4.71377907857780	2.79111430255866	h
4.57048698472421	-0.73078813637082	3.25809888845829	h
8.39905279329372	-1.82433107160724	0.91341289214148	h
8.36203804990284	-2.05170049301323	-3.79062559734960	h
4.35897751539966	-1.09416986114375	-6.09108636936449	h
0.50748500680225	0.02851262238831	-3.76153619973342	h

-9.66144840718250	-0.76995956888080	-2.65054096536957	f

24

energy: -696.2750485079

-1.43665655405859	-2.67625126484832	-4.15809735537524	c
-1.61713054151026	-1.12298037362377	-2.00560328580609	c
-4.03713487457974	-0.46745377460036	-1.11357014613011	c
-6.18838235961843	-1.38785969194953	-2.30851410670178	c
-6.00123920848451	-2.96432275129582	-4.41664344609784	c
-3.60872255009360	-3.58194637588273	-5.33403426353895	c
0.57058463247820	-0.19634210528134	-0.84493423914776	n
0.79434644336269	0.83061532556740	1.55652315334954	c
3.14047183318292	2.45019429029540	1.59194986423132	c
4.86436875736700	1.00792612177727	-0.27598087371179	c
2.98032506161539	0.02565275922179	-2.25232386136646	c
-0.62978687400070	0.37924569472567	3.69101898114683	c
0.21083903852407	1.29467115189562	6.04658954072615	c
2.59734317124925	2.49362560463449	6.20147674317186	c
4.11551672530150	2.98524806776752	4.19193251456845	c
2.65226407775029	4.26645282186217	0.65750572639504	h
5.93451141211334	3.89673548903745	4.44489715477280	h
-0.85621000419135	0.95789977082800	7.76133877905575	h
-2.30998361204871	-0.79041235977384	3.61421356942633	h
-4.21884802794040	0.81524147078635	0.47176428141531	h
-8.03435187184041	-0.84368489181583	-1.59252300178993	h
-7.68854047972798	-3.68030962496196	-5.33724678981427	h

-3.41849235386433	-4.78692965712522	-6.98553905326928	h
0.39829753562123	-3.18962830030297	-4.91089701282809	h
3.54637255204071	-1.81118845345062	-3.01203780444265	h
2.73749228599645	1.33915425652708	-3.84032749407892	h
5.76895046763866	-0.57093126171554	0.70684823356222	h
6.32822170899749	2.19526279250218	-1.11920908185964	h
3.40557360871981	3.13231526919951	8.57142327413698	f

TS-19-24

energy: -696.2406903096

1.14363987924885	3.56610358704658	5.37208901016660	c
1.65351409972321	2.72138278038539	2.88083132383218	c
0.05667673212617	0.84142123193574	1.77819212200363	c
-1.97029782873546	-0.14445831172878	3.14118335684668	c
-2.46061772972959	0.70482628363683	5.59386560780041	c
-0.85621674626005	2.50681348070036	6.66617823134593	c
0.99852777012259	-0.21090606257882	-0.46533785290463	n
3.80361906151312	-0.39364852528544	-0.50178292588508	c
4.79052969272039	0.17463554767886	2.09784002278969	c
-0.44726440518387	-0.92623828327668	-2.54589223470947	c
0.64071161661028	-2.28870253362675	-4.55976882699758	c
-0.81433998866904	-2.99448641739717	-6.63731594122929	c
-3.36669111274215	-2.36257705862408	-6.78759278290383	c
-4.44193035316005	-0.96858184303474	-4.82018369463432	c
-3.01777521585312	-0.24554922641096	-2.73596168068289	c
2.68127209244475	3.98430642447979	1.63010510906102	h

2.25595159855159	5.05514305281740	6.23636781837100	h
-4.01593267199922	-0.05378733910841	6.69185218800183	h
-3.11284237157587	-1.64327302573528	2.33415813279062	h
-3.87579295151916	0.88466377792210	-1.25788858881500	h
-6.41667732291806	-0.41246134146018	-4.91399685498145	h
-4.48962244354207	-2.91947241452992	-8.41108270360507	h
0.07557831789945	-4.05020898900880	-8.15744679281618	h
2.62512309548793	-2.79426197217041	-4.51814370210235	h
4.31534736397047	-2.31882350268211	-1.08622822369449	h
4.58675479448671	0.91444080844961	-1.91190594512946	h
4.52696899098883	-1.24827691682345	3.55364405956755	h
6.47993088411471	1.32515299157797	2.26582417909090	h
-1.34814484812215	3.29682379685155	9.06839758942306	f

20

energy: -749.7048666312

-2.62308408805177	1.86883895167170	2.05725285200577	c
-0.69025574811484	0.31816359522798	1.05959665781225	c
-1.30800367821210	-1.32188679702755	-0.95402537730006	c
-3.73804593351197	-1.38455763832651	-1.92248417850695	c
-5.66396449183854	0.16097873307597	-0.94055391884237	c
-5.04444558232636	1.77644881543600	1.07081840071310	c
1.72388800879987	0.31961287879678	2.09502888098360	n
2.19281487549512	1.65478296521662	4.48584868781284	c
2.46074014116027	4.44683386934285	4.18605279615066	c
-8.28578661587004	0.15448115120277	-1.92636137283368	c

3.86423124108424	-0.51367469260028	0.69628565303844	c
5.64114384663667	-2.12135041212926	1.82011400750517	c
7.77338283349370	-2.88706864970902	0.47731459650277	c
8.14414650869296	-2.08694683263164	-2.00729134808680	c
6.36679446277120	-0.49704794539197	-3.13662599699077	c
4.24894213193682	0.30104324330397	-1.79458905980722	c
0.13043851909379	-2.56410759448750	-1.72072803440152	h
-4.14435517118445	-2.68279361141804	-3.45929534905790	h
-6.52280613583038	2.98158257135547	1.82772367764846	h
-2.20269912514370	3.18224119177554	3.57338011298006	h
3.92633357526891	0.83723139682419	5.29020121377999	h
0.66969930096928	1.19067181276201	5.81649879877133	h
2.26481185298752	5.68522639864773	5.81100209374439	h
3.09771029944710	5.23748084685774	2.40515782964473	h
5.33282928422197	-2.79348514480299	3.73500530884772	h
9.13389522925614	-4.13805728651500	1.37064196871490	h
9.80027990794454	-2.69631626481230	-3.05400274139384	h
6.64395525443382	0.15202646754275	-5.06443093001740	h
2.87608709747472	1.55498754481718	-2.66165162182416	h
-9.92513182892891	1.51785201405033	-1.00446843509362	o
-8.91994528030322	-1.58830575022207	-4.12427848357632	c
-7.75649750583061	-1.14340961169441	-5.77970769869876	h
-8.55558734700576	-3.56640850145053	-3.62721034364601	h
-10.91151583901594	-1.35506771468839	-4.60021864657893	h

25

energy: -749.7222846762

-2.62308408805177	1.86883895167170	2.05725285200577	c
-0.69025574811484	0.31816359522798	1.05959665781225	c
-1.30800367821210	-1.32188679702755	-0.95402537730006	c
-3.73804593351197	-1.38455763832651	-1.92248417850695	c
-5.66396449183854	0.16097873307597	-0.94055391884237	c
-5.04444558232636	1.77644881543600	1.07081840071310	c
1.72388800879987	0.31961287879678	2.09502888098360	n
2.19281487549512	1.65478296521662	4.48584868781284	c
2.46074014116027	4.44683386934285	4.18605279615066	c
-8.28578661587004	0.15448115120277	-1.92636137283368	c
3.86423124108424	-0.51367469260028	0.69628565303844	c
5.64114384663667	-2.12135041212926	1.82011400750517	c
7.77338283349370	-2.88706864970902	0.47731459650277	c
8.14414650869296	-2.08694683263164	-2.00729134808680	c
6.36679446277120	-0.49704794539197	-3.13662599699077	c
4.24894213193682	0.30104324330397	-1.79458905980722	c
0.13043851909379	-2.56410759448750	-1.72072803440152	h
-4.14435517118445	-2.68279361141804	-3.45929534905790	h
-6.52280613583038	2.98158257135547	1.82772367764846	h
-2.20269912514370	3.18224119177554	3.57338011298006	h
3.92633357526891	0.83723139682419	5.29020121377999	h
0.66969930096928	1.19067181276201	5.81649879877133	h
2.26481185298752	5.68522639864773	5.81100209374439	h
3.09771029944710	5.23748084685774	2.40515782964473	h

5.33282928422197	-2.79348514480299	3.73500530884772	h
9.13389522925614	-4.13805728651500	1.37064196871490	h
9.80027990794454	-2.69631626481230	-3.05400274139384	h
6.64395525443382	0.15202646754275	-5.06443093001740	h
2.87608709747472	1.55498754481718	-2.66165162182416	h
-9.92513182892891	1.51785201405033	-1.00446843509362	o
-8.91994528030322	-1.58830575022207	-4.12427848357632	c
-7.75649750583061	-1.14340961169441	-5.77970769869876	h
-8.55558734700576	-3.56640850145053	-3.62721034364601	h
-10.91151583901594	-1.35506771468839	-4.60021864657893	h

TS-20-25

energy: -749.6904253359

0.18476643514136	-3.16037147765926	-6.08408211072053	c
-0.67735809731071	-1.55486004226210	-4.14692062288439	c
-3.17487118179122	-0.64075835763677	-4.28570155244450	c
-4.76084054298125	-1.37633414125939	-6.24488492946352	c
-3.91051860799847	-3.00763092442082	-8.13807846588801	c
-1.42451067637300	-3.87127718651098	-8.04320887833363	c
0.91709815776801	-0.80263654417514	-2.17559869106239	n
3.71858766635034	-1.04681846266579	-2.36451915893594	c
4.84272488877092	-0.68493229924305	0.21479247619667	c
0.12383053608668	0.21501611036320	0.11613100607476	c
-1.95516148946552	-0.64410262046660	1.50276626736950	c
-2.27436368099075	0.12925165287635	3.99251561133455	c
-0.52830637879231	1.76898982757850	5.17073175009267	c

1.50054863338660	2.70426258529383	3.76620757953248	c
1.89243789463735	1.93644158578173	1.23642340304752	c
-0.96841596125812	2.48931614394941	7.85285491971243	c
0.94591871746528	4.19549010723290	9.15013057555641	c
-2.82862985257582	1.72367939293831	9.00893070433643	o
2.92874984629367	3.20461932752032	-0.00509910682960	h
2.78106074682474	4.08213117369250	4.58517303277349	h
-3.84674323677585	-0.56312647840620	5.11218367931546	h
-3.24472005382820	-2.00314105255308	0.66917298742278	h
-3.84720671044210	0.67472193257650	-2.86549768860774	h
-6.67691950215934	-0.64069329608075	-6.30374077123460	h
-5.15495402545891	-3.57141924193118	-9.66791043741656	h
-0.71177246904265	-5.12000537797222	-9.50897268878320	h
2.10844589391295	-3.86318621439436	-6.07705923304514	h
4.14165472739830	-2.93901905005498	-3.09486763769412	h
4.46284727984110	0.33147035577731	-3.72624299711738	h
4.57475775782918	-2.18931559244685	1.58594115442786	h
6.60093822757027	0.36020787783288	0.36448631174668	h
2.83425786818012	3.34507908083795	9.12322585026895	h
1.07685974273430	6.02237931219786	8.18111766212462	h
0.34980744705339	4.49657189369110	11.09959999912738	h

21

energy: -613.4172201834

2.82532308080493	-3.02432716796671	-0.33799547000061	c
1.41483023842417	-1.16884192459257	-1.57481950236684	c

2.33051858047798	0.01317599547418	-3.74705714242968	c
4.69653783597079	-0.67221115659204	-4.68335550358856	c
6.12901512786033	-2.51232190558134	-3.45144034644294	c
5.19488988762818	-3.68504302998009	-1.28161129537152	c
-1.06258811788540	-0.51681275298920	-0.63082379000506	n
-3.27670371669635	-1.29844386976999	-2.16914806570518	c
-4.77118685319209	0.87422918471888	-3.14797961646429	c
-1.36066336252714	0.69521521225191	1.58370393360932	c
0.75607089312032	1.61689704424057	2.98235600506191	c
0.40345616650563	2.84643392872894	5.22136851059231	c
-1.96574138338031	3.20712738936071	6.16543623880847	n
-4.04107007058976	2.37121546264210	4.89818259541889	c
-3.80569415235200	1.14021181999349	2.64107111455631	c
2.06173924219259	-3.94732070232137	1.32889002831083	h
6.29762452370934	-5.13182085740400	-0.33413055428523	h
7.96927722164652	-3.03900825541065	-4.18769960773826	h
5.42157287963110	0.24037142271232	-6.37090334776588	h
1.21053580584849	1.45279068050430	-4.68700959644258	h
-4.45492942349376	-2.58972471924366	-1.04658012407951	h
-2.48854908751578	-2.44252869833928	-3.71251783569689	h
-6.68758998831512	0.56466633536762	-3.80748184796790	h
-3.86145319692060	2.66709397382354	-3.54872358397550	h
-5.50207079059868	0.52837131176146	1.67984902291327	h
-5.85255341325901	2.73697405762375	5.77762977558963	h
1.94972757971358	3.59093107826173	6.33619990416154	h
2.65580171967037	1.36254939415629	2.26899151985273	h

-2.18612722647841	4.12015074856881	7.83559858145129	h

26

energy: -613.4358617308

1.44643505798595	1.94329319232399	6.00930510466222	c
1.93422217046204	2.23307065378013	3.25630978054823	c
-0.03511058039874	0.85494306316695	1.70608724027952	c
-2.45693568751548	0.40149851673835	2.64295924026137	c
-2.87711099854857	0.80060341986738	5.20049421919771	c
-0.95062151124558	1.46712748743720	6.77450079411561	n
4.43519840316720	1.21423413015623	2.14113156987620	c
3.72310366384053	0.80465650748031	-0.64131697746162	c
0.97364163950988	0.20489778505270	-0.51468482829777	n
-0.36861974273902	-0.83312254688071	-2.62080123225834	c
0.54645549726576	-3.00889933798068	-3.80420051118256	c
-0.76443840858709	-4.00115874312381	-5.85940760403416	c
-2.96313938610097	-2.82974327816640	-6.72642233821494	c
-3.85455045557238	-0.64924664572419	-5.54066851590688	c
-2.55802406104032	0.36459427184598	-3.48643571591074	c
1.80314000636266	4.26542262976037	2.74796422492474	h
2.83468578456843	2.35268557490521	7.45369738577427	h
-4.68450047941168	0.46588442592973	6.09591081984113	h
-3.94916181513627	-0.37864490630714	1.48278308574316	h
-3.20556925370965	2.09256900154547	-2.58947978537268	h
-5.54659077314384	0.28254712597831	-6.22988526549518	h
-3.97339642127999	-3.60884471899365	-8.33193716754490	h

-0.06976743705683	-5.69849810485583	-6.77695435126998	h
2.23676037358752	-3.93932245956109	-3.10765128421676	h
4.73035558179078	-0.75646091524567	-1.53233498816733	h
3.98053250619401	2.50251239408186	-1.79942366209598	h
4.92279833713642	-0.58777169129179	3.02892150614283	h
6.01074939409628	2.52424058518457	2.36450120689617	h
-1.32054140448114	1.51693258289612	8.65703804916666	h

TS-21-26

energy: -613.4020230738

-2.63442855649047	0.60727614057047	-3.38791871622323	c
-0.46164825324300	-0.65726648090810	-2.57613521848434	c
0.25797550151789	-2.94723318177040	-3.67761128979161	c
-1.22098498243903	-3.97404731658416	-5.59827328373941	c
-3.40357042669417	-2.73255372180240	-6.40671891736858	c
-4.10905871918474	-0.44658672077594	-5.29727506674971	c
1.03245462373849	0.43076567524609	-0.60251651418990	n
3.82658561183785	0.74090958893268	-0.84879174781854	c
4.94010334505842	0.40879486072807	1.73785772120774	c
0.12453502808057	1.04951282031079	1.68497938138230	c
1.82925809634274	2.53209844009822	3.25565152908013	c
1.21974771366245	2.78280371448481	5.81309880781237	c
-0.84486361818946	1.58814580906952	6.77425210227276	n
-2.55757732063924	0.37765566426437	5.27998421369627	c
-2.15533097371428	0.13751402433491	2.72464972548510	c
2.85413999386031	4.09625906656878	2.40801924752951	h

2.30267536036389	3.92917986322617	7.11584532587039	h
-4.18697549339998	-0.41430621224394	6.22967584138688	h
-3.49864586938473	-0.89606352026106	1.57904744711244	h
-3.13638252907958	2.41244959909157	-2.55010740821138	h
-5.79078201222060	0.53698749566425	-5.93835716911541	h
-4.54611743517527	-3.54110200411668	-7.90545269218494	h
-0.67284288223123	-5.75629826603954	-6.45216752592105	h
1.92979166360475	-3.93953579568636	-3.02091611763842	h
4.47885350300735	-0.68801934448050	-2.19287704437185	h
4.25953052910890	2.60186096099819	-1.65195595471932	h
4.73579657337110	-1.43326090941397	2.62449051597540	h
6.62765120885084	1.46690329115410	2.22662196807491	h
-1.19988968031916	1.72715645933805	8.65290083963797	h

27

energy: -675.6464463011

1.06304001190487	1.21137871141958	1.82231989408571	c
-0.96345847839601	0.25735990848768	0.38040366934324	c
-3.39977404268327	0.32505454032389	1.42192295606108	c
-3.81219869715557	1.28018760577680	3.85617166622905	c
-1.77017683093244	2.17199009754523	5.25931549786698	c
0.68188927237476	2.15025845102158	4.24653692499531	c
-0.55658863490500	-0.62646395707123	-2.08909849051430	n
-2.72724980384473	-0.89386091634032	-3.78945968679939	c
-3.99874107561061	1.54872544292319	-4.36589666412591	c
1.79546479626260	-1.61731061106021	-2.87894527168536	c

2.68236444793313	-1.13857898530191	-5.33362212461385	c
4.96393761465698	-2.16569461704454	-6.15376217525199	c
6.42081967669598	-3.65534806103724	-4.53879199969748	c
5.55197822148560	-4.12492752932680	-2.09101585810657	c
3.25831236918373	-3.13607810772681	-1.26747914643436	c
2.57559005023751	-3.53751338332546	0.62419540000687	h
6.65173875099191	-5.30298032760254	-0.81824519860717	h
8.20594981654575	-4.44023515129609	-5.17654790346286	h
5.61854778120281	-1.76773858215497	-8.05885102971405	h
1.59475813988666	0.07192068184264	-6.58508265812742	h
-4.09178405974832	-2.25878659398970	-2.99439442836532	h
-2.00923352043157	-1.80047512457424	-5.51492423435008	h
-5.89449990746980	1.57189699129004	-5.15036937966118	h
-3.00081733189489	3.31924352361510	-4.10456375232213	h
-5.00615321155360	-0.35734028231122	0.34685581041063	h
-6.45778372811475	1.32875166545754	4.92549822423393	c
-2.08463281623069	2.91288443366452	7.15154419022191	h
2.87835998955168	3.15833457455422	5.76367228938874	c
2.94836078554395	1.23584271049882	1.01464923448602	h
4.62999994809549	3.10532877042226	4.66796438858917	h
2.54213758270661	5.11577357266850	6.35246646306920	h
3.16429845441212	2.04139752793379	7.48585335770402	h
-6.46841063688133	2.08972438028616	6.84691719479015	h
-7.70925494915325	2.50098196398362	3.76181385263664	h
-7.27678998466649	-0.57370332355169	4.98294898772067	h

30

energy: -675.6690920089

0.99638908658727	-2.92101292195943	-5.69200950692718	c
-0.21409399506283	-1.42171260313896	-3.85379482865328	c
-2.70735957484509	-0.61348714555135	-4.32685637134979	c
-3.94345301230464	-1.33081383401336	-6.53104820525061	c
-2.75170624323281	-2.85441388475240	-8.32695614888977	c
-0.26909875306756	-3.62394844462761	-7.88859731050546	c
1.07804084984124	-0.69804224453344	-1.66994912477446	n
0.07523432326762	0.32405737122888	0.53576055306273	c
2.15792678179053	1.74062833417470	1.85643256648069	c
4.50144609182455	0.14895762719048	1.12305465594683	c
3.87292666853267	-0.72006415453554	-1.57292649887970	c
-2.28060912881824	0.00780112846419	1.58362894877612	c
-2.76590705337518	0.86705866736046	4.07086252602336	c
-0.74025442212516	1.86849201247050	5.52907567498806	c
1.65190185434730	2.21617266189117	4.60805037772872	c
2.39156467415398	3.59824492964832	0.89647609782723	h
3.77462958974689	3.24652471053421	6.17270677618173	c
-1.11319384301699	2.34407760751998	7.49818030815490	h
-5.37012901683696	0.63972034485677	5.18429262514328	c
-3.74349638710119	-1.03326741470941	0.59208423037762	h
-3.63859707835162	0.62888179761691	-2.99331587320413	h
-5.86056661823783	-0.66924253365431	-6.85496369641359	h
-3.73239501931062	-3.41362194491612	-10.03959872246487	h
0.70624913570615	-4.79036102216070	-9.26884881608571	h

2.92549575799985	-3.54822544287250	-5.40456480937346	h
4.59611124156605	-2.61963945619132	-1.95467093574863	h
4.63042490267030	0.56261420598495	-3.01815661105419	h
4.64542941121714	-1.48256984290222	2.38756545797720	h
6.27303377846698	1.20651928497409	1.19833663655610	h
3.15559816084356	3.64797970983352	8.10366978189632	h
5.36969004961647	1.91935420425115	6.27203533258001	h
4.52738705071372	5.00491074804521	5.35321806313250	h
-6.42666865721734	-0.91437908704029	4.31486766150757	h
-5.29138889146741	0.30958737309508	7.22688935042161	h
-6.48056171452064	2.37321925841858	4.88906983481194	h

TS-27-30

energy: -675.6348284304

-2.78087137572437	-0.25254387277104	-4.07134033432492	c
-0.35417190789260	-1.32302978970466	-3.79124873520822	c
0.57225231391839	-2.85322541101985	-5.76601793567345	c
-0.90618246098439	-3.33936957096612	-7.88995722697807	c
-3.32276439080185	-2.31989494653021	-8.12813431048864	c
-4.23085451278815	-0.76063972030275	-6.20013890101140	c
1.11565843069775	-0.81454623166000	-1.66967411704354	n
3.85672200690797	-1.45654868039907	-1.60448463584437	c
4.85286252267392	-1.04850101265399	1.02005069787329	c
0.26506502085364	0.35318279277627	0.55276470489083	c
2.08894066704971	1.94966820975001	1.72480025867281	c
1.63898392736408	2.84410574488710	4.21992059082553	c

-0.56049991820834	2.11618241049276	5.45031359073672	c
-2.39451574951145	0.58413333937968	4.27217908129119	c
-1.94243154498178	-0.31301059342492	1.82583236930093	c
3.54413587609109	4.55877712572590	5.45371915323408	c
-4.78210065142511	-0.12320922743324	5.65762147818192	c
3.34550488779279	3.04429115971089	0.52089314446419	h
-0.90770104067828	2.79507508916397	7.36128172300680	h
-3.26899401569074	-1.61346073015362	0.95353168506349	h
-3.49955951693490	1.00526647015641	-2.62251125166839	h
-6.09099932143983	0.09527875347933	-6.36104211576652	h
-4.46524536466436	-2.70824242792604	-9.78680023382028	h
-0.14162952063027	-4.53234685390408	-9.37671927893029	h
2.45079965173207	-3.66186996537385	-5.65745833674518	h
4.05546191952989	-3.44339726544657	-2.17249437424849	h
4.88663469293115	-0.30656271286012	-2.99320744393403	h
4.33781216746156	-2.41551822167952	2.46194728615094	h
6.69523237284502	-0.17269843665935	1.23088732456450	h
2.84381671634484	5.28573958269169	7.25702169588030	h
5.32380759669844	3.55366012087949	5.81206892255830	h
3.99747632899069	6.17270087671608	4.23358109106409	h
-4.35902742181419	-1.06052164394554	7.45677423842425	h
-5.91178757294839	1.55903477300259	6.09897658637924	h
-5.95183081276325	-1.39795913399327	4.52706360912366	h

28

energy: -826.1489596366

0.95567235530154	0.87659610447085	1.44473233387747	c
-1.14612562663112	0.02781793747735	0.03402563341994	c
-3.57231832720272	0.23439088641953	1.05837111292950	c
-3.89001649425584	1.24522058037142	3.48723138554392	c
-1.83937410004704	2.04745530651875	4.92354746302149	c
0.58166198780446	1.85062562578268	3.85483083538808	c
-0.79802746768686	-0.90842825285440	-2.42156083447241	n
-2.97619862911768	-1.03671005827182	-4.12751137838809	c
-4.04735398010516	1.48509606782844	-4.77066593957827	c
1.53485185530644	-1.89264986940591	-3.26663115759466	c
2.38845888482803	-1.37227534701737	-5.72489961275940	c
4.65187702503516	-2.39673198207107	-6.59768478955525	c
6.12321993765849	-3.92534739874252	-5.03310126972169	c
5.28494632670949	-4.44134612292735	-2.58369138050805	c
3.00898677251331	-3.45631823557573	-1.70791483133851	c
2.34509003547914	-3.89765964258663	0.18145318197159	h
6.39324878331912	-5.65504230771939	-1.35230033799291	h
7.89430545416782	-4.70762102735005	-5.71193802526826	h
5.28073894842112	-1.96693326290311	-8.50452393145043	h
1.28665566490906	-0.13528471184867	-6.93729962303608	h
-4.44326850883695	-2.26640445022290	-3.29794111552791	h
-2.33218643060665	-2.04674899561845	-5.82452521143255	h
-5.90369033655140	1.63316819623995	-5.63185550905665	h
-2.96806353539332	3.18989027856546	-4.41604258870534	h

-5.24444232100642	-0.35694559161075	0.04119250224300	h
-6.34709765445059	1.35382135448863	4.30631733999792	o
-2.02734305974031	2.82731590496002	6.80478730292333	h
2.50927178294681	2.71586321234351	5.35826487108795	o
2.82042463231022	0.79535592844893	0.61465481521829	h
5.01567033293593	2.53747962242473	4.36908943241179	c
6.24695298614630	3.26918700796526	5.85107992266333	h
5.51164663988999	0.56856163283509	3.94784456119369	h
5.22381144669294	3.68342663820622	2.65347762228111	h
-6.77281184593699	2.34661950040088	6.78035620473253	c
-8.81249359597284	2.26585991304164	7.06489057898324	h
-5.81590620116113	1.19834439343266	8.21769335157652	h
-6.12077373767227	4.31035116450407	6.91624708492193	h

31

energy: -826.1701209755

1.45674341934768	-2.97065049307652	-6.01447530026877	c
0.20239162299641	-1.45406277607399	-4.21605295906061	c
-2.28259809702336	-0.65184460959918	-4.75874279023755	c
-3.46257028787374	-1.38782842696242	-6.98690830386185	c
-2.22774170111656	-2.92602856801102	-8.74196965186491	c
0.24504010603193	-3.69007070913452	-8.23479467371093	c
1.43346803973135	-0.71815229664294	-2.01089687283308	n
0.36871265747537	0.34784418434381	0.15076798096025	c
2.42288723395037	1.79234907963493	1.48103606710888	c
4.77862424430853	0.16232333982237	0.87705161041030	c

4.22223275477854	-0.79502781044188	-1.80775584006064	c
-2.01807544955222	-0.01974782101709	1.15319383974967	c
-2.50838612721417	0.84865211793941	3.61184400138864	c
-0.56147148600144	1.90523012823073	5.14886816861183	c
1.80752131033288	2.26287042150339	4.19642123280340	c
2.71133293405293	3.64390245072048	0.53130563797802	h
3.83458977710508	3.20609051803471	5.49567867435422	o
-1.03856511579698	2.37851239151606	7.08416842100012	h
-4.82126603796890	0.67387058123534	4.78822233285725	o
-3.41942721614293	-1.10011839359426	0.12788878757404	h
-3.24434480665605	0.60670886444740	-3.46161126992076	h
-5.37094075807692	-0.72821044619673	-7.36433022983319	h
-3.16561895699948	-3.49934393790635	-10.47373948021812	h
1.25476131205465	-4.86627294748534	-9.58197945929992	h
3.37846224681293	-3.59472579629927	-5.67375554559515	h
4.91667876663604	-2.72404512756395	-2.08752445461984	h
5.05921312771487	0.41157988549816	-3.27381171121347	h
4.87190310660445	-1.42108620168068	2.20361430848888	h
6.54676400062818	1.21988156906550	0.97985672467634	h
3.43637429939459	3.78950417284307	8.10427196729468	c
5.25050985553271	4.46331809153535	8.81190157417534	h
1.99821402969057	5.26851361566798	8.31270303766517	h
2.84555416934132	2.09870624651495	9.14889283589340	h
-6.83158067020308	-0.42817073119300	3.36949468198368	c
-8.49664312648072	-0.31161162677536	4.57899167184588	h
-7.16225037977087	0.62108711911823	1.61025840266466	h

-6.43049879764372	-2.41394605801729	2.92191658311410	h

TS-28-31

energy: -826.1358653125

0.62160481837976	-3.10228189171866	-6.60068794350603	c
-0.28544010304343	-1.59020945535898	-4.60289496403328	c
-2.72468333947714	-0.53745717150894	-4.84044761500725	c
-4.19988403240135	-1.03957987652348	-6.95326337067144	c
-3.30871904606110	-2.57785258824014	-8.90582539201283	c
-0.88285392199465	-3.58301475165593	-8.70779103764145	c
1.22030960103848	-1.07514313636805	-2.50804025284537	n
3.95502706188436	-1.77986028401650	-2.46757843639758	c
5.00613172359130	-1.37867835598178	0.13410659083287	c
0.39963807877878	0.05142235644073	-0.25804573637611	c
-1.79330206540256	-0.62198982449474	1.01753723143562	c
-2.18849861056273	0.28912513616288	3.47149456347189	c
-0.38301154849002	1.80501394287094	4.69972805027046	c
1.79612844529003	2.48829445865886	3.38617872605274	c
2.26797739439959	1.61405102038356	0.89900143108533	c
3.61726319291502	4.06538136803723	4.33665792426172	o
3.21254214561091	5.08163887196799	6.80435011161908	c
-4.40342616402622	-0.46501540027431	4.58707117175139	o
-4.84838846989118	0.31071970777818	7.13298351330043	c
3.49280085052563	2.76781681961461	-0.27436838870868	h
-0.71325158723412	2.48343094748120	6.59861072004832	h
-3.14673460372703	-1.92775921589332	0.20713498551391	h

-3.43568920873269	0.69930082543497	-3.37031136112149	h
-6.06925761091232	-0.19795165995703	-7.07964249600461	h
-4.47240042570006	-2.96325321360421	-10.55050775007159	h
-0.13106261153146	-4.76137606318048	-10.21273502941195	h
2.50660460148021	-3.89891761605384	-6.52460252842490	h
4.08989652850194	-3.77510176688788	-3.02356080977408	h
4.98266533263556	-0.66613143211458	-3.88582522865303	h
4.53285451474764	-2.75846505529757	1.57816713953212	h
6.84237341418194	-0.48562379587159	0.31406922657365	h
4.83602529421326	6.29458731078586	7.17739979520260	h
1.46762110143395	6.20099188568804	6.86978110714831	h
3.12481071782830	3.57251462945386	8.22421461114024	h
-3.36473486659944	-0.39634137371017	8.39824941001348	h
-4.95954103725046	2.37689608621394	7.27603452034035	h
-6.66139556439792	-0.51918143826202	7.65335751106522	h

29

energy: -795.5446526646

2.16616428284433	-0.54652116793435	-2.69290137487222	c
2.18146982514830	-0.39083243230971	-0.03204186470573	c
4.48266478726835	-0.76306518965624	1.25858298244370	c
6.63989821822602	-1.30418091025964	-0.12604863958193	c
6.69527221691671	-1.50993121562406	-2.73963074049269	c
4.39255667161683	-1.10592321117884	-3.94176659348417	c
-0.01058767421289	0.20535642444508	1.30161494500373	n
0.18714094444331	1.00369715469750	3.94979468752274	c

1.55476299659117	3.43949996450054	4.29591926457096	c
-2.45592834830334	0.00300307807869	0.22323115868971	c
-4.23019674080713	1.93489443007703	0.59840353317341	c
-6.66433370494496	1.71175466759393	-0.38251378592112	c
-7.35323978240574	-0.41604920270871	-1.77692285400566	c
-5.58637923085325	-2.33863455520619	-2.15950079653971	c
-3.16250062482793	-2.14781855764520	-1.15532940516722	c
-1.80711217082319	-3.66445402831644	-1.41862832283661	h
-6.10704730471575	-4.01895167000523	-3.21784179145138	h
-9.24502430537268	-0.57818788025299	-2.55403889364484	h
-8.01583095045706	3.22686287232246	-0.07855636258517	h
-3.68874006342328	3.62108163990918	1.63679552825530	h
1.08339309798234	-0.51002771156271	5.06934265715300	h
-1.76006487814347	1.11707453587371	4.66439478171164	h
2.21558516748386	3.99884695583491	6.15586934244260	h
1.82173349442264	4.71385059374300	2.71383473645435	h
4.63003678678185	-0.63340766655025	3.29288932680988	h
8.40900400798369	-1.93757976249868	-3.77043130408856	h
0.46367355025917	-0.20543036599579	-3.77397697707586	h
8.83144029727757	-1.66423391683793	1.16664183982490	f
4.33218943404449	-1.24069287253300	-6.50718507760321	f

32

energy: -795.5631842515

-1.47871206824636	-2.61774536345286	-4.17369458711762	c
-1.63868913352514	-1.05825174432552	-2.02370282594577	c

-4.04908190003598	-0.33752722756503	-1.15513849217530	c
-6.21189613051315	-1.20008066742824	-2.37220484354072	c
-6.04689968394252	-2.78147402196554	-4.47837568205941	c
-3.66295124644353	-3.46375666214441	-5.37153733123581	c
0.56219843754794	-0.18985786991821	-0.84442882722732	n
0.78793963000593	0.84212173629208	1.55513792987491	c
3.20524294302572	2.36422073389454	1.59469554165864	c
4.90739591464938	0.81135356748789	-0.20774320758100	c
3.01288757729772	-0.13071507515064	-2.19531292428513	c
-0.68054500769239	0.45244767194236	3.67146402813382	c
0.22564998329428	1.34947027457115	5.99555291288776	c
2.63185725146975	2.45353980002040	6.31665287044077	c
4.08214658711122	2.81732422790310	4.22271745970751	c
2.86186893267679	4.21321414722096	0.65761955288736	h
3.30228215401431	2.97049057963090	8.18220576544619	h
-2.39886091282889	-0.65850076745988	3.63320965949298	h
-4.21596458221870	0.94958741685990	0.42793403075978	h
-8.04922424600299	-0.60771070735409	-1.67265530576736	h
-7.74368677509163	-3.45278528056691	-5.41489411275773	h
-3.48850213294860	-4.67354759906930	-7.02122799178109	h
0.34855102039457	-3.18035530731500	-4.90954435793377	h
3.50687805824635	-2.01803894206432	-2.87849228573467	h
2.87466199987340	1.13737040370324	-3.83124760073017	h
5.72669804917677	-0.78403808300911	0.82051302151190	h
6.43111008070061	1.92246764090319	-1.04293289401617	h
6.43109678157062	3.85699426460752	4.42623889542372	f

-1.23345158156551 1.01378285375186 8.08919160166367 f

TS-29-32

energy: -795.5307461440

4.98637431581712	-2.43624531263889	-3.38754776205849	c
3.82793438080705	-1.68076213811922	-1.11035543788075	c
1.31674159226184	-0.68249331854920	-1.29008658566078	c
0.10496895349454	-0.47209827934341	-3.62293274183487	c
1.39602677306295	-1.26681581446182	-5.76913710672352	c
3.85976272431364	-2.21509018070236	-5.73219205401780	c
0.46887773160340	0.44639471537419	0.93469901256965	n
2.53402218723761	1.66242600462375	2.42487810772765	c
4.89543560334964	1.75321898149706	0.85784520504440	c
-2.04091017722502	0.50818577381593	1.75633693945946	c
-2.80275550434596	2.13996785740623	3.71521775157467	c
-5.30577222882377	2.16862403570227	4.53271988676507	c
-7.10714615313623	0.58364699320309	3.44810852746499	c
-6.35001065913706	-1.07365367366634	1.53829269592533	c
-3.85981631374582	-1.13100364028200	0.70677486128512	c
4.42513431382645	-2.62460046000237	0.61096745776835	h
4.80336138510997	-2.81501022008308	-7.44632900195852	h
-1.73900038469525	0.39847661117786	-3.80204440096514	h
-3.29909627048728	-2.47214860426130	-0.73710084909833	h
-7.70883114666711	-2.36118735908044	0.69425603091163	h
-9.05443897854698	0.61832103608310	4.09126928474600	h
-5.84035926358716	3.45358183233253	6.04243179317236	h

-1.45170457895253	3.38764875261640	4.61596068459054	h
1.90718786431173	3.57610497784748	2.91764983178912	h
2.82972861419214	0.62286894309554	4.19625659953836	h
4.96896585710739	3.11500723895964	-0.67636432596681	h
6.68307577204769	1.32912502591178	1.76750732236772	h
7.32590166083925	-3.48679791014804	-3.23280902980168	f
0.22634192996869	-1.04569186830761	-8.04427269673336	f

33

energy: -385.6488881805

0.32307964500531	-0.82832762813204	-1.42980000055753	c
0.20135210082001	-0.05432425702736	1.09614887674525	c
2.42791288745782	0.50894491254422	2.41410927476445	c
4.75313359059158	0.29757173477488	1.21051863820594	c
4.89667938321296	-0.47514127147821	-1.31229098700745	c
2.67686727379602	-1.03099648422274	-2.61096681135577	c
-1.99431820878899	0.19255392306409	2.45662459537861	o
-4.33265711839099	-0.37672921894615	1.18390048524048	c
-5.04400271319202	1.56212798902507	-0.71768891387277	c
2.28859220373036	1.10322612143233	4.37279873026306	h
6.46740911774731	0.73840889849621	2.25119273591288	h
6.71533643288099	-0.63595177368940	-2.24778816169231	h
2.75536782282790	-1.62762011450312	-4.57424935850636	h
-1.38125598000634	-1.24636488672946	-2.48625435871344	h
-5.70754302902457	-0.46045169338019	2.74206345938640	h
-4.24236360452306	-2.27812647306078	0.34617116295944	h

-6.33447640197601	1.09936187410273	-2.24404814206993	h
-4.46911340216825	3.51183834772991	-0.45044122508111	h

35

energy: -385.6622674850

0.22061895339981	0.47731200656894	-3.34822902949162	c
0.71075400017685	-0.85381420556253	-0.78939400687802	c
-1.64848543898264	-0.16097681019487	0.61719203640808	c
-3.62184772829730	0.19221928582905	-1.00826591591525	o
-2.63976359367295	0.10774912641767	-3.59360131904361	c
-1.76340564029349	0.21078075602147	3.16705741111082	c
0.53210804273202	0.19657200696501	4.55213450041897	c
2.88358242099048	0.06147093249797	3.27170336173459	c
3.04677243229275	-0.30487324955737	0.71344901575374	c
-3.55059427438233	0.64076302767244	4.07880235382546	h
0.49036133499690	0.46391389962726	6.58435365550720	h
4.60943062137912	0.29866887316378	4.36166727217895	h
4.86709092880401	-0.38515460543117	-0.23362534139678	h
0.63609920842123	-2.91707791566470	-1.19327821594215	h
-3.61379084702361	1.58893087179353	-4.64871258340798	h
-3.11595040238206	-1.74217304547355	-4.39789354579327	h
1.26119947458956	-0.36014083546552	-4.92373256092613	h
0.69582050725170	2.48582988079241	-3.20962708814313	h

TS-33-35

energy: -385.6320057359

-0.48363512686760	-0.74216109362834	-3.37703295368137	c
-1.15230522623301	-0.87380702289575	-0.85025063209580	c
0.70443968444182	-0.91250554007838	1.11464822914158	c
3.28789194165948	-0.73978514214213	0.38265435577674	c
3.94690877787629	-0.52501945902231	-2.14567969661090	c
2.07986551694728	-0.59614924785242	-4.02824046439423	c
-3.61755169541065	-0.60849763404515	-0.13422822073655	o
-3.75399226311776	0.56456630488656	2.39129116267202	c
-1.39323157308032	2.04968295966396	2.85850076863261	c
-5.47991644298621	1.70369444066105	2.32177215425752	h
-4.00015541285735	-0.93134285938139	3.80574852438519	h
-0.68024317118732	2.16522105655742	4.77972596161031	h
-1.06109165056882	3.70278028305570	1.68486639669682	h
4.74338794838540	-0.83011154313661	1.82759792978352	h
0.27718763368761	-1.91802990432230	2.85735159458354	h
5.92397245830949	-0.38048403023964	-2.67820018150772	h
2.61318375468467	-0.48162131068120	-6.00656479207395	h
-1.95471515368240	-0.64643025739902	-4.80396013643877	h

34

energy: -349.7332293289

1.01730926077242	-2.37997480764913	-1.24940149004748	c
0.39212171454229	-0.76083335989798	0.75121359819885	c
2.11917431138445	1.12676942720483	1.42226626484276	c
4.40334910452685	1.39857641170042	0.13206247730809	c
4.99858451119381	-0.22333868174714	-1.85771418590122	c

3.29606167690697	-2.11878139709490	-2.54345928363528	c
-2.11441928802754	-1.01217761888244	2.09198362583287	c
-4.35380008078219	0.04625405120547	0.52167542504392	c
-4.13520145994097	2.79120243525799	-0.05325120515997	c
1.66468667349111	2.39494994893027	2.97466653367179	h
5.71863926305579	2.87548151349263	0.68496198334568	h
6.77714411140982	-0.01932592478900	-2.86226003891125	h
3.74879082049004	-3.39893434379974	-4.08402268954248	h
-0.29569324060043	-3.86671683829125	-1.79200918924839	h
-6.12700898245989	-0.33877663443900	1.52520520637574	h
-4.45182985640623	-1.05551045724033	-1.25141683001756	h
-5.80597507484547	3.96822623552160	-0.22968722642381	h
-2.32431292052184	3.59942384945287	-0.58284082961439	h
-2.49090108421403	-3.00996999997821	2.49642178987406	h
-2.03671945997482	-0.01654380895681	3.90560606400788	h

36

energy: -349.7394233266

0.68400340984826	0.56107773715425	-3.16537536526251	c
1.13437208735168	-0.87502122319697	-0.64832750065606	c
-1.24261096342286	-0.28465272929906	0.80352067782561	c
-3.41033967843015	0.04182927470008	-1.02540513278082	c
-2.13799937520700	0.03834391434607	-3.67492357022668	c
-1.20539479805128	0.17339975707202	3.34529979279028	c
1.11083106731211	0.23242758956782	4.68823802504934	c
3.43164233691982	0.03974740893895	3.35686101588952	c

3.50701826590368	-0.40088242956172	0.81122509964039	c
-2.95332510296388	0.60810106156150	4.34033053079354	h
1.11739208463869	0.57787218619749	6.71008424301622	h
5.18953119570955	0.28501264453754	4.39340107250113	h
5.30500693829742	-0.52879892245473	-0.17639004106850	h
1.09104454603414	-2.91413951897584	-1.17094505925065	h
-2.98912178135793	1.41288817952643	-4.96192984977628	h
-2.33697194905914	-1.83035350777779	-4.54598004920844	h
1.91738914679187	-0.08102693040856	-4.69667259937954	h
1.01713259695045	2.58355910384628	-2.86345914838612	h
-4.82693679411953	-1.46490606385029	-0.85827037416061	h
-4.40266323314591	1.82552246807676	-0.66128176734968	h

TS-34-36

energy : -349.7125969513

0.06182946238538	-0.50899855047265	-3.34066887084775	c
-0.73261906255823	-0.96701452054976	-0.89417001003242	c
1.09390092571122	-1.06982502681238	1.10919039691850	c
3.70281239269937	-0.86904171051925	0.49215961016328	c
4.44537949946529	-0.40199115898172	-1.98069884124761	c
2.64430749076361	-0.24749836250574	-3.91383799397050	c
-3.43015853946839	-0.73514133409802	-0.01266617375892	c
-3.34894530072793	0.87072091876985	2.48284140125483	c
-0.77336847738844	2.06515009860693	2.76963491770908	c
-4.85452075650954	2.29842017234049	2.45647435415058	h
-3.72558975729928	-0.34863526792421	4.11227874535306	h

0.00265276161078	2.29197529434829	4.65982323049044	h
-0.25703363082278	3.55321993097240	1.44900374166150	h
5.11123164000062	-1.06807769507783	1.97290600169540	h
0.59687708834048	-2.06504105899790	2.84128784847046	h
6.44009144241363	-0.20298156918640	-2.42784909906668	h
3.23469573148107	0.09626903542775	-5.84909628083328	h
-1.33855720407268	-0.28596009761187	-4.83004506675503	h
-4.58430282894288	0.17746550361217	-1.46633020614593	h
-4.28868287708151	-2.58301460134012	0.36976229479092	h

S1

energy: -636.3205788765

2.25881949143897	-1.86307314171548	2.94677336873524	c
0.33692729317924	-0.44148634376053	4.13758790895220	c
0.85720965395774	0.56991579682916	6.55146158281786	c
3.16721673786906	0.12320603721460	7.72826075777952	c
5.04060263034577	-1.31090913601368	6.55320082955452	c
4.55670236655814	-2.28772840088241	4.15226451685332	c
-1.99804591709530	-0.07149045037308	2.99016280470392	n
-2.30406942380069	-0.73548769702898	0.33475380105644	c
-0.81722326953745	0.90902084751479	-1.43956823127061	c
-3.73692238623692	1.78096388405901	4.04289107812119	c
-0.54790565490455	1.70444064550611	7.51823190732423	h
3.50131947283376	0.92354535461440	9.59026178756346	h
6.84036484578010	-1.64578322505126	7.47832549385771	h
5.99252541387284	-3.39076066130402	3.18253082486477	h

1.97196023559800	-2.62368240434129	1.06680639759198	h
-5.44308864465993	1.80823426344926	2.88348627674472	h
-4.27925271205672	1.25889258930548	5.97199950773392	h
-2.93573960154594	3.70154580870206	4.08902255014563	h
-4.33379119601210	-0.62532046488194	-0.09198562185205	h
-1.79982326789588	-2.73874082314275	0.08007218041038	h
-0.73736376125248	0.57859987113891	-4.08610167572578	c
0.27413397221021	2.43452535189965	-0.60033408048260	h
0.73776047212396	2.22831318915491	-5.61968141788676	c
0.84754695534651	1.93124022922518	-8.21641001651648	c
-0.50240123571086	-0.01637271668460	-9.40178040411183	c
-1.96702221726593	-1.66384497540932	-7.93802262313551	c
-2.09221301541947	-1.38452185297609	-5.33553862330617	c
1.79139219626923	3.73744484820221	-4.70628468421418	h
1.98930318151840	3.21470335863592	-9.34150035021129	h
-0.41242573912854	-0.24626161322596	-11.43883893842135	h
-3.01660235518575	-3.17660539928282	-8.84765076622003	h
-3.23989452119310	-2.68252276937732	-4.23439614145630	h

S5

energy: -636.3178197078

1.16003304675342	-3.32106320185702	-3.12124429700032	c
1.06155355022479	-0.67345593703302	-3.03177426538827	c
2.35935532770498	0.69329858728663	-4.88754271684327	c
3.71492819183804	-0.53754041777766	-6.78407602798523	c
3.79289726373110	-3.17099264086520	-6.85449506484006	c

2.51052386535316	-4.55833876741544	-5.01317568931384	c
-0.35159560541495	0.68902316654260	-0.97623870787453	c
0.59515369499872	0.16009755810644	1.76434063267724	c
-1.64277613873328	0.93779039693951	3.33949620728683	c
-3.76640824611188	0.75201461490858	1.87161078210436	n
-3.17263508986495	-0.01871698421746	-0.71509985493559	c
-1.39459008230213	1.74940975519007	5.79996360707662	c
1.05816375063743	2.28755594912267	6.72697598163146	c
3.18203303295724	2.19360940070116	5.09411282034461	c
3.01039472856824	1.32089065491156	2.66014544900992	c
-6.32087158769421	1.04389798914150	2.78775754625484	c
-3.04068927477422	2.10917408931659	6.97310901284443	h
1.28684782580301	2.94082136943178	8.65736619627673	h
4.99223091033247	2.86856494403251	5.79533789507688	h
4.64909344534005	1.25571729968838	1.42466933062850	h
0.75869958769444	-1.93291398350392	1.89215188260973	h
-7.55345618071622	1.57038135307386	1.21422877133793	h
-7.05518715538953	-0.70018069206049	3.65068472524004	h
-6.38096597776095	2.55104031688511	4.20494582725614	h
-4.38147584247876	0.98797552658770	-2.06126827990910	h
-3.45450240621180	-2.06218620321196	-1.00018109997445	h
-0.22864474429703	2.73135476623874	-1.30212553246479	h
2.30726072555180	2.74741169643784	-4.83483684411966	h
4.71072268178574	0.56138870474535	-8.20416236206229	h
4.84577709255913	-4.13638882258825	-8.32859237002750	h
2.56524167784800	-6.61070631434058	-5.04918695922808	h

0.18288793206816	-4.42893417441761	-1.69289659568940	h

S-TS-1-5

energy: -636.2991524921

-1.15117688309950	1.68191389344087	3.98580718171381	c
-0.99823059749910	2.72042613377233	1.57425785931107	c
1.42767813515717	3.27956926256378	0.43823514088699	c
3.64562962185933	2.60413939734613	1.82450520083032	c
3.45645738718501	1.45612645405620	4.16522400771315	c
1.07531918362856	1.06647691942622	5.28243867255037	c
-2.97155083721293	2.99509086124943	-0.09983475886460	n
-5.55337549140693	2.43804573575012	0.62305416516365	c
-2.18656509389834	2.52090826126922	-2.75946135959270	c
0.45776981895925	1.40443049371659	-2.80182060206279	c
-6.00044541475590	3.39567600836788	2.39853189479232	h
-6.83268381999833	3.14701226636378	-0.83526212432398	h
-5.89907125830229	0.39566843308640	0.87087377494694	h
-3.56791611769401	1.22629689670354	-3.61149156416173	h
-2.22973463526987	4.28816306548131	-3.84541955126328	h
1.75249155520092	2.27195440348360	-4.14314565446870	h
0.83995962790836	-1.28561531193286	-2.42094725914393	c
5.48795846182099	3.04752317731463	1.03372347796437	h
1.53501237352364	4.99037390053584	-0.70508255656226	h
5.15554241450636	0.93249356580512	5.19095309297849	h
0.94912743832333	0.21640070931438	7.14603789032259	h
-2.97547509079658	1.23647476188022	4.81279699540725	h

3.16264077172673	-2.40609838661305	-3.10157477659852	c
3.63267595308913	-4.94820535789763	-2.64671827828403	c
1.79928630824248	-6.45444051284073	-1.48304373182527	c
-0.50637174629737	-5.37783073073749	-0.78546792573290	c
-0.98178652349022	-2.83225412109744	-1.24027857977964	c
4.60041194487166	-1.24242128140116	-3.99827367793231	h
5.43398792589490	-5.76598018125967	-3.19825899052022	h
2.16539191452053	-8.44206219629077	-1.12563706747457	h
-1.94159816302697	-6.53024524893931	0.12574279051928	h
-2.78135916366995	-2.03001127191750	-0.67046368650946	h

S2

energy: -444.4490093097

3.32826235098078	1.52419247902674	-3.73743914825030	c
1.06557112704849	1.43068612851331	-2.40386292487687	c
1.04591294618424	1.02571838196494	0.23715357805731	c
3.40472352628133	0.66674553411475	1.44073680354213	c
5.64981815185467	0.76310772878692	0.07349084822855	c
5.64814104689044	1.19954518454273	-2.52516731705920	c
-1.17275565082496	0.98863688261507	1.63246500920168	n
-1.11429227376530	0.15394651817175	4.25142259030666	c
-3.65849370980344	1.11931721150889	0.43728621908338	c
-4.77307415132704	-1.39786230394227	-0.24719026536519	c
-3.23253194004712	-3.52572429237905	-1.26265892258990	c
3.48642189211893	0.32126945819261	3.45790722915420	h
7.42539483131319	0.48582520292598	1.06860923446709	h

7.40450591876241	1.26701873747851	-3.58206700111493	h
3.26491573697796	1.83901803179050	-5.76637674065945	h
-0.69069310937181	1.64556576944056	-3.43560678150698	h
-3.04574574179517	0.15203885142419	4.97473619929458	h
-0.34134583598318	-1.76568388716549	4.45123619219783	h
0.02202171874167	1.43744659123916	5.41830990610936	h
-3.50612745319700	2.37420755515313	-1.21572044129157	h
-4.93340925793130	2.10093316162620	1.74756582073633	h
-4.09730480340420	-5.35627149637106	-0.82622045065527	h
-3.04550732839707	-3.43163543213994	-3.33934285726882	h
-1.30895169324838	-3.50209023319773	-0.50150240196284	h
-6.82545629805821	-1.51595176332041	-0.34776437777798	h

S6

energy: -444.4636431026

0.57438828674637	0.18704617495303	-4.06689531377848	c
0.67299937971274	0.87363895240150	-1.32449852430993	c
-1.72999047560572	0.21999091715808	0.05605111052492	c
-3.97907990096102	-0.25070450318985	-1.16007317678991	c
-3.99548263773954	-0.39956058870490	-3.83344532178590	c
-1.70099509691519	-0.29027349799599	-5.21180428968306	c
-1.18958605055597	0.13890865072994	2.58805113658243	n
1.54893915775296	0.03559173015853	3.04909271029957	c
2.70780649033692	-0.35485689802308	0.40011947994127	c
5.36359392327920	0.72069130566382	0.10549576196864	c
-2.99667084221595	-0.60510334558099	4.49894540807984	c

-5.69306381900001	-0.63846825478173	-0.09762258780935	h
-5.75754758241481	-0.76824866564849	-4.81558423387552	h
-1.75325712842470	-0.63275910836781	-7.23775371708387	h
2.31176270369072	0.25848487996804	-5.16056911398074	h
0.96470668123184	2.95017525927375	-1.14682972750291	h
-2.29256936168103	-0.04855147052674	6.35953747535670	h
-4.79740337661830	0.35681546521859	4.17023876288487	h
-3.33830511370431	-2.65958925296044	4.49792100090756	h
2.00671382056634	-1.51480220026108	4.34740392890014	h
2.21425257678176	1.80924285375876	3.91130378565076	h
6.68827638345772	-0.15878409496187	1.43244382500667	h
6.08182874174279	0.39609027048505	-1.80770061372239	h
5.36587826392106	2.76354675047384	0.45391339599124	h
2.72280497661593	-2.38852132923996	-0.00774116177267	h

S-TS-2-6

energy: -444.4346589320

-3.39943636032135	-0.25540327990052	3.78431523888140	c
-3.31011800668923	0.22902581665849	1.17988552572831	c
-1.11354705479031	1.20951543900913	0.10373008965120	c
1.02989965444255	1.81415476492863	1.67125176545376	c
0.86819846210884	1.28799110555056	4.29532480703872	c
-1.29052680465048	0.19987978729691	5.32119430421238	c
-0.63920427903575	1.38325881244879	-2.45797789792657	n
2.07041896550569	0.92067384851415	-3.06997970166237	c
3.33033033882288	-0.38300083481379	-0.86679729230072	c

2.72408874787147	-3.10649938500713	-0.38580133167558	c
-2.53448408994521	0.59792701423122	-4.27679033966410	c
-4.31001915012700	1.59257023241321	-3.91318151468563	h
-1.89484622026519	1.10119669105805	-6.17437608305988	h
-2.90324209785550	-1.45439749457659	-4.21787889002374	h
2.12790838425161	-0.24445986324058	-4.79064803019378	h
3.02670878175434	2.71172975604416	-3.50245068533403	h
2.44200068685520	1.79940874100083	5.51178744333930	h
2.25468573223942	3.35612908131688	1.08028813861692	h
-1.38062979910604	-0.20938022029430	7.33062502140386	h
-5.11236724503961	-1.03975078913802	4.59943360297275	h
-4.91881136637841	-0.24868207368749	-0.00026710482290	h
3.44360256895270	-3.71823827308143	1.45425575764014	h
0.67698243870454	-3.42609959465134	-0.40477431337212	h
3.56452788754003	-4.34261378330865	-1.83671477076719	h
5.24787982515512	0.22506450122863	-0.43445373945032	h

S3

energy: -633.1306074599

1.74494322257048	-1.96407479353740	1.40002976525349	c
-0.09434807067397	-0.51102632883530	2.67165153776288	c
0.65056583001679	0.81999122501446	4.85791156054503	c
3.11559017213224	0.64037852500468	5.76496073855757	c
4.91334209227053	-0.82634560330142	4.52082189295131	c
4.19778663762066	-2.10951000812276	2.32783284295059	c
-2.60122033588436	-0.43217984690723	1.84919254223224	n

-3.32803846071037	-1.37200472730939	-0.67196581892841	c
-2.58951914734813	0.48425231823940	-2.65630816217766	c
-4.37668576609312	1.25546886352927	3.09973869320228	c
-0.68838921852107	2.00088378564022	5.86131030389841	h
3.62591091805477	1.67934180538672	7.46143454732624	h
6.83459211228263	-0.95228256653149	5.22852681342204	h
5.57208822643493	-3.23931466630807	1.30224955009935	h
1.28742599452976	-2.93908880670354	-0.34204031098413	h
-6.24500701939706	0.96773648808786	2.27271522675511	h
-4.48426565945874	0.80955699995324	5.11882841161644	h
-3.86398098082147	3.26082630389613	2.90391075331007	h
-5.37655448178760	-1.67178751488777	-0.62832931777301	h
-2.43073607452451	-3.19554617230380	-1.02891744626428	h
-0.67839129605630	-0.10470604731942	-4.50204474250197	c
-3.37640558469765	2.37927516373133	-2.62397605920274	h
-0.20990169298096	1.89699194628882	-6.07652618710496	o
0.40901933845072	-2.14927955347377	-4.71492176936421	o
1.66184158586054	1.39399699686890	-7.99722514806732	c
1.81711777544478	3.14392156265883	-9.07217427215405	h
3.46643288286411	0.89969896473680	-7.12120368286455	h
1.04678700042245	-0.16517431349538	-9.20548226249568	h

S7

energy: -633.1339612983

-0.10086583325261	1.85315275352747	-2.11832087544621	c
0.99534292874519	2.16163735873780	0.63683681439109	c

-1.08734870232910	1.00872893926469	2.19503905525610	c
-3.33689681145151	1.47126478576408	1.00071272632690	n
-2.95229389894813	2.20121753623085	-1.65957317282979	c
-0.63406041669996	-0.32639008998565	4.38076312626153	c
1.88857821575616	-0.85731473826133	5.08123330140529	c
3.91546060644451	-0.19853634665910	3.45729883873026	c
3.55369573827795	1.13938588533698	1.27058911535191	c
-5.68953340662348	0.29725913281974	1.76920558653622	c
-2.18817719344021	-1.07771834941037	5.49123132726536	h
2.26109676261066	-1.91513201772493	6.79776856772061	h
5.81553952910411	-0.80040509105802	3.95781770285927	h
5.12674380856283	1.62118877644749	0.04469528837370	h
0.98031712222870	4.23452584080137	0.95351176977099	h
-7.26455536747590	1.25535541072998	0.83765115323342	h
-5.73237348259375	-1.72196874830862	1.27373435089796	h
-5.92787821688347	0.49343600561841	3.81239810078079	h
-3.52543302217039	4.17172944072474	-1.96898648989843	h
-4.07082614215253	0.97357089840027	-2.89333024120742	h
0.46965368984872	-0.76612742324662	-3.10792755986943	c
0.69625020153678	3.26437341243528	-3.39518952936072	h
2.76100059149627	-0.82803633495875	-4.25363263144696	o
-0.87839490190818	-2.60888391998128	-2.85994613230889	o
3.57278165097749	-3.32498816003513	-5.03343594810397	c
5.41640401378334	-3.04281298266543	-5.90519136109869	h
3.71025942449474	-4.55877178604977	-3.38430706836249	h
2.22551311206185	-4.11974018849399	-6.38064581522833	h

S-TS-3-7

energy: -633.1164156790

-1.01053107253422	1.14582086672530	3.76309011021649	c
-0.94991046165010	2.08062122840687	1.29444153793523	c
1.44911135891007	2.60633568014016	0.08507749559236	c
3.71127806508394	1.98749855842324	1.41285120276634	c
3.60349698549248	0.92890673557784	3.79774385433005	c
1.25121761202656	0.57973799617726	4.99663730486373	c
-2.98308294587273	2.27529648691483	-0.29389307783709	n
-5.48199655394626	1.46143087111451	0.51178770605810	c
-2.29157399798844	1.86102559612416	-2.98911891475491	c
0.28782084587227	0.63576800447287	-3.13280154811783	c
-5.96138707846568	2.35437481655644	2.31188547107381	h
-6.86733506746131	2.06950996919616	-0.89293188489519	h
-5.57242812789296	-0.60506055811311	0.71336898163228	h
-3.74583159520290	0.64107738929493	-3.81693031755798	h
-2.28466880395736	3.66133395806989	-4.01948515207964	h
1.62122031829031	1.30483181266723	-4.54032697055672	h
0.42819514706196	-2.02498724412761	-2.46330947559144	c
5.52024272005677	2.40734338712147	0.53903948834506	h
1.52827286491255	4.22994209735046	-1.17657706561186	h
5.33350599427360	0.44909474487036	4.79189144298946	h
1.18482541350705	-0.20488305214326	6.89217773119096	h
-2.80092306732171	0.72758342957936	4.66897274471711	h
2.75004931843661	-3.03483850113753	-2.99367367626323	o
-1.26249599850500	-3.23228055214046	-1.43293423596832	o

3.06292183315187	-5.61135706785677	-2.15351416128263	c
4.98416467674077	-6.12574736913800	-2.69030650500033	h
2.81172501161601	-5.72637687400620	-0.10618893116056	h
1.68411660536599	-6.84200241011979	-3.07697315503223	h

S8

energy: -444.4457657694

1.55167582101548	-0.90884489665831	-1.22546519953652	c
-0.78525651069030	-0.46269776983797	-0.02294405832935	c
-0.71971592680725	0.12788100874988	2.57903549886259	c
1.55331850524156	0.19259731096810	3.89536798894070	c
3.87172069115112	-0.29276690386018	2.71716650652927	c
3.80410859121631	-0.83261699901948	0.13254589210714	c
-3.07230372055638	-0.62890581426526	-1.31635324998590	n
-3.06394701670894	-0.72269552000500	-4.07903790727370	c
-2.44827819790611	1.74219698199163	-5.31092113760706	c
-5.34188218965905	0.38877091110585	-0.13636338618873	c
-2.45599745797081	0.53263470909874	3.58807388665211	h
1.51319414060685	0.64816397390675	5.90069562117276	h
6.32469873745163	-0.21990319867581	4.16986286365078	c
5.56547396429720	-1.19039467412550	-0.86675567918448	h
1.63168393481949	-1.29739956950890	-3.23496755366118	h
-6.92020240284378	0.14321299081070	-1.44245689256133	h
-5.78003957162043	-0.64550149113119	1.60287500648431	h
-5.17438079041472	2.41046961421635	0.32652141868370	h
-4.93708605195278	-1.37869455777863	-4.68106579886938	h

-1.74788219332429	-2.22720880306764	-4.66864475694740	h
-3.03715490032180	2.13033326656236	-7.23857259818281	h
-1.24300007284505	3.09704673818950	-4.35321167333689	h
7.93709929884097	-0.52913266368511	2.91258406333172	h
6.59485468338416	1.60564600687436	5.11324559896204	h
6.37929863559691	-1.68219065085531	5.63878554628766	h

S13

energy: -444.4644908327

0.45850247722549	-0.46326187022086	-3.90334073775033	c
1.05616084896635	0.95717577231955	-1.41306011465272	c
-1.34582248936180	0.54942475009833	0.05830349328891	c
-3.30681924670740	0.41413234663714	-1.63083478625104	n
-2.39965474172965	-0.00625627025002	-4.22963656971500	c
-1.37619633041432	0.31497042350071	2.64738750692895	c
0.94693429415255	0.17392471555970	3.96109811687317	c
3.29269725671887	0.01714181293894	2.64009397494649	c
3.36710452910383	0.25875152254987	0.06226080398999	c
-5.88281729371227	-0.14950756667622	-0.91400613146971	c
-3.14580800356556	0.11385408362782	3.67027809912152	h
0.93602462114146	-0.00818974499690	6.00599200340362	h
5.67967392889612	-0.46341937453136	4.13191187245369	c
5.15746030653336	0.13122460260936	-0.94171016706639	h
1.15112547763403	2.99729728892726	-1.90830821471688	h
-7.14928538957405	0.33794445490212	-2.47185788266928	h
-6.14496978348632	-2.16154758966773	-0.44218995961280	h

-6.41236296517276	0.98233404810842	0.73331603421932	h
-2.79305922187055	1.65758476573996	-5.41090602065723	h
-3.37119004863501	-1.63076978856690	-5.07511064552216	h
0.83201328540306	-2.48216419614653	-3.64423945726065	h
1.55746073080098	0.21357822462187	-5.51626448501057	h
7.32141804914692	-0.60820046166544	2.88376569816836	h
6.03147975396527	1.07184556586849	5.48021348183663	h
5.53992995454148	-2.21786751528761	5.22684408712391	h

S-TS-8-13

energy: -444.4305313310

-1.28537323650201	-0.04463671742232	2.50525436459891	c
-1.17543556790845	0.82410180708501	0.01893704621685	c
1.23945272692775	1.32622046085414	-1.11882577091958	c
3.45196592710951	0.83854968384368	0.30567405115705	c
3.34254385159977	-0.14177122557238	2.74480273377144	c
0.94931864838263	-0.50632320232703	3.84371409961637	c
-3.20677126575762	0.98846923287376	-1.61727758692965	n
-5.72103968832121	0.29621109472286	-0.77014856100293	c
-2.48919965439885	0.35587955475813	-4.26796476121241	c
0.00355170557372	-1.01242210019322	-4.26710317135059	c
-6.20193810192958	1.35846248745776	0.93680830118497	h
-7.08573121817692	0.79359844040495	-2.23800702704424	h
-5.90036001686015	-1.74006248587483	-0.35266950969922	h
-4.02169251419981	-0.79813239069421	-5.06467337952691	h
-2.36777019778875	2.08517615141879	-5.41163722997649	h

1.31407611213209	-0.66896358617738	-5.80968526797026	h
0.05127281701890	-2.90185043833305	-3.46192689856031	h
5.27888141312273	1.29584134990393	-0.51889135425474	h
1.34624258680109	2.76867493000357	-2.57850051499522	h
5.71941058653009	-0.74246890051728	4.20399486355872	c
0.82366900195691	-1.20099548498236	5.77415333055630	h
-3.09381680064893	-0.44896622055445	3.38533230107932	h
5.58636915401301	-0.09422268801076	6.16594672881028	h
6.07300778215474	-2.78632601757802	4.25356545899459	h
7.36936594916963	0.15595626490998	3.33912775389825	h

S9

energy: -519.6940752307

1.00250912336779	-0.82015610033220	-1.14612547164322	c
-1.30865262637485	-0.44267898120332	0.11406465658300	c
-1.18754184283933	0.16874662255573	2.71043437448561	c
1.10637424753810	0.31951025909904	3.97314175725277	c
3.38209602008712	-0.10572359581342	2.70065732378507	c
3.31012058771701	-0.66791309581280	0.12420360961378	c
-3.63525680408888	-0.70495529608880	-1.11362995873325	n
-3.68555149526698	-0.73271705987706	-3.87897313180113	c
-3.16327987308330	1.77609151730162	-5.06463454686957	c
-5.88371318480930	0.30084560162061	0.11747875082907	c
-2.90498274864818	0.52327913144919	3.76849866252611	h
1.16016396345105	0.78323126429262	5.97143447409494	h
5.55524850866365	0.09435845719246	4.12005714763060	o

5.03516383402418	-0.97682974710561	-0.93816975593399	h
1.03944564383896	-1.22482862553430	-3.15337577073338	h
-7.49840873447630	-0.00243350848554	-1.13127804414169	h
-6.24703453344831	-0.70634150759153	1.88911773596668	h
-5.74291148677402	2.33544016437508	0.53536234657220	h
-5.55424820543789	-1.42392187728936	-4.45442711833899	h
-2.34540755055447	-2.18969558950553	-4.53077674245331	h
-3.84617852027110	2.21546812992613	-6.94965052297857	h
-1.95528093283273	3.13116384971021	-4.11041037809116	h
7.88315321663180	-0.33481223977659	2.83403399433403	c
9.35721236243633	-0.10967135096220	4.25740107868359	h
7.96118200162406	-2.25274336664827	2.04421051014625	h
8.16577902952550	1.04728694450383	1.31135501921461	h

S14

energy: -519.7149889697

-0.15096091420214	-0.34548674440178	-3.85335126486909	c
0.46758565975266	0.89263060184085	-1.28079735231057	c
-1.86418504244713	0.29728247445364	0.24116831716901	c
-3.82525484155008	-0.14491139500587	-1.40081865941625	n
-3.01661324888413	0.09459436721668	-4.03832067221606	c
-1.81551927897538	0.17818436313559	2.84152908261928	c
0.54343284531828	0.18423938370579	4.08195603778295	c
2.83005356229142	0.04838363110384	2.65217014979975	c
2.86615092729416	0.22103231863582	0.06937416768916	c
-6.45351449888737	-0.23661974227552	-0.67290216241569	c

-3.53983967079827	-0.03813792072546	3.93478695185058	h
0.66888382278995	0.08559822649936	6.12436055893043	h
4.94515654597745	-0.31527638259542	4.13185710904703	o
4.60600833597883	0.14366528206470	-1.00935591982685	h
0.43603056850853	2.96828281049760	-1.63021021817785	h
-7.53144329806247	-1.23310313153429	-2.12823525623149	h
-6.64286938112346	-1.27236742503546	1.10789291551829	h
-7.27285893963570	1.66050823996899	-0.41981889179462	h
-3.43992860493748	1.99106077208945	-4.79590651070685	h
-3.99996473990248	-1.29931245377803	-5.21155421938211	h
0.25395927450649	-2.37343575790816	-3.77268167157695	h
0.88576665892087	0.49001179065159	-5.43250203995736	h
7.30168343445387	-0.46723440399165	2.84061871436553	c
8.73046005702682	-0.72259367315497	4.30521506282124	h
7.33450289609032	-2.08215627716676	1.53714594694530	h
7.68327787049634	1.27516104570920	1.77837982434328	h

S-TS-9-14

energy: -519.6787731998

-1.43313005454231	0.21995488180629	1.92070460931108	c
-1.26294418170776	0.97157875643246	-0.59114569512616	c
1.18683725584370	1.38036941565483	-1.70222391119152	c
3.36536812727644	0.95345940509698	-0.22122336424461	c
3.15410598645062	0.09203204212216	2.25779301443156	c
0.76040231346073	-0.20706031511294	3.35695003580307	c
-3.26179544411280	1.08735337482087	-2.28514876896083	n

-5.78891909974695	0.43192353212957	-1.44935258049635	c
-2.48624228615967	0.27230082672223	-4.86795168986714	c
-0.01270821633163	-1.12606807679043	-4.70780037431820	c
-6.31024073801618	1.59347209856414	0.17990592371599	h
-7.12593743022538	0.83127497568476	-2.97148351516839	h
-5.96881751781190	-1.57689402352643	-0.91055642191277	h
-4.01133489256269	-0.91037594514813	-5.63693377403379	h
-2.30430620950877	1.92185453968298	-6.11652446586205	h
1.34152529447054	-0.90681901242090	-6.23574084850821	h
-0.01557354856294	-2.96010004734261	-3.78165473349636	h
5.23052834277007	1.35233913692073	-0.97666179967803	h
1.34342795494672	2.73882129859424	-3.23579723553563	h
5.39695200448586	-0.32352308960963	3.51766101637276	o
0.56430850884245	-0.80976921106468	5.30462588022980	h
-3.26187306129491	-0.13443671674260	2.77900402753797	h
5.22847397509042	-1.07777071364303	6.09806810242447	c
7.17491759777008	-1.28498589369837	6.74514588138265	h
4.26254615049523	0.36067969051604	7.24096188647345	h
4.23442916868093	-2.88961092964851	6.28937880071714	h

S10

energy: -504.3939581466

0.73388136475865	-0.97429761659053	-2.46344577773253	c
0.61416176445675	-0.49028018712097	0.15924939145081	c
2.89416407769093	0.11685865301563	1.40564667400811	c
5.18768967570177	0.16606562154879	0.11416030218951	c

5.22766131863285	-0.36538732554993	-2.45057475261420	c
3.02502649255530	-0.92478165873479	-3.75628121348154	c
-1.65360703642554	-0.64234983420725	1.49081337817861	n
-4.03764893231388	-0.72835559400663	0.09532398720553	c
-4.76227671142849	1.72120198073784	-1.10752251676357	c
-1.76527003288012	0.40147740638837	4.03590603339320	c
2.89466014322803	0.54762954304845	3.40707355813296	h
6.93058885225085	0.62584214446521	1.09212120282532	h
3.09715744957191	-1.30111823668928	-5.77085166096319	h
-0.96871956844565	-1.37775638821959	-3.52599438046221	h
-3.68533517971579	0.16492499719238	4.75222803869103	h
-0.47993271131907	-0.62236969451228	5.29543318739449	h
-1.27847412665837	2.42369540081564	4.10197222144323	h
-5.50781932634916	-1.33943389548190	1.42489736467917	h
-3.91292849903405	-2.26532487503531	-1.30757822360578	h
-6.70567674336369	2.08379966524776	-1.65954930904394	h
-3.31994253037824	3.08889173441466	-1.61152287400324	h
7.47264025946510	-0.30893184072627	-3.72150463092169	f

S15

energy: -504.4144864279

2.11057214623033	0.50285266042483	-2.85431312514292	c
-0.38514510627270	-0.81287626322844	-2.07596066210486	c
-0.35763157625639	-0.44015596131215	0.75280765927045	c
2.11086587561638	-0.42445387693441	1.52631512771862	n
3.86505176649026	-0.09728453550089	-0.60966928012753	c

-2.52184554337180	-0.11923673857793	2.16274661741809	c
-4.86223475084929	0.15458459432931	0.90063310400457	c
-4.87771872826702	0.31056118494584	-1.76693698875684	c
-2.81424841002029	0.01141412433800	-3.27241817135462	c
2.89113062658918	0.01099560485496	4.10966318996469	c
-2.44082813364186	0.05229976193990	4.20633024906563	h
-6.61140990703240	0.41828131819942	1.93168978349669	h
-2.97092663862313	0.15449010765849	-5.31162126740146	h
-0.10506030971747	-2.86351889369968	-2.43326552356789	h
4.86579882222181	-0.55269641104544	4.33017116431412	h
2.69925782973972	2.01055823315313	4.65733463788321	h
1.74038274565015	-1.13293169704317	5.39172304855305	h
4.95862313815019	-1.83677764293649	-0.92165996078914	h
5.20234352265210	1.43491311855569	-0.21152316244542	h
1.79587486119692	2.54349933474213	-2.98864082501814	h
2.86444149830644	-0.17550523501743	-4.65346059429934	h
-7.15729372879113	0.85098721215434	-2.86994502068093	f

S-TS-10-15

energy: -504.3793652364

0.61955013574183	-0.66015829172505	-3.03657956623637	c
-0.10423449114398	-0.84487975778270	-0.50671792935882	c
1.78863259023868	-0.95798432867247	1.44162985757401	c
4.36909128002799	-0.80734276029687	0.75025997613436	c
4.99161727962616	-0.51391533348153	-1.76768472177693	c
3.18298403601342	-0.50290501799003	-3.68254413645553	c

-2.55823334372811	-0.63809312358968	0.37090529036647	n
-4.62875902883345	-0.28334623648073	-1.39176391174440	c
-2.69438680801474	0.68271590689754	2.85332443215718	c
-0.27276392403179	2.10632425193783	3.29619945053259	c
-4.60367297025802	-1.78383086646047	-2.81363043245557	h
-6.41456575053069	-0.40906365629317	-0.36339768207488	h
-4.54888735976465	1.55705640754853	-2.37103060882968	h
-4.34593924474428	1.94088106248414	2.79845045215481	h
-3.01960149351695	-0.68660026366480	4.38055473260358	h
0.46271817889374	2.23284326447463	5.20860897016357	h
0.13108124612943	3.70785875486692	2.07483988542339	h
5.84849780316744	-0.98759510363994	2.15810065226171	h
1.33654318587117	-1.94429006625929	3.18490487492738	h
3.77284861019061	-0.32865357563893	-5.63777275556436	h
-0.79398173477509	-0.54660109366552	-4.51712714870505	h
7.48146180344139	-0.33242017256892	-2.42952968109787	f

S11

energy: -557.8473211435

3.33992533196004	0.04500907290744	0.70353248453945	c
3.46053552007996	-0.39477369506936	-1.90996219979508	c
1.17005854566956	-0.80174846362717	-3.19424842246567	c
-1.12421055048102	-0.78892465240255	-1.94828403356982	c
-1.24409948099873	-0.38159576149680	0.69969689623545	c
1.05736987724036	0.06484401995183	1.98918531116045	c
5.85834381218248	-0.42726945690578	-3.35058977981275	c

-3.50331310151834	-0.42759069092328	1.99145474786837	n
-3.60374830676515	0.40323360950490	4.61572381669315	c
-5.90339168572628	-0.68042577990315	0.64193754094963	c
-6.81301963813330	1.72900429253201	-0.51157592556177	c
1.05398021481996	0.41393871427679	4.00718348759186	h
5.06111303669993	0.38534972691449	1.76885177489257	h
1.24791996363194	-1.11437997596768	-5.22093231788964	h
-2.84108435559885	-1.06320503568564	-3.03089553691725	h
-5.56380120518538	0.32629379447047	5.24980442554465	h
-2.47441046630224	-0.84101050182459	5.82847777221321	h
-2.91382556884747	2.34835794161721	4.84337476116717	h
-7.30065343749444	-1.40722261244399	1.99042162041097	h
-5.68901595557960	-2.18465030746575	-0.78335237977459	h
-8.79628668497674	1.98058980895229	-0.97448711679198	h
-5.46842474333538	3.16249815919867	-1.09540344309569	h
8.31888250133229	0.03185360914700	-1.93214818752188	c
5.88750075071202	-0.81617070731262	-5.64150017524467	o
9.88149028594030	-0.06218274963331	-3.27254425443599	h
8.30782051918345	1.88918728774628	-1.01407690479763	h
8.59434482149069	-1.38900964655767	-0.44964396159261	h

S16

energy: -557.8632943657

3.94684245988341	0.04166313594065	-2.54812106095426	c
1.43941545628895	-1.07944404676480	-1.55001401579747	c
1.47142708145009	-0.29659575992966	1.19220585462000	c

3.91134202421974	0.13490271100092	1.88612726300053	n
5.67826563247849	-0.20472340292186	-0.22844664090129	c
-0.71589377924229	-0.08301680869095	2.59109102559839	c
-3.05952919122096	-0.13641069846291	1.30973607315239	c
-3.18053113326805	-0.16687144140834	-1.36821910283025	c
-1.00218279879663	-0.43402593307717	-2.80458146765747	c
-5.62786026608707	0.17742145053901	-2.72181925783286	c
-8.04493646526439	0.40291399886287	-1.17737003843897	c
4.74010303965747	0.63963134264952	4.44120973679056	c
-5.72055932400664	0.28983524659232	-5.03810506700717	o
-0.66135008570025	0.24983100996680	4.61548620901290	h
-4.78229632454591	0.02051972561548	2.40765278265537	h
-1.13810139974441	-0.39410621099602	-4.85132193598463	h
1.68490413786382	-3.17059657700592	-1.58284458127998	h
6.52162564616581	1.68536245334924	4.38613792916601	h
3.32443713355580	1.79433559882280	5.41071232730836	h
5.02725361222877	-1.10799067641047	5.52862547629808	h
6.59248755631424	-2.07323269754688	-0.14580464836018	h
7.15839621188987	1.23948640861285	-0.14428746204199	h
3.68107139645906	2.03699853281427	-3.02645222216957	h
4.68147530156823	-0.95373882727784	-4.20032554584078	h
-8.30316784710326	-1.24755431174924	0.04694011995743	h
-7.98230548211205	2.07469939994120	0.04451148290055	h
-9.64033259293189	0.56070637753435	-2.47272323336380	h

S-TS-11-16

energy: -557.8310316114

-1.77421922393125	-0.74762457754254	0.37921958314858	c
0.11812937032644	-0.77725303820319	2.35037492936517	c
2.68286093203736	-0.68293654380596	1.64058145396295	c
3.40474371187710	-0.54308630903040	-0.90392920227845	c
1.52128430232843	-0.67502178155468	-2.78116606240016	c
-1.04128797659210	-0.77517248768384	-2.15724817679329	c
6.13625685824109	-0.38868093574192	-1.52259542343284	c
7.74486801228623	-0.24083369781448	0.14628962598954	o
-4.19713858883594	-0.41640697394859	1.23310047208664	n
-4.31817501852217	0.97654542764143	3.67823339501940	c
-1.84805193409070	2.32205182755991	4.08501206384082	c
6.92740370098017	-0.41963445945847	-4.28832767459002	c
-6.31542430540616	-0.21957937619745	-0.49878705041380	c
-6.26452986447606	-1.78421797353931	-1.84699452262098	h
-8.07414889399120	-0.35197902827742	0.57474866122237	h
-6.31151320804163	1.57380297496536	-1.55875936023143	h
-5.92212014698339	2.28885763823310	3.55474275052195	h
-4.71239475827332	-0.33606916578495	5.23739378728178	h
-1.11773572834630	2.47700065472779	5.99647773028071	h
-1.41393907700118	3.89489417895606	2.83685552518922	h
4.16119619490771	-0.77633705253584	3.06005234606260	h
-0.34579034635163	-1.72261762657579	4.11656121314603	h
2.04654612551565	-0.64365990116437	-4.76366169748004	h
-2.45278778295246	-0.74967233704074	-3.64511657600900	h

6.07074280320786	1.15872796594171	-5.32144252422272	h
8.98061362623830	-0.27208677383973	-4.38837418238798	h
6.31461121585002	-2.16901062828698	-5.21324108425752	h

S12

energy: -421.5579719572

4.71166678086624	-0.81045422956885	0.72247176837423	c
2.51484910882914	-0.72743791074244	-0.62761196948508	c
0.16336083090811	-0.24972973699505	0.61705773949261	c
0.26776188792695	0.13962554813120	3.28863536603199	c
2.52324811471223	0.03154463455400	4.53931096053753	c
4.70825918698475	-0.43881920883221	3.26943013309517	n
-2.03131527248772	-0.17855186141396	-0.64881028244888	n
-4.40919047341086	0.41397723134956	0.66069824529295	c
-2.10297150271992	-0.65469479527379	-3.40522723911460	c
-1.08726757795897	1.48829066077773	-4.92328475018861	c
-1.42379555175434	0.51231354579345	4.37343116521934	h
2.68505500957110	0.30569328396713	6.56107397472792	h
6.53275755538753	-1.16042091755003	-0.14319202736277	h
2.60628420843675	-0.98883573466007	-2.65237314021613	h
-5.90845248611823	0.53350730230381	-0.74277470211242	h
-4.88755408857349	-1.06780173896718	2.02413170634924	h
-4.27118463640863	2.23244399118474	1.63572140272320	h
-4.09003761458571	-1.04356706201430	-3.86916911096475	h
-1.08408218389825	-2.41754753939947	-3.80698505402668	h
-0.55245904451614	1.17517914985753	-6.87783875934246	h

-1.22823610666532	3.41198130423732	-4.22821855050404	h
6.36330385547470	-0.50669591673909	4.23352312392222	h

S17

energy: -421.5780259157

1.34844303047303	-0.03311675006653	-3.39988267956332	c
1.93546313776117	0.89124986168489	-0.69383403902409	c
-0.45682239636104	0.22426703166011	0.73029422019469	c
-2.35411841145846	0.02184154253282	-0.90253201221385	n
-1.52381383549992	0.37264919598246	-3.55657257486447	c
-0.43925938877942	-0.05583034725382	3.35437952199209	c
1.88812074870225	-0.27391893764176	4.54362467226610	c
4.07125763521298	-0.38270939944987	3.18293482768889	n
4.20240075322499	-0.10247648275715	0.63869851555128	c
-5.00488244550843	-0.42260284651816	-0.28302951893503	c
-2.14620151061049	-0.20952544467104	4.47096215003338	h
2.08412182461344	-0.50906088737427	6.56540100454838	h
6.02815150054297	-0.34624320426491	-0.24906256946903	h
2.04181699729884	2.98658520781946	-0.74641972502011	h
-5.75769469708133	-1.87204643529305	-1.54690736213479	h
-5.16429489081314	-1.06922554210119	1.66567727940047	h
-6.09310953660676	1.32035766580755	-0.52608246543763	h
-2.02794088661947	2.28022001324953	-4.19264025780801	h
-2.48877881616311	-0.99919763846087	-4.75969979218895	h
1.79411288782067	-2.04370318065572	-3.57730288865258	h
2.37367440231713	1.01375826130595	-4.84908641717448	h

5.68935389753401	-0.79127168353431	4.13108011081115	h

S-TS-12-17

energy: -421.5427033313

1.82964943025253	-0.45450550907649	4.54380666597584	c
-0.39159046619465	-0.03354168871013	3.25880644760683	c
-0.29412090296403	0.54111954103154	0.65662965592244	c
2.14733831798215	1.03830602150542	-0.51838164636407	c
4.30732974646107	0.65917823225509	0.94781073839026	c
4.09900843908920	-0.21366571405272	3.35943646079297	n
-2.25188767603979	0.46270649687773	-0.93695499330125	n
-1.53953016602598	0.10578355736189	-3.63582488213609	c
0.96973432440480	-1.22137584146364	-3.69073022061877	c
-4.88824994582830	0.17116808511737	-0.16331469180759	c
-5.20580748282699	1.14415296451976	1.62593449619615	h
-6.09241591786136	1.03371959959136	-1.59879805954904	h
-5.38798683622571	-1.83062715933813	0.02916599889419	h
-3.03678510814782	-1.02879757391481	-4.50946236012659	h
-1.47785560092694	1.93200280336042	-4.61335849160148	h
2.28218976615669	-0.78983535829901	-5.20664536671440	h
1.03925726881769	-3.13703958944354	-2.95181867254908	h
6.19885073676239	1.06281730473060	0.28197778341057	h
2.27508691127934	2.48433427623230	-1.96978964840071	h
1.88663961965817	-1.04444762487822	6.50166879163418	h
-2.16779908173902	-0.32535228608662	4.23098440418661	h
5.69894462391641	-0.55610053732016	4.35885759015936	h

S18

energy: -483.7873811389

1.44431870954304	-0.68556522238443	-1.43556431372461	c
-0.90790945119832	-0.46866141495059	-0.18589075745742	c
-0.88456119190689	-0.08244188572734	2.44688384692984	c
1.38337706256028	0.01672385622204	3.79870618529578	c
3.67376037672893	-0.24198046272795	2.51931821334545	c
3.71238308282909	-0.58640479080151	-0.10615890572173	c
-3.18008698247447	-0.66258795191320	-1.49886004184067	n
-3.18088223878511	-0.52635979978562	-4.26015268823938	c
-2.71836218081945	2.06520362004597	-5.28051796603368	c
-5.51067908212603	0.08333851755420	-0.23887823818628	c
-2.64697067541938	0.13753174446771	3.47094484956486	h
1.32872164634760	0.40784377034822	6.62619568836999	c
5.44125573932611	-0.15293283003659	3.56595269407522	h
6.19681210078109	-0.83588736172382	-1.48906163393313	c
1.52005119859496	-0.91132386136713	-3.47112203852346	h
-7.07230042863490	-0.14668837012176	-1.56760801207217	h
-5.87058528632694	-1.13381948474640	1.39690036764195	h
-5.47822375940249	2.05998687752589	0.40976037932655	h
-5.01563073666915	-1.23866017063793	-4.91373587311332	h
-1.78254992972301	-1.89536498956055	-4.97597858501517	h
-3.34390786971027	2.58084931532455	-7.16628592827364	h
-1.58123595035213	3.39885977651199	-4.21581516978588	h
5.90580331921129	-0.99249224703224	-3.52978958517051	h
7.41488248862404	0.80203000202107	-1.13482708382118	h

7.23185348327913	-2.51676164272022	-0.85821767304929	h
3.24296304383605	0.54040246106298	7.39421180211027	h
0.31402993982896	2.14505975652067	7.12155790931716	h
0.36367357205809	-1.15989721136806	7.57803255798427	h

S21

energy: -483.8077450345

0.32439011097314	-0.52350603235939	-4.04851550463936	c
0.96804024601963	0.86615449058261	-1.54913901452636	c
-1.43955165906216	0.51694328478995	-0.07374019680682	c
-3.40725477274732	0.47265290690977	-1.76533813183915	n
-2.51904363253328	0.01750513133517	-4.36445040047474	c
-1.49153697997181	0.25366511022219	2.50422677584829	c
0.82176198622184	0.03432147953452	3.86046676899540	c
3.11794125023778	-0.13335737363807	2.48972512127437	c
3.28321453823369	0.10732371596617	-0.09238966976402	c
-5.99177715422895	-0.04229369846065	-1.04375087805558	c
-3.27499955025562	0.07412093890709	3.51051999556814	h
0.79488773255670	-0.16599065732307	6.69438704761194	c
4.84475267081338	-0.50738091455318	3.54898339883450	h
5.72929354411036	-0.07620191524452	-1.50590028479611	c
1.13497467177243	2.90532251655637	-2.04230274039857	h
-7.25267066752421	0.47807873852082	-2.59559930420005	h
-6.29472437947136	-2.05070007414867	-0.57819177688148	h
-6.49287037434962	1.09317304566579	0.61017036786201	h
-2.86784166106000	1.68654178424994	-5.55196344380611	h

-3.53708456562662	-1.58264148319344	-5.20271564888901	h
0.63691272227724	-2.55510933280802	-3.80276963759503	h
1.43480794801994	0.12837707277708	-5.66298602810772	h
7.29843639827282	-0.51225788777129	-0.23190590013708	h
5.66702173962781	-1.54779154623773	-2.97162705845726	h
6.17190583221877	1.70593195972869	-2.48832025951039	h
2.65308032026609	-0.70940535996353	7.42210471308765	h
0.27452996566628	1.63862907858196	7.58482922266011	h
-0.58659628045694	-1.57210497862656	7.34619246714238	h

S-TS-18-21

energy: -483.7733966566

3.43549216383127	0.37924584547939	-0.02388769859219	c
3.21014013774842	-0.27088324187900	2.51903149583302	c
0.86657421540827	-0.24970495845939	3.75972698472008	c
-1.33525735796226	0.29993396574400	2.37701916270268	c
-1.18762116149781	0.85263485191025	-0.19179108773066	c
1.22657235562139	0.95156566700633	-1.42879007782091	c
0.68487645141101	-0.85708053856570	6.53799027821787	c
-3.23388108688378	1.06330459911381	-1.80367512149083	n
-2.68585922300969	0.04949018636576	-4.37701813265512	c
-0.36783701594149	-1.59135506196209	-4.28190539579743	c
5.97308330408403	0.50226817837829	-1.30518112619527	c
-5.78489241986025	0.78349301513883	-0.83856808256042	c
-6.08340608831978	2.08667136696368	0.73810458570549	h
-7.12588167926754	1.26818900383107	-2.33245782325256	h

-6.19102846043630	-1.15370999963677	-0.17861186198701	h
-4.36858807780497	-1.00182327455260	-4.99242222610617	h
-2.41635978470135	1.61055453013577	-5.71986924019020	h
0.91717802320690	-1.57222337591749	-5.88271708529236	h
-0.49857475518885	-3.36901123637768	-3.26065519732204	h
1.43547683409867	2.19121262952231	-3.05635636217231	h
4.90463736958791	-0.72210449858491	3.59408806947868	h
-3.16114438838479	0.19296164166163	3.30988465849333	h
6.20665215732052	2.28793725430173	-2.33324679919985	h
7.51455066059293	0.33419759176688	0.06164797165461	h
6.17949470568965	-1.02544580226472	-2.69517778461730	h
-0.59244523908957	-2.45405567819468	6.87999277511136	h
2.53717896423849	-1.34230244776971	7.31638733174714	h
-0.05913060449100	0.75603978684477	7.60845778931873	h

S19

energy: -634.2895713444

0.86944694764037	-0.58578513368132	-1.45647755698579	c
-1.42260234420535	-0.42634182747864	-0.07889388486062	c
-1.31946784301862	-0.12656288386522	2.55238632908613	c
1.01942139951172	-0.05494414142153	3.78552717724762	c
3.28647099523569	-0.24398368118067	2.46282158708772	c
3.16136696774371	-0.50130620050662	-0.17113953718935	c
-3.73896355481986	-0.59770085457102	-1.31254236586676	n
-3.84713420524100	-0.35772565851752	-4.06212326992324	c
-3.40246360821327	2.26488888164144	-5.01135618717472	c

-6.02677745071832	0.06438758977363	0.06792897398510	c
-3.00682864559036	0.04230050225090	3.69505976764440	h
0.88573074263371	0.22355269855156	6.36152656082979	o
5.12035607435697	-0.18072064460344	3.36512474594384	h
5.46631945371497	-0.64221901980851	-1.35877689133677	o
0.84330448683137	-0.74369550994039	-3.49099548074756	h
-7.64059520845959	-0.16115334050371	-1.19773182238752	h
-6.28701983772780	-1.20659435353779	1.68050416117759	h
-6.00184932984569	2.02143807809578	0.77226138235099	h
-5.71245803184558	-1.03289529344006	-4.66744484174030	h
-2.49328790648544	-1.71140806516436	-4.88769439896625	h
-4.03201667747893	2.82444639620461	-6.88348394246948	h
-2.28583097570080	3.58175357964789	-3.90548751196167	h
5.46270001952379	-0.90294721024663	-4.04295431121553	c
7.44761431849955	-0.98532367289154	-4.59283628631451	h
4.50173185110363	-2.64756548337001	-4.62262172909542	h
4.55080404767951	0.72367470556230	-4.95150619536183	h
3.22209683373150	0.31969178561380	7.70858586117798	c
2.70789066475262	0.53913006262460	9.69259456397005	h
4.30255550753532	-1.43169817823016	7.45249000888161	h
4.36948530885596	1.93530687299264	7.09725509421451	h

S22

energy: -634.3062788953

-0.41406336782331	-0.42113850561515	-4.55069768627204	c
0.14760211091351	1.05912589173313	-2.08454526631126	c

-2.24342354666906	0.62989312655932	-0.60305042937159	c
-4.20771244425945	0.55126231305230	-2.31389086510720	n
-3.28579857349175	-0.04483304892417	-4.87972034669393	c
-2.30745179886973	0.35626522123891	1.96968857479527	c
0.01253675429617	0.23126109118141	3.30965814155601	c
2.35174251888690	0.13279187563243	2.02030465908206	c
2.42400833688756	0.36407460648965	-0.57710972313981	c
-6.75058929896322	-0.12924229596788	-1.57700749467173	c
-4.05880856085265	0.09987274166580	3.00551318715007	h
-0.21941502021743	0.04859498141001	5.90107488009216	o
4.08336522431467	-0.16032434411095	3.07029477223026	h
4.57180080861148	0.25179370118437	-2.03839857509555	o
0.26712139209953	3.09041317612436	-2.61875043023990	h
-8.04730386317959	0.28933321667200	-3.13024684607841	h
-6.91912765665460	-2.14927198724323	-1.08684728517946	h
-7.32114457055226	0.99001326738418	0.06507900532946	h
-3.73312241360067	1.51010562441210	-6.18011019741728	h
-4.21061612766664	-1.75826464275996	-5.59660344250646	h
0.01593121651394	-2.42286854165116	-4.25670415551627	h
0.67198477376169	0.25771060018192	-6.16824126410581	h
2.04705652797688	-0.21919485486901	7.32525190426737	c
1.46053872738183	-0.29647720095415	9.30058011327900	h
3.04761161493614	-1.96951722673553	6.82888375905783	h
3.31179203138704	1.40156415351681	7.03581590488839	h
6.87775110695356	-0.31623305041444	-0.76402621326398	c
8.32642742129427	-0.40246164782432	-2.22809234724119	h

7.35207619546800	1.16735511302146	0.60732676853826	h
6.74923048111717	-2.14160335439011	0.21457089794572	h

S-TS-19-22

energy: -634.2751819307

-1.52758275422365	0.42081761838033	1.81354899889543	c
-1.25266482219166	0.94728900166278	-0.76214194568118	c
1.17754018379703	0.91850594618498	-1.95148164932651	c
3.29882941962892	0.30651036921201	-0.42382599461183	c
3.04206534196506	-0.29484296156986	2.11733473475484	c
0.62990072365363	-0.17311794825421	3.22770096034288	c
-3.23933596735368	1.20754174188178	-2.43436825188849	n
-5.82620801359757	1.09539911467379	-1.53769080770864	c
-2.68377506881741	0.07329341557429	-4.97129669291137	c
-0.39678244935465	-1.59742065098373	-4.80022692519776	c
-6.09223004450863	2.44682449880757	0.00441238574904	h
-7.09551645461136	1.62521218559548	-3.07856542611793	h
-6.36615697489214	-0.80198631082161	-0.85622707814847	h
-4.38657570812171	-0.96956142268051	-5.54407440527196	h
-2.38919078700830	1.58231279983617	-6.36563735320610	h
0.93889362712589	-1.59960544884624	-6.35719163353440	h
-0.59029962284912	-3.37447307788154	-3.78782728273638	h
5.57127487853502	0.40821212060431	-1.66658344865770	o
1.53451805494866	2.16267894162818	-3.54460700148222	h
4.63652261404958	-0.76940131020484	3.30867294907794	h
0.57229083149285	-0.73231152889416	5.76144838823781	o

-3.38922756427919	0.35134319956421	2.65729835489196	h
7.78646283975625	-0.11443400248697	-0.21563318431731	c
9.36687435242467	0.08504646278052	-1.52357474017157	h
7.98664747504015	1.23524107049311	1.34615802978904	h
7.73976992303140	-2.04464809289339	0.54077580809866	h
-1.82026737835319	-0.61369254579424	7.00132072827713	c
-1.44092061834856	-1.05151623743511	8.97946167711305	h
-2.64732708196287	1.28168759162636	6.84617278115797	h
-3.13752895497533	-2.01090453975909	6.21664802458468	h

S20

energy: -603.6867722961

0.73174624953645	-0.90922769895134	-2.47455891789317	c
0.61604260553938	-0.44298724558926	0.15495501497855	c
2.89181950254345	0.10082201091793	1.44468755993311	c
5.14385106257504	0.09899767426055	0.11265026330121	c
5.33567543660881	-0.38622220456545	-2.45958260737647	c
3.05783520768684	-0.87725113139328	-3.66995955704020	c
-1.64950355541387	-0.53755450465033	1.45312561121880	n
-4.03336075749328	-0.71502590120924	0.06557550349384	c
-4.84094325291901	1.70574025799202	-1.14069506001753	c
-1.76565945434724	0.40685703928033	4.03781687471293	c
2.93713328166230	0.50811906724935	3.44736349072180	h
7.12351780656004	-0.36348965028573	-3.45164256956052	h
-0.93727054240480	-1.26286772682266	-3.60037309962058	h
-3.70389986350221	0.21318443912042	4.71421284368086	h

-0.53419910713744	-0.70631347992992	5.27572504850774	h
-1.21198769346938	2.40506431071144	4.17753189371373	h
-5.47742510337783	-1.37313016732974	1.40051232222870	h
-3.85430609559283	-2.24961982015625	-1.33336530160928	h
-6.79470635247079	1.99032814034069	-1.70078880740190	h
-3.44570752159665	3.11421954292770	-1.66344253935116	h
3.10881724662652	-1.33552114414236	-6.19932081577395	f
7.30253090038668	0.61587819222525	1.40957284915350	f

S23

energy: -603.7027018053

-2.84225162387492	0.14728036073205	-3.14061207833808	c
-0.36939103446998	0.91075787114594	-2.05420277189205	c
-0.34044423820374	0.46889432076863	0.76910959485005	c
-2.48614763379941	0.12592510750812	2.19658312877400	c
-4.80369698501892	-0.08472643431710	0.90157516524661	c
-4.99533937426360	-0.17357284925696	-1.74822740163035	c
2.14092153363099	0.42492763883918	1.51525879044666	n
3.85750042598498	-0.00019141604533	-0.64091667492116	c
2.06870412372710	-0.48387031029674	-2.88825099006610	c
2.91511998618548	-0.16357044027774	4.07189135178619	c
-6.95408140742292	-0.39046554048397	2.28941036284487	f
-2.93593612239617	0.06628305107207	-5.72043298311779	f
-2.43224045542303	-0.13984736805843	4.22788031261596	h
-6.80229583801402	-0.51230423872178	-2.65228238886230	h
-0.05062057361455	2.95618533977751	-2.42210074329030	h

4.90000423381953	0.35118632794083	4.31806265220634	h
1.78729861531855	0.93600475491447	5.41083325144085	h
2.68458238948526	-2.18547377345176	4.51526902317980	h
5.09200723793806	-1.62179499405586	-0.26406578235581	h
5.06135861808654	1.66453912347648	-0.94058060098438	h
2.82733164554172	0.21056467819322	-4.67643590003203	h
1.67761647678303	-2.50673120940269	-3.06776531790109	h

S-TS-20-23

energy: -603.6711848966

0.62782457559940	-0.68762110798319	-3.04512403922206	c
-0.09761920088873	-0.82286211234352	-0.51231845285020	c
1.76801482146180	-0.87968194625755	1.47569422321082	c
4.31506498524058	-0.73678045496639	0.71535539006003	c
5.09688768990376	-0.50440479030621	-1.77069982039380	c
3.19964559662383	-0.54796089279773	-3.59812778815825	c
-2.53928504792588	-0.60867683993006	0.35025981102025	n
-4.62980842076295	-0.36558662977573	-1.40949204967083	c
-2.71164551125025	0.74232982079950	2.82419128868061	c
-0.28358936616490	2.13432870226872	3.31012288818815	c
-4.54130221499685	-1.88070475426756	-2.81202656920523	h
-6.40487484933221	-0.55394175695036	-0.37209610334549	h
-4.62803515598794	1.46522915550712	-2.40583374245937	h
-4.34069718111670	2.02324260481988	2.70234206150755	h
-3.10377646043352	-0.61135060465080	4.34775386711436	h
0.42737330713736	2.22999349657978	5.23236102803999	h

0.13107629633854	3.75931310233548	2.12376948713379	h
1.37579281027003	-1.92781885339873	3.19708415266159	h
7.07624193504558	-0.39198925099144	-2.27694525704872	h
-0.73588181908051	-0.56101402976560	-4.56669111357944	h
6.10445059749355	-0.89593622419555	2.55748998672095	f
3.89414261282556	-0.37810663373143	-6.06706924840403	f
